# A bibliometric analysis of immune-related adverse events in cancer patients and a meta-analysis of immune-related adverse events in patients with hepatocellular carcinoma

**DOI:** 10.2478/jtim-2024-0003

**Published:** 2024-07-27

**Authors:** Bengang Wang, Xiangjun Hao, Jinshan Yan, Xin Li, Mingfang Zhao, Tao Han

**Affiliations:** Department of Hepatological surgery, The First Hospital of China Medical University, Shenyang 110001, Liaoning Province, China; School of Life Science and Biopharmaceutics, Shenyang Pharmaceutical University, Shenyang 110001, Liaoning Province, China; Department of Medical Oncology, The First Hospital of China Medical University, Shenyang 110001, Liaoning Province, China

**Keywords:** bibliometrics, hepatocellular carcinoma, immune checkpoint inhibitors, immune-related adverse events, single-arm meta-analysis

## Abstract

**Background and Objectives:**

Immunotherapy has become the standard treatment for hepatocellular carcinoma (HCC), but it carries a risk of immune-related adverse events (irAEs) that can be life-threatening. This study employs bibliometric analysis to understand global scientific research on irAEs in cancer, focusing on characteristics and areas of interest. Additionally, a meta-analysis provides a comprehensive overview of irAEs in HCC patients receiving immune checkpoint inhibitor (ICI)-based therapies.

**Methods:**

We conducted a thorough search of Web of Science Core Collection (WoSCC) publications from 1999 to 2022. R and VOSviewer software were used for analysis. A meta-analysis was performed using data from PubMed, Embase, and the Cochrane Library databases up to March 22, 2022. Trials with HCC patients reporting irAE incidence were included. Quality assessment followed Cochrane risk of bias, Newcastle-Ottawa Scale (NOS), and Methodological Index for Non-Randomized Studies (MINORS). We used random-effects or fixed-effects models based on I2 values. Primary outcomes included any-grade irAEs and grade ≥ 3 irAEs. This review and meta-analysis are registered in PROSPERO as CRD42022318885.

**Results:**

In bibliometric analysis, we included 2946 papers, showing a consistent rise in annual publications on irAEs in cancer research. Frequent keywords were “nivolumab”, “immune checkpoint inhibitor”, and “immune-related adverse event”. “Hepatocellular carcinoma” emerged as a prominent research focus linked to irAEs. We conducted a comprehensive meta-analysis on irAE incidence in HCC patients, including 29 studies. The overall incidence of any-grade irAEs was 61.0% (95% CI 38.5%–81.3%), and grade ≥ 3 irAEs was 13.2% (95% CI 7.9%–19.6%). Treatment-related mortality occurred in 3.1% (95% CI 0.8%–6.3%), with treatment discontinuation at 10.7% (95% CI 6.3%–16.0%). Reactive cutaneous capillary endothelial proliferation (RCCEP) was the most common any-grade irAE, while elevated aspartate aminotransferase (AST) was the most common grade ≥ 3 irAE. Treatment strategies were independently associated with specific irAEs, as indicated by multivariable analysis.

**Conclusion:**

This study provides valuable insights into the current research landscape of irAEs in cancer and ofers a comprehensive overview of irAEs in HCC patients undergoing ICI-based therapy. The relatively high incidence of irAEs and their association with treatment strategies emphasize the need for careful management by clinicians when treating HCC patients. These findings offer significant guidance for optimizing care and treatment for HCC patients.

## Introduction

Hepatocellular carcinoma (HCC) stands as the sixth most prevalent malignancy and ranks third in terms of cancer-related death worldwide.^[1]^ Typically, HCC emerges within the context of chronic liver diseases, with primary factors including HBV and HCV infections, alcohol abuse, and non-alcoholic fatty liver disease.^[2]^ Unfortunately, most HCC cases are diagnosed at an advanced stage, carrying a poor prognosis. Traditional chemotherapy and targeted therapies have demonstrated limited efficacy in improving overall survival for advanced HCC patients.^[3]^ Chemotherapy, with its associated toxicity, often fails to confer survival benefits, while local therapy like radiofrequency ablation (RFA), transarterial chemoembolization (TACE), or selective internal radiotherapy (SIRT) offer only a median survival of 60 months, leaving HCC’s natural survival rate at 35 months.^[4]^

The landscape of cancer treatment has undergone a transformative shift with the advent of immunotherapy, substantially reducing mortality rates, extending overall survival (OS), and prolonging progression-free survival (PFS).^[5]^ Immune checkpoint inhibitors (ICIs) have played a pivotal role in this transformation, bolstering the body’s immune response against cancer cells by inhibiting antibodies such as cytotoxic T-lymphocyte antigen 4 (CTLA-4) and programmed cell death protein 1 (PD-1), or its ligand (PD-L1).^[6]^ However, the remarkable potential of immunotherapy comes with a dual nature. ICIs can trigger various immune-related adverse events (irAEs) by intensifying autoimmunity and disrupting patients’ immune balance. Patients with HCC often harbor additional risk factors, including cirrhosis, immunodeficiency, and inflammation.^[7]^ Consequently, irAEs can impede the utilization and therapeutic efficacy of ICIs, posing the risk of autoimmune-like disorders and even fatal adverse events when combine with other agents.^[8]^ Although individual irAEs have been well-documented, the prevalence and clinical patterns of patients experiencing irAEs affecting multiple organ systems (multisystem irAEs) remain largely unclear.

Bibliometrics, an integral facet of library and information science, employs various qualitative and quantitative techniques to comprehend and systematize previous research findings. This method provides a valuable means of gauging the evolutionary trajectory within a scientific domain and identifying primary research directions through an analysis of databases and literature attributes. Essentially, bibliometrics condenses the vast influx of novel information, conceptual developments, and data into actionable insights *via* specialized analytical methodologies.^[9]^ In the realm of medical research, bibliometric analysis and meta-analysis are potent study designs. Therefore, we have undertaken a bibliometric analysis to summarize the research landscape and pinpoint hotspots in the field of irAEs in HCC. Furthermore, we have conducted a meta-analysis to consolidate the incidence of irAEs in patients with HCC. The purpose of this paper is to improved treatment strategies and provide patients with a more comprehensive understanding and management, ultimately advancing and optimizing immunotherapy for HCC patients.

## Methods

### Bibliometric

#### Data sources and search strategies

**Figure 1:**
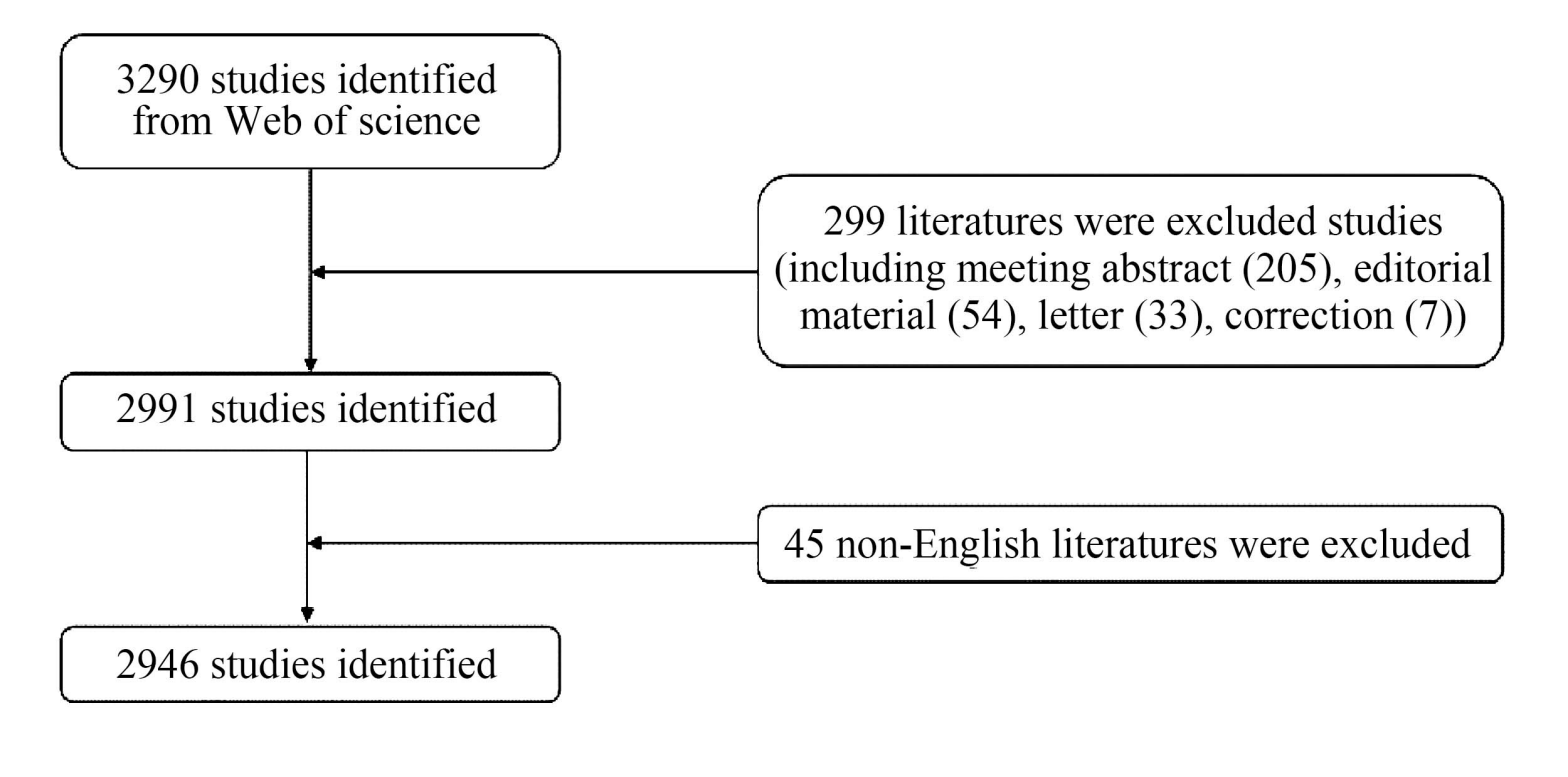
Flowchart of screening process.

We performed bibliometric analyses using the SCI-expanded of WoSCC bibliographic database. To allow for rapid database updates, the literature search was conducted on a single day (September 15, 2022) to avoid bias. In this study, the publication period in this study was set between 1999 and 2022. The search terms were presented as follows: TS = (“Liver Neoplasms” OR “Neoplasms, Hepatic” OR “Neoplasms, Liver” OR “Liver Neoplasm” OR “Neoplasm, Liver” OR “Hepatic Neoplasms” OR “Hepatic Neoplasm” OR “Neoplasm, Hepatic” OR “Cancer of Liver” OR “Hepatocellular Cancer” OR “Cancers, Hepatocellular” OR “Hepatocellular Cancers” OR “Hepatic Cancer” OR “Cancer, Hepatic” OR “Cancers, Hepatic” OR “Hepatic Cancers” OR “Liver Cancer” OR “Cancer, Liver” OR “Cancers, Liver” OR “Liver Cancers” OR “Cancer of the Liver” OR “Cancer, Hepatocellular”) AND TS = (“immune related adverse events” OR “immune related adverse events” OR “irAEs” OR “irAE”). We included only original articles and reviews written in English. Two researchers independently conducted the original data search, and all potential discrepancies were resolved through discussion. Our study ultimately analyzed 2946 articles. The particular screening flowchart is shown in the Figure 1 below.

#### Data collection

Extract the original data from the SCI-expanded database. The information includes title and number of citations, H-index, year of publication, country/region, author, journal, and keywords.

#### Bibliometric analysis

Bibliometric analysis are extensively to decipher the characteristics of the relevant publications in the specific scientific field. The productivity is generally measured by the number of publications (Np), and the impact was assessed by the number of citations without self-citation (Nc), thus, Np and Nc are the two main aspects represent the level of study.^[10,11]^ The H-index unifies productivity and impact by finding a threshold that connects Np and Nc. Although the H-index was initially used to assess individual academic success, it has extended to describe the publication output of country/ region, institution, and journals.^[12]^ In addition, the impact factor (IF) index mainly refers to the latest edition of the Journal Citation Report (JCR), has been widely regarded as indicator of the impact of medical journals.^[13]^ Local Cited Scores (LCS) are important indicator to evaluate an author and considered to be the Nc of an article.^[14]^ The Bibliometrix package^[9]^ in Rstudio (version 2022.02.2) is used for basic bibliometric analysis. To further account for the changes in the number of documents in the year, a fitting polynomial model is used to predict the annual Np. The variable f(x) represents the number of studies per year, and x represents the year of publication. In addition, a bibliometric map was constructed using VOSviewer software to obtain more comprehensive outcome based on co-citation and co-occurrence.^[15]^ If both items were cited by the third item, they were defined as co-cited. The co-occurrence of keywords measures the most frequently occurring keywords in the same document,^[16]^ and analysis of keywords can identify research hotspots for irAEs in cancer patients treated with ICIs.

### Meta-analysis

The protocol of this meta-analysis was registered at PROSPERO, (International Prospective Register of Systematic Reviews, CRD42022318885).

#### Literature search

We searched PubMed, Embase, and Cochrane databases from database inception to March 22, 2022 (Last search time). There was no language restriction in this meta-analysis. The following search terms were included: (hepatocellular carcinoma) AND (immune checkpoint inhibitor OR PD-1 inhibitor OR PD-L1 inhibitor OR CTLA-4 inhibitor OR pembrolizumab OR nivolumab OR atezolizumab OR durvalumab OR avelumab OR cemiplimab OR ipilimumab OR toripalimab OR sintilimab OR camerlizumab OR tislelizumab) AND (immune-related adverse event OR adverse event). The complete search strategy and results are summarized in Table S1.

**Table 1:** The top 10 authorswith the most publications.

Element	H-index	g-index	m-index	TC	Np	PY-start
WOLCHOK JD	21	23	1.4	7506	23	2008
ROBERT C	19	24	1.267	14961	24	2008
WANG Y	18	49	2.571	3164	49	2016
NAIDOO J	17	24	2.125	2131	24	2015
POSTOW MA	17	18	1.545	6863	18	2012
HODI FS	16	21	1.231	12618	21	2010
JOHNSON DB	16	26	2.286	2717	26	2016
LAMBOTTE O	16	25	2.286	3507	25	2016
MATEUS C	15	17	1	3609	17	2008
MICHOT JM	15	20	2.143	2857	20	2016

TC: total citation; Np: number of publications; PY-start: the year that publication starts.

#### Inclusion and exclusion criteria

Studies eligible for inclusion met all the following criteria: (1) participants diagnosed with HCC who were treated with ICIs (PD-1/PD-L1/CTLA-4 inhibitors) alone or in combination with other agents (*e.g*., other drug or surgery); (2) reporting the incidence of irAEs. Exclusion criteria were as follows: (1) fewer than 5 patients in the ICIs group; (2) studies involving multiple advanced tumors; (3) conference abstracts, basic studies, reviews, letters, case reports, meta-analyses; (4) unpublished relevant data from trials still in progress.

#### Data extraction

The following pieces of information were extracted from each study: first author, year of publication, study type, sample size, liver function [Child-Pugh classification and Barcelona Clinic Liver Cancer, (BCLC) classification], Eastern Cooperative Oncology Group (ECOG) performance statuses, ICIs agent, dose, duration of follow-up, number of people with any-grade of irAEs, number of people with grade ≥ 3 irAEs, number of patients with treatment discontinuation due to trAEs, number of treatment-related deaths. Severity was graded according to common terminological criteria for adverse reactions (CTCAE). Those described as AEs of special interest and selected AEs suspected to be potential irAEs were also extracted as irAEs in the present study. The data extraction process was performed independently by the two investigators and any disputes were resolved through discussion.

#### Quality assessments

The two investigators independently conducted the quality evaluation process for the included studies. The six randomized controlled trials (RCTs) were evaluated following the Cochrane risk of bias tool.^[17]^ The evaluation included the following five aspects: sequence generation, allocation concealment, blinding, completeness of outcome data, and other sources of bias. Sixteen cohort studies were evaluated according to the Newscar-Ottawa (NOS) scale,^[18]^ ranging between zero up to nine stars, which contains the following three main aspects: cohort selection, comparability, and outcomes. Other studies (6 single-arm studies, and 1 pilot trial) were evaluated according to the MINORS scale,^[19]^ quality was classified as low (0–8), mediate (9–16), high (17–24).

#### Statistical analysis

The incidence of irAEs was pooled for the included studies in this study and heterogeneity between studies was assessed by Q-test and the I^2^ statistic, and *P* < 0.10 indicated apparent heterogeneity. Heterogeneity was classified as low (*I*^2^ < 25%), mediate (*I*^2^ 25%–75%), and high (*I*^2^ > 75%). A random-effects model was used if *I*^2^ > 50 and a fixed-effects model was used if *I*^2^ < 50. We selected the top 10 incidences of irAE for subgroup analyses, meta-regression, and publication bias test. Subgroup analysis of the incidence and profile of irAEs according to treatment strategy. Multivariate regression analysis was performed to assess risk factors associated with the incidence of irAEs based on treatment strategy (ICIs monotherapy, ICIs combined with other treatments), type of ICIs (anti-PD-1, anti-PD-L1, and anti-CTLA-4), and median patient age (≥ 60, < 60). Publication bias was assessed by funnel plot and Egger’s test. In addition, some irAEs were not subjected to this regression analysis, because of the number of studies less than ten.

All statistical analyses were performed using Stata statistical software version 15.0 (Stata Corp, USA, https://www.stata.com)

## Results

### Bibliometrics

#### Overview

Based on the search strategy, a total of 2946 articles and reviews were retrieved. The total Nc of the retrieved articles was 102,550.26, and the average Nc of each article was 34.81. The H-index for all publications is 140.

#### Annual trend of publication volume

**Figure 2:**
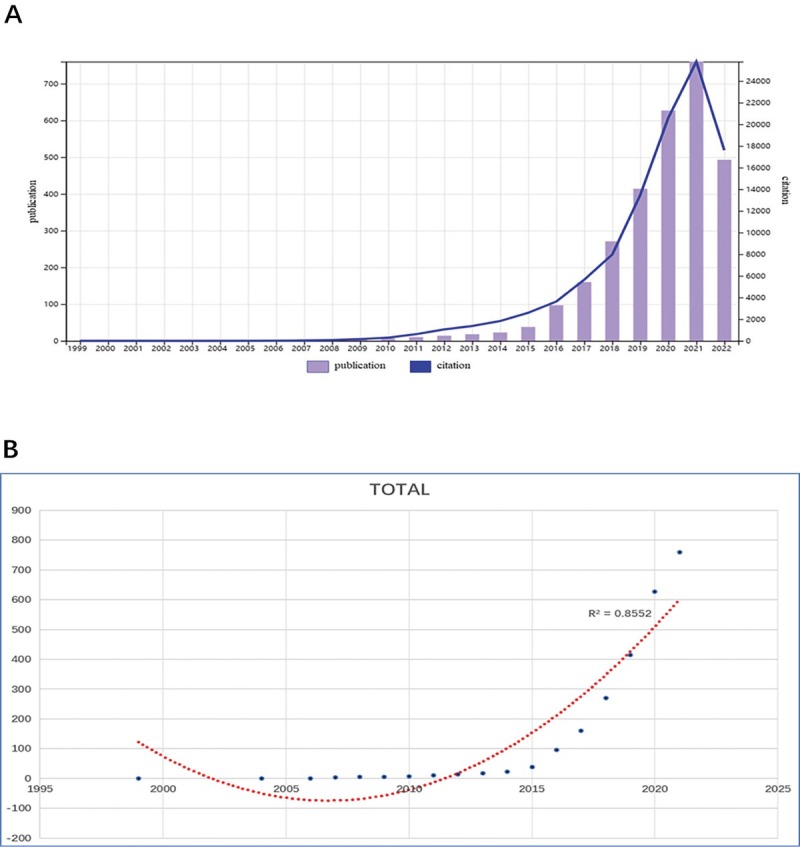
The number of publications and citation by year (A). Polynomial-fitting curve of the annual trend of publication quantity (*R*2 = 0.8552) (B).

**Figure 3:**
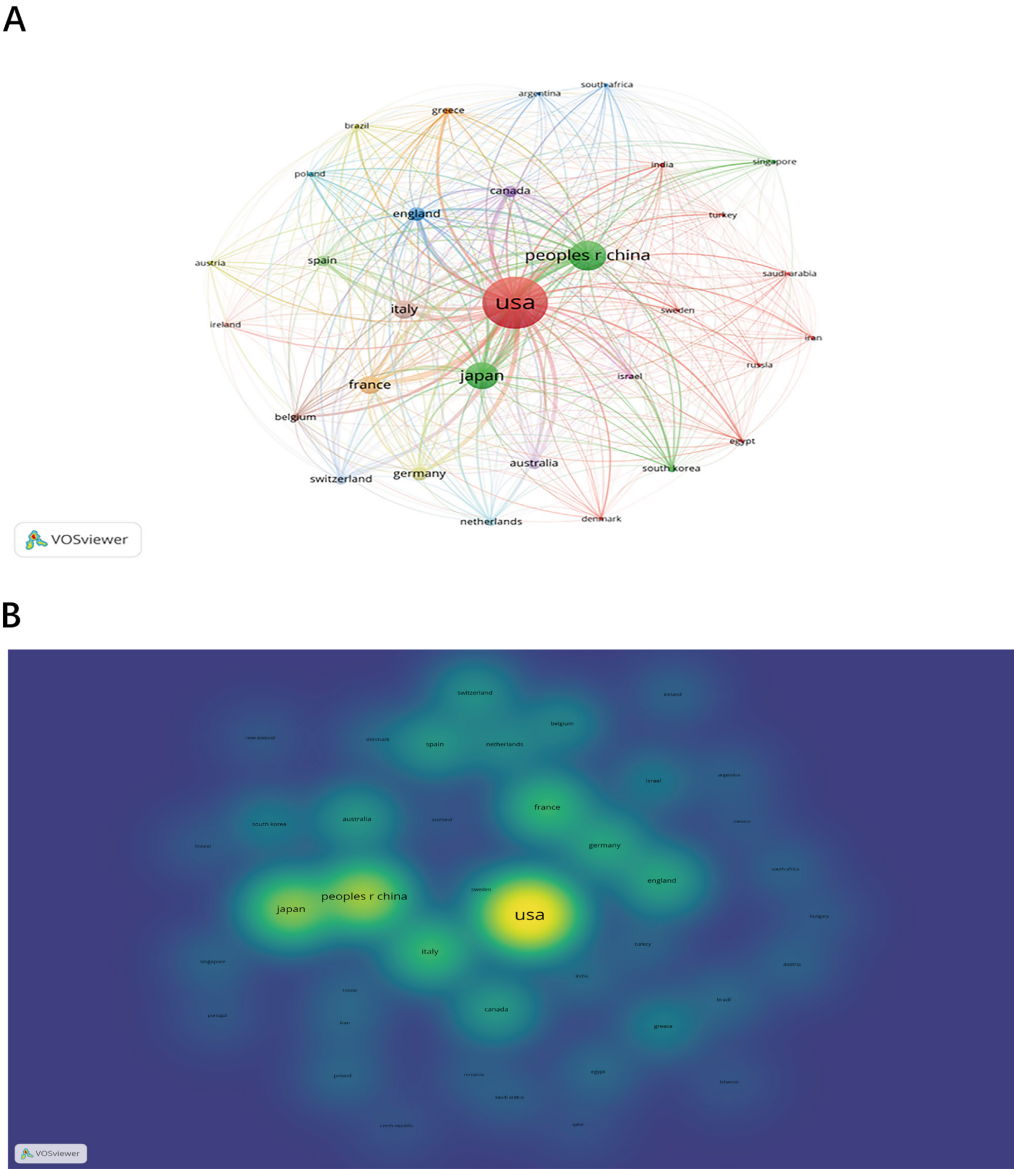
Network visualization map of publications in the countries/regions (A). Density visualization map of publications in the countries/regions (B).

Figure 2A shows the annual Np associated with irAEs in cancer. Overall, the Np rose from 1 in 1999 to a peak of 749 in 2021, despite fluctuations in the rate of growth. The Figure 2B is a polynomial fitting curve of the trend of Np. Np is significantly correlated with the publication year and the correlation coefficient R2 reaches 0.8552. In addition, as shown in the Figure 3A, Figure 3B, the productivity in this field is led by China (1,269,993) and the United States (2,756,866), and there is a big gap productivity between the two, which means that China still has a lot of space for development. Moreover, the cooperation between China, the United States, and Japan is quite close. Overall, these findings indicate that irAEs in cancer treatment has become a key point of attention and has entered a phase of rapid development.

#### Analysis of authors

**Figure 4:**
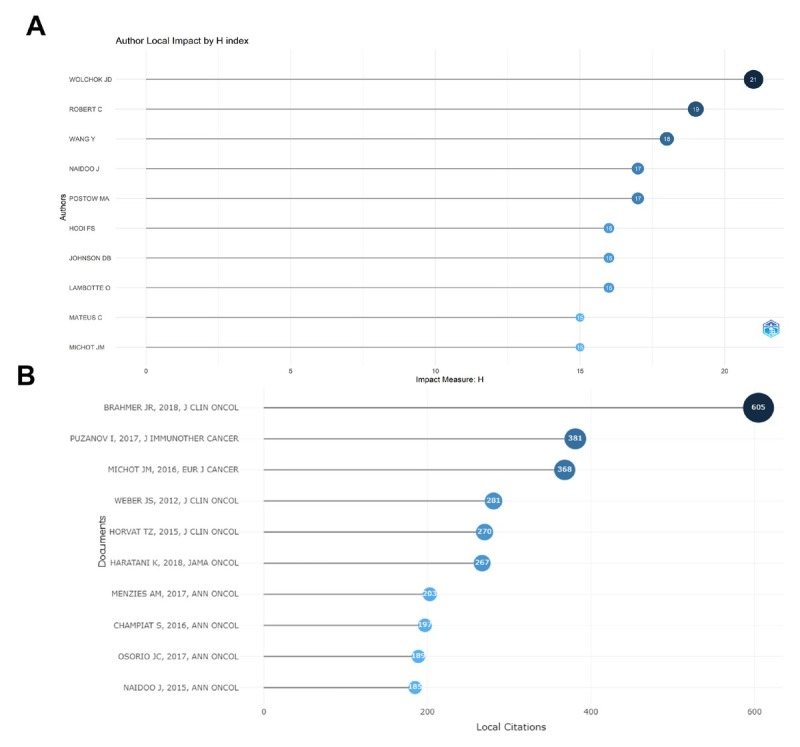
Top ten authors analysis according to H-index (A). Top ten paper analysis of local cited scores (B).

The top 10 authors are listed in Figure 4A according to H-index. Jedd D Wolchok from the United States has the highest H-index, followed by Caroline Robert from France and Yuping Wang from China. In addition, most of these authors are from the United States (4) or France (4), while the other two authors are from China and the United Kingdom, respectively.

#### Analysis of paper local citations (LCS)

The local citations (LCS) for the top 10 articles is presented in Figure 4B. The paper written by BRAHMER JR in 2018 has the highest LCS (605). This article reviews recommended management strategies and provided guidelines for irAEs in patients treated with ICIs. In addition, other articles have elaborated irAEs from different perspectives, mainly focusing on the recommended management strategies of irAEs, the mechanism of irAEs, the efficacy and safety of ICIs, and the characteristics of each irAEs. Among these literatures, most of them were review and published after 2015, which provided a relatively comprehensive description of irAEs.

#### Analysis of journals

As shown in the Table 2, JOURNAL FOR IMMUNOTHERAPY OF CANCER (145 articles, IF: 12.469) was the most productive journal and has the highest IF, while FRONTIERS IN ONCOLOGY (105, IF: 5.738) and FRONTIERS IN IMMUNOLOGY (IF: 8.786) ranked second and third according to productivity. About 25% of the papers published in the top 10 journals (713/24.2%), and had high IF (defined as greater than 3.000). Notably, the EUROPEAN JOURNAL OF CANCER (IF = 10.002) and ONCOLOGIST (IF = 5.837) have a higher citations and H-index, but their productivity were low respectively.

**Table 2:** The top 10 most active journals.

Sources	Articles	H	IF	TC
JOURNAL FOR IMMUNOTHERAPY OF CANCER	145	36	12.469	4961
FRONTIERS IN ONCOLOGY	105	13	5.738	611
FRONTIERS IN IMMUNOLOGY	81	13	8.786	679
CANCERS	76	13	6.575	520
CANCER IMMUNOLOGY IMMUNOTHERAPY	54	18	6.63	1422
EUROPEAN JOURNAL OF CANCER	53	24	10.002	3686
ONCOLOGIST	51	24	5.837	2199
IMMUNOTHERAPY	50	9	4.04	213
JOURNAL OF IMMUNOTHERAPY	49	15	4.912	1014
THORACIC CANCER	49	9	3.223	233

H: H-index; IF: impact factor; TC: total citation.

#### Analysis of research hotspots

**Figure 5:**
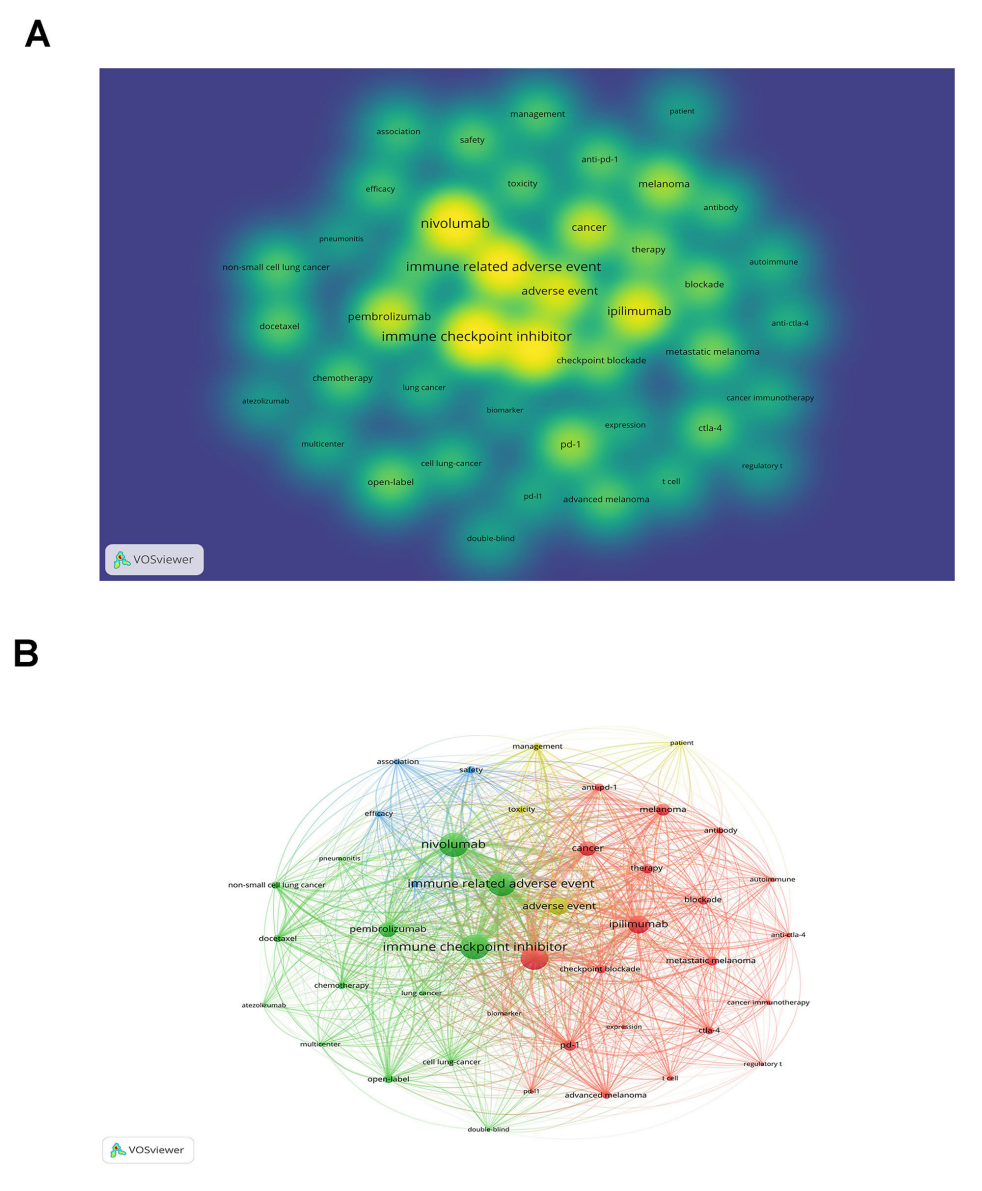
Density visualization map of high frequency keyword (A). Network visualization map of co-occurrence and clustering analysis of the frequent 52 Keywords (B).

A total of 5951 keywords were extracted from the 2496 articles and reviews. A density map was generated for keywords with the co-occurrence greater than 100 times, which includes 43 keywords in the map. As shown in Figure 5A, nivolumab was the most frequent keyword, with 1145 co-occurrences, followed by immune checkpoint inhibitor (1166), immune related adverse event (1113).

Clustering analysis and a network map were performed for co-occurrence keywords by VOSviewer (Figure 5B). Keywords were extracted from titles and abstracts of the 2496 papers. The cumulative frequency of the keywords was calculated and the threshold was set to 40.00% because the keywords with high frequency can accurately reveal the main topic of a field. After calculation, a network map for keywords appearing more than 100 times was generated. There were 43 nodes and links in the network map, and the 43 keywords with high-frequency formed 4 clusters. Cluster 1 was the largest cluster included 21 keywords, mainly related to mechanism of ICIs. Cluster 2 contained 14 keywords, reflected the main focus in clinical trials. Cluster 3 comprised of 4 keywords, mainly related to prognosisof cancer. Cluster 4 included 4 keywords mainly about adverse event and management in the cancer patients. The top frequent occurrences of keywords were “nivolumab,” “immune checkpoint inhibitor”, “immune related adverse event”, and “immune related adverse event” has a strong relationship with almost all of elements, suggesting that the researches related to ICIs in cancer mainly focused on irAEs.

#### Analysis of research hotspots based on types of cancer

ICIs, as a new treatment in this field, have been widely used in various tumors since their advent, which has changed the current treatment mode to a certain extent. However, while ICIs bring significant survival benefits to patients, immunotoxicity to various organs has also become an unavoidable problem in clinical practice. Based on the above bibliometric analysis, in order to further analyze the research progress of irAEs in various solid tumors, keywords of the same type of tumors were combined (*e.g*., In term melanoma and advanced melanoma merged) and selected the top 10 tumor-related keywords, as shown in the Table 3 below.

**Table 3:** Top ten types of cancer according to the frequency.

Cancer	Np
Melanoma	1261
Cancer	785
Cell lung cancer	685
Urothelial carcinoma	54
Prostate cancer	49
Kidney carcinoma	39
Breast cancer	37
Squamous cell carcinoma	36
Hepatocellular carcinoma	32
Bladder cancer	24

Np: number of publications.

**Figure 6:**
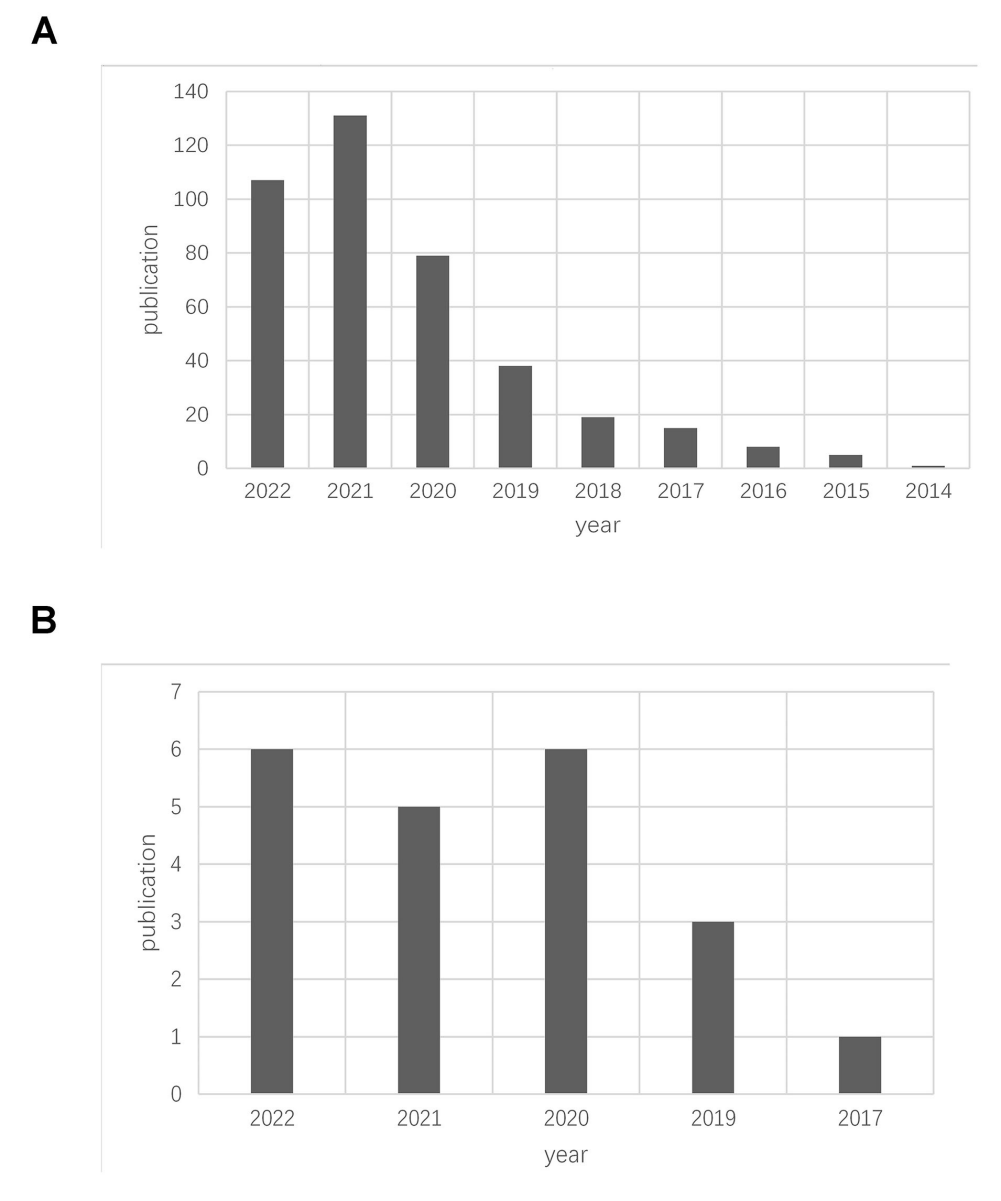
The number of publications by year in ICIs and hepatocellular carcinoma (A). The number of publication by year in immune-related adverse events and hepatocellular carcinoma (B).

After further retrieval of relevant articles on ICIs in HCC and irAEs in HCC, as shown Figure 6A, Figure 6B, the research on ICIs in HCC is superior to that on irAEs in HCC in terms of publication time and number of publications. Since 2017, the number of publications on irAEs in HCC has been increasing year by year. Combined with the above bibliometrics results and the world liver cancer epidemiology data, we found that the study of irAEs in HCC is of great significance. Therefore, we further conducted a meta-analysis on the incidence of irAEs in patients with HCC.

### Meta-analysis

#### Literature search

**Figure 7:**
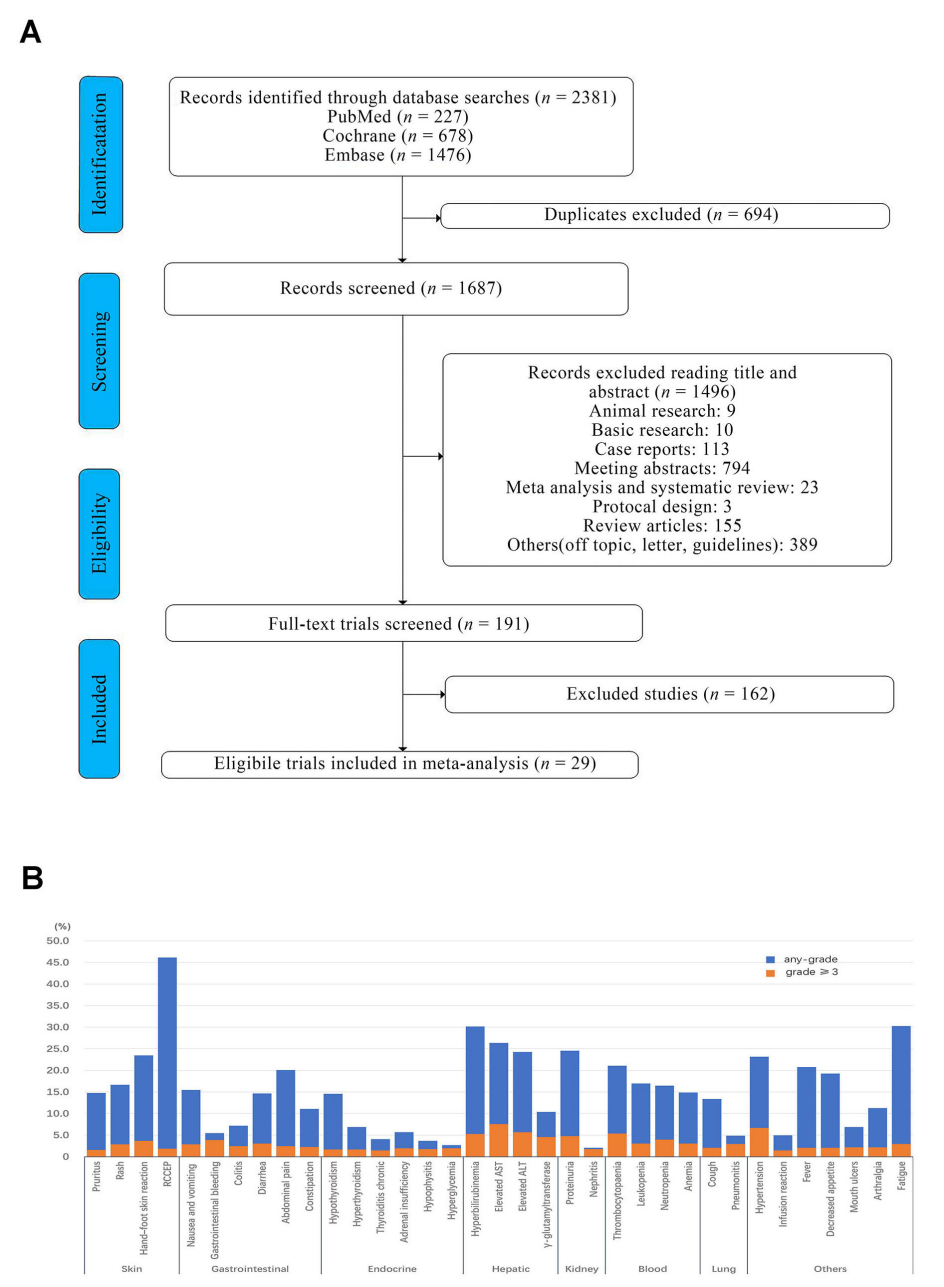
Flow chart of the included studies (A). The incidence of each type of immune-related adverse events (B).

Our search strategy yielded 2381 studies, of which 694 studies were removed due to duplication. 1658 studies were excluded according to the exclusion criteria. Finally, 29 articles including 3066 HCC patients were eligible for the present meta-analysis. The study selection is shown in Figure 7A.

#### Study characteristics

A total of 29 studies were included in our study which contained 16 cohort studies,^[20,21, 22, 23, 24, 25, 26, 27, 28, 29, 30, 31, 32, 33, 34, 35]^ six RCTs,^[36, 37, 38, 39, 40, 41]^ one pilot study,^[42]^ and six single-arm studies.^[43, 44, 45, 46, 47, 48]^ Thirty-five cohorts of 3066 patients were included, with seven cohorts included in the monotherapy cohort and 28 cohorts included in the combination therapy cohort. There were 17 studies involving anti-PD-1 antibody, 10 studies involving anti-PD-L1 antibody, 1 study involving ant-CTLA-4 antibody, and 1 study involving anti-PD-1 with anti-CTLA-4 antibodies. The primary characteristics of the eligible studies are presented in Table 4. Majority of participants were identified as Child-Pugh classes A or B and ECOG performance status of 0–2, and most were at BCLC stage B or C. In addition, most patients had background liver disease, which may increase the incidence of irAEs.

**Table 4:** Characteristics of the included studies.

Author	Year	Size	Type of study	Male/ Female	Age (year)	ICIs	Dose	Follow up (m)
Ando Y.	2021	40	Retrospective Study cohort	30/10	69	Atezolizumab	1200 mg/Kg q3w	4.0
Chen S.	2021	70	Retrospective Study cohort	37/33	58	Pembrolizumab	200 mg d1/d21	27.0
Chuma M	2022	94	Retrospective Study cohort	73/21	73	Atezolizumab	1200 mg/Kg q3w	4.8
D’Alessio A	2022	202	Retrospective Study cohort	173/29	69	Atezolizumab	1200 mg/Kg q3w	9.0
De Castro T	2022	147	Retrospective Study cohort	125/22	68.7	Atezolizumab	1200 mg/Kg q3w	6.2
Guo Y	2022	20	Retrospective Study cohort	19/1	50.3	Camrelizumab	200 mg q3w	9.0
Hayakawa Y	2021	52	Retrospective Study cohort	42/10	73	Atezolizumab	1200 mg/Kg q3w	7.4
He MK	2021	71	Retrospective Study cohort	59/12	NA	Toripalimab	240 mg d1; 240 mg q3w	NA
Huang J	2022	58	Retrospective Study cohort	51/7	54	Sintilimab	200mg q3w	NA
Iwamoto H	2021	51	Retrospective Study cohort	45/6	71	Atezolizumab	1200 mg/Kg q3w	2.9
Ju S	2022	56	Retrospective Study cohort	46/10	52	Camrelizumab	250 mg qd	13.5
Ju S	2022	52	Retrospective Study cohort	44/8	55	Camrelizumab	250 mg qd	13.5
Liu Q	2021	35	Retrospective Study cohort	32/3	53	Camrelizumab	200 mg q2w	8.8
Sho T	2021	58	Retrospective Study cohort	49/9	72	Atezolizumab	1200 mg/Kg q3w	1.5
Xia J	2021	27	Retrospective Study cohort	23/3	60	Nivolumab	480 mg q4w	NA
Yuan G	2020	63	Retrospective Study cohort	58/5	48,7	Camrelizumab	200 mg q3w	12.6
Yang F	2021	31	Single-arm study	25/6	NA	Camrelizumab	200 mg q3w	9.0
Kudo M	2021	22	Single-arm study	20/2	68.5	Avelumab	10 mg/kg q2w	NA
Zhu AX	2018	104	Single-arm study	86/18	68	Pembrolizumab	200 mg q3w	12.3
Liu J	2021	22	Single-arm study	17/5	57.7	Camrelizumab	200 mg q3w	NA
Cao F	2021	52	Single-arm study	45/7	NA	Sintilimab	200 mg d1/d21	12.5
Wang JH	2022	48	Single-arm study	38/10	62	Atezolizumab	1200 mg/Kg qw	9.5
Duffy AG	2017	32	Pilot study	28/4	61	Tremelimumab	3.5 mg/kg q4w	18.8
Finn RS	2020	278	RCT	226/52	67	Pembrolizumab	200 mg q3w	13.8
Lee MS	2020	104	RCT	84/20	62	Atezolizumab	1200 mg/Kg q3w	12.4
Lee MS	2020	60	RCT	54/6	60	Atezolizumab	1200 mg/Kg q3w	6.6
Lee MS	2020	59	RCT	49/10	63	Atezolizumab	1200 mg q3w	6.7
Qin S	2020	109	RCT	98/11	48	Camrelizumab	3 mg/kg q2w	12.5
Qin S	2020	108	RCT	98/10	50	Camrelizumab	3 mg/kg q3w	12.5
Ren Z	2021	380	RCT	334/46	53	Sintilimab	200 mg mg/kg q3w	10.0
Yau T	2020	50	RCT	43/7	61	Nivolumab; Ipilimumab	1 q2mg/w; kg 3 mg/q3w; kg 240 q3w mg	30.7
Yau T	2020	49	RCT	37/12	65	Nivolumab; Ipilimumab	1 q2mg/w; kg 3 mg/q3w; kg 240 q3w mg	30.7
Yau T	2020	49	RCT	40/9	58	Nivolumab;Ipilimumab	3mg/kg q2w; 1mg/kg q6w	30.7
Yau T	2022	367	RCT	NA	65	Nivolumab	240mg q2w	15.2
Zhang S	2021	46	Prospective Study cohort	29/17	57.16	Camrelizumab	200mg d1/d21	12.0

ICI: immunecheckpointinhibitor.

**Table 5:** The results of the subgroup analysis.

	Any grade				Grade ≥ 3		
	
irAEs	Combination	ICI	*P* value	irAEs	Combination	ICI	*P* value
RCCEP	34.5%	67.0%	0.007	Elevated AST	7.3%	5.3%	0.322
Fatigue	29.3%	28.4%	0.935	Hypertension	6.6%	0.4%	0.000
Hyperbilirubinemia	33.1%	4.8%	0.000	Elevated ALT	5.2%	2.0%	0.077
Elevated AST	29.5%	17.1%	0.053	Thrombocytopaenia	4.1%	1.6%	0.037
Elevated ALT	26.0%	17.8%	0.303	Hyperbilirubinemia	4.6%	1.0%	0.040
Proteinuria	25.2%	14.1%	0.212	Proteinuria	4.2%	0.7%	0.000
Hypertension	27.6%	0.9%	0.000	Inceased γ-glutamyltransferase	4.1%	1.8%	0.120
Hand-foot skin reaction	25.7%	1.1%	0.000	Neutropenia	3.0%	3.2%	0.966
Thrombocytopaenia	22.2%	13.6%	0.203	Gastrointestinal bleeding	-	-	-
Fever	-	-	-	Hand-foot skin reaction	2.9%	0.3%	0.001

irAEs: immune-related adverse events; ICI: immune checkpoint inhibitor; Combination ICIs: combined with other treatments (chemotherapy,targeted therapy, surgery).

**Table 6:** Multivariateregression analyses of the top tenimmune-related adverse events.

Any-grade irAEs	Combination type	ICI type	Age	Grade ≥ 3 irAEs	Combination type	ICI type	Age
Fatigue	0.122	0.139	0.218	Hand-foot skin reaction	0.046	0.270	0.448
Hand-foot skin reaction	0.139	0.515	0.834	Hypertension	0.009	0.723	0.758
Hypertension	0.000	0.716	0.818	Proteinuria	0.002	0.254	0.866
Fever	-	0.909	0.678	Thrombocytopaenia	0.146	0.623	0.601
Proteinuria	0.087	0.186	0.109	Elevated AST	0.685	0.893	0.484
Thrombocytopaenia	0.849	0.871	0.765	Elevated ALT	0.001	0.053	0.015
Elevated AST	0.472	0.757	0.347	Gastrointestinal bleeding	-	-	-
Elevated ALT	0.489	0.810	0.810	Neutropenia	-	-	-
Hyperbilirubinemia	-	-	-	increased γ-glutamyltransferase	-	-	-
RCCEP	-	-	-	Hyperbilirubinemia	-	-	-

irAEs: immune-related adverse events; ICI: immune check point inhibitor.

**Table 7:** Publication bias of the top ten immune-related adverse events.

Any Grade irAEs	*P* value	Grade ≥ 3 irAEs	*P* value
Fatigue	0.001	Hand-foot skin reaction	0.001
Hand-foot skin reaction	0.003	Hypertension	0.609
Hypertension	0.210	Proteinuria	0.383
Fever	0.628	Thrombocytopaenia	0.004
Proteinuria	0.000	Elevated AST	0.095
Thrombocytopaenia	0.089	Elevated ALT	0.165
Elevated AST	0.828	Gastrointestinal bleeding	-
Elevated ALT	0.841	Neutropenia	-
Hyperbilirubinemia	-	Increased γ-glutamyltransferase	-
RCCEP	-	Hyperbilirubinemia	-

irAEs: immune-related adverse events; AST: aspartate aminotransferase; ALT: alanine aminotransferase; RCCEP: reactive cutaneous capillary endothelial proliferation.

#### Incidence of irAE

Of the 29 included studies, a total of 6 studies reported overall any-grade irAEs and 7 studies reported overall grade ≥ 3 irAEs, with incidences of 61.0% (95% CI 38.5%–81.3%, *I*^2^ = 98.02%; Figure S1), 13.2% (95% CI 7.9%–19.6%, *I*^2^ = 87.11%; Figure S2). Eighteen studies reported trAEs leading to treatment discontinuation, with an incidence of 10.7% (95% CI 6.3%–16.0%, *I*^2^ = 89.08%; Figure S3). Deaths due to trAEs were reported in 17 studies, with an incidence of 3.1% (95% CI 0.8%–6.3%, *I*^2^ = 86.74%; Figure S4). The incidence of each type of irAEs is shown in the Figure 7B: the most common any-grade irAEs were RCCEP (44.8%), fatigue (28.9%), hyperbilirubinemia (28.8%), elevated AST (25.0%), proteinuria (23.2%), elevated alanine aminotransferase (ALT) (22.9%), hand–foot skin reaction (22.1%), hypertension (21.8%), thrombocytopenia (19.7%), and fever (19.4%). The most common grade ≥ 3 irAEs were elevated AST (6.2%), hypertension (5.3%), elevated ALT (4.3%), thrombocytopenia (4.0%), hyperbilirubinemia (3.9%), proteinuria (3.4%), increased γ-glutamyltransferase (3.2%), neutropenia (2.6%), gastrointestinal bleeding (2.5%), and hand–foot skin reaction (2.3%).

#### Organ-specific irAEs

With regards to organ-specific irAEs, we summarized the incidence of irAEs in HCC patients treated with ICIs involving the skin, gastrointestinal tract, endocrine, liver, kidney, hematological system, lung, and others. The most common any-grade irAEs involved the skin (13.4%– 44.8%). The grade ≥ 3 irAEs mostly occurred in the liver (3.2%–6.2%). The detailed information is presented (Figure S5-74)

#### Skin

There were four categories of skin-related irAEs, including pruritus, rash, hand-foot skin reaction, and RCCEP. RCCEP was the most common of the any-grade irAEs, with an incidence of 44.8% (95% CI 22.2%-68.7%, *I*^2^ = 95.14%), and hand-foot skin reaction was the most common of the grade ≥ 3 irAEs, with an incidence of 2.3% (95% CI 0.7%–1.4%, *I*^2^ = 69.22%).

#### Gastrointestinal tract

A total of six classes of irAE were associated with the gastrointestinal system, including nausea and vomiting, gastrointestinal bleeding, colitis, diarrhea, abdominal pain, and constipation. Of these, abdominal pain was the most common of the any-grade irAEs, with an incidence of 18.7% (95% CI 12.7%–25.4%, *I*^2^ = 81.94%), and gastrointestinal bleeding was the most common of the grade ≥ 3 irAEs, with an incidence of 2.5% (95% CI 1.3%–3.9%, *I*^2^ = 0%).

#### Endocrine

There were six classes of irAE that related to endocrine, including hypothyroidism, hyperthyroidism, thyroiditis chronic, hypophysitis, adrenal insufficiency, and hyperglycemia. Of these, hypothyroidism was the most common of the any-grade irAEs, with an incidence of 13.2% (95% CI 9.9%–16.8%, *I*^2^ = 69.05%), and adrenal insufficiency and hyperglycaemia were the most common of the grade ≥ 3 irAEs, both with an incidence of 0.6% (95% CI 0%–1.6%, *I*^2^ = 25.74%) (95% CI 0.1%–1.3%, *I*^2^ = 0%).

#### Liver

There were four classes of liver-related irAE, including hyperbilirubinemia, elevated AST, elevated ALT, and increased γ-glutamyltransferase. Of these, hyperbilirubinemia was the most common 28.8% (95% CI 17.0%–42.2%, *I*^2^ = 92,49%) of any-grade irAEs, and elevated AST was the most common of grade ≥ 3 irAEs with an incidence of 6.2% (95% CI 4.0%–8.9%, *I*^2^ = 73.19%).

#### Kidney

There were two categories of kidney-related irAE, including proteinuria and nephritis. Of these, proteinuria was the most common in both any-grade irAEs and grade ≥ 3 irAEs, with an incidence of 23.2% (95% CI 17.4–29.6%, *I*^2^ = 86.62%) and 3.4% (95% CI 2.5%–4.4%, *I*^2^ = 33.24%), respectively.

#### Hematological system

There were four categories of hematological-related irAE, including: thrombocytopenia, leucopenia, neutropenia, and anemia. Thrombocytopenia was the most common irAE in both any grade and grade ≥ 3 irAE, with incidences of 19.7% (95% CI 10.9%–30.2%, *I*^2^ = 93.39%) and 4.0% (95% CI,2.8%–5.2%, *I*^2^ = 41.39%), respectively.

#### Lung

Two categories of lung-related irAE including: cough and pneumonia were summarized. Of these, any-grade irAEs were most common in cough, with an incidence of 12.0% (95% CI 6.2%–19.2%, *I*^2^ = 66.60%), and grade ≥ 3 irAEs were most common in pneumonia, with an incidence of 1.6% (95% CI 0.9%–2.5%, *I*^2^ = 0%).

#### Other

Other irAEs included hypertension, infusion reactions, fever, decreased appetite, mouth ulcers, and fatigue. Of the any-grade irAEs, fatigue was the most common irAE, with an incidence of 28.9% (95% CI 22.8%–35.3%, *I*^2^ = 86.23%), and of the grade ≥ 3 irAEs, hypertension was the most common irAE, with an incidence of 5.3% (95% CI 2.6%–8.6%, *I*^2^ = 85.47%).

### Subgroup analyses

To explore whether the incidence of irAEs was related to treatment strategy, we selected the top 10 incidences of irAE for subgroup analyses (Table 2), divided into ICIs monotherapy group and ICIs combined with other treatments (chemotherapy, targeted therapy, surgery) (Figure S75-S92). Of these, the incidence of any-grade irAEs correlated with treatment strategy included RCCEP, hyperbilirubinemia, hypertension, and thrombocytopenia. Incidences in grade ≥ 3 irAE associated with treatment strategies included hypertension, thrombocytopaenia, hyperbilirubinemia, proteinuria, and hand-foot skin reaction. The incidence of these irAEs was significantly higher in the combination therapy group than in the monotherapy group, except for RCCEP.

**Figure 8:**
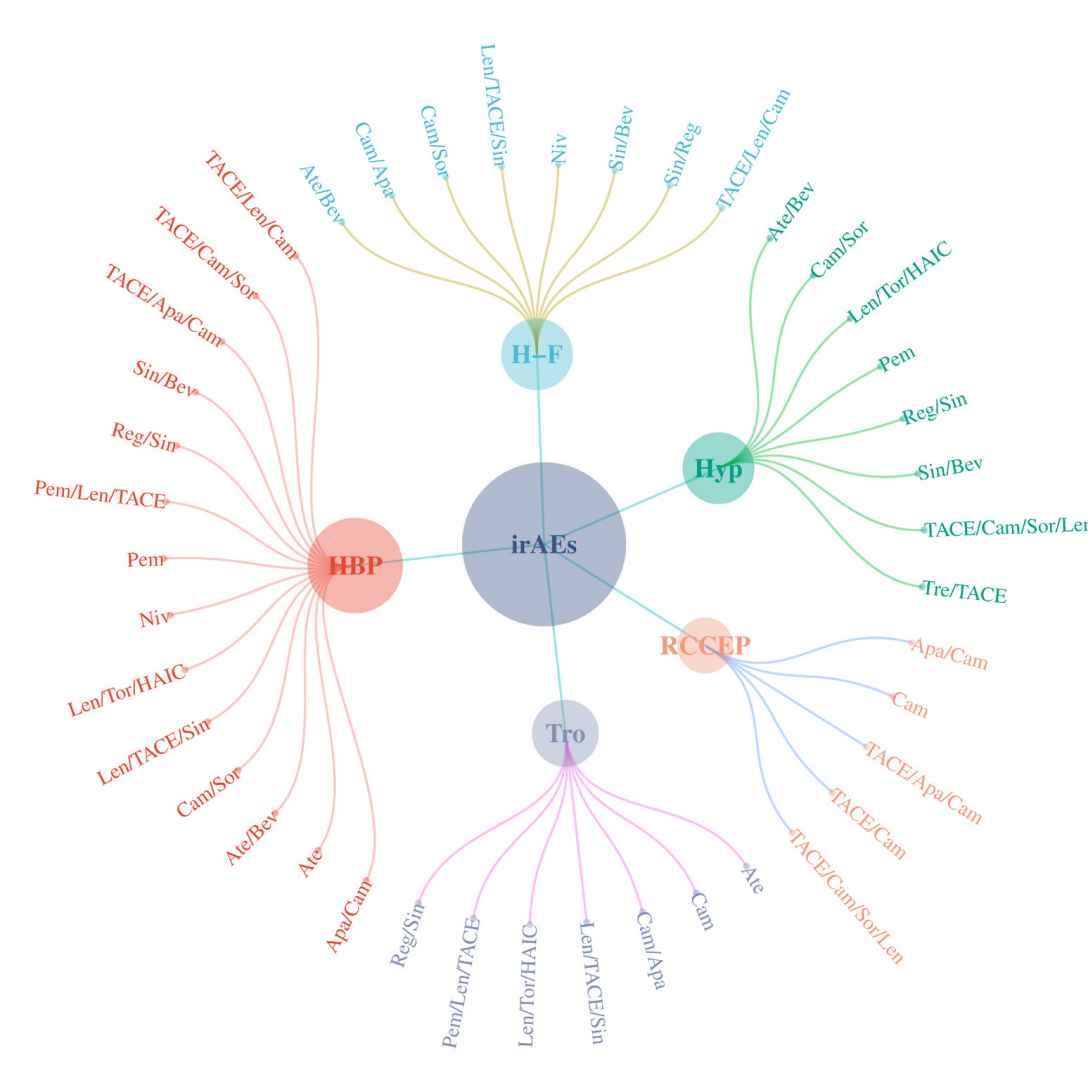
Treatment regimens options were classified according to immune-related adverse events.

IrAEs related to treatment were classified according to their corresponding treatment regimens based on the subgroup analysis (Figure 8). We found that carrilizumab appeared in all treatment regimens with RCCEP, the treatment regimens of the above irAEs were mostly ICIs-targeted combination therapy and the type of ICIs was mainly PD-1, and bevacizumab was often seen in the treatment regimens of thrombocytopenia and hypertension.

### Multivariate regression analysis

Among the any-grade irAEs, only the incidence of hypertension was significantly associated with treatment strategy (*P* < 0.05), while the incidence of the remaining category of irAEs was not significantly associated with treatment strategy, median age, or type of ICIs. The incidences of hypertension, elevated ALT, proteinuria, and hand-foot skin reaction were all significantly associated with treatment strategy in grade ≥ 3 irAEs (*P* < 0.05), and the incidence of elevated ALT was significantly associated with the median age of the patient (*P* < 0.05). Furthermore, the studies that reported any-grade fever were all combination therapies, so only regression analyses based on type of ICIs and median age value could be performed.

Treatment strategy was significantly associated with the risk of irAEs, especially some grade ≥ 3 irAEs, while type of ICIs and age were not significantly associated with irAEs.

### Quality assessment

Six RCTs were assessed using the Cochrane risk of bias tool. Of the six studies, five studies were assessed as high risk of bias (Figure S93). Sixteen cohort studies were evaluated according to the NOS scale and scored 5–8 (Table S2). Seven non-randomized studies were assessed using the MINORS index score ranging from 9 to 19 points, which was of relatively high quality (Table S3). Overall, the quality of the included studies was moderate or high.

### Publication bias

We used funnel plots (Figure S94-S107) and egger’s test (Table 4) to detect publication bias for the pooled results of the top 10 incidence of irAEs, where hyperbilirubinemia and RCCEP in any-grade irAEs and gastrointestinal bleeding, neutropenia, increased γ-glutamyltransferase, and hyperbilirubinemia in grade ≥ 3 irAEs did not perform these tests due to the number of included studies was less than ten. According to the results of the egger’s test, there was publication bias for fatigue, hand-foot skin reaction, and proteinuria in any-grade, as well as hand-foot skin reaction, and thrombocytopenia in grade ≥ 3 irAEs. Possible reasons for this result include the large heterogeneity between the pooled studies. In addition, there may be some subjectivity in the definition of irAEs by the investigators.

## Discussion

Our study, through bibliometric analysis and literature visualization, provides valuable insights into the developmental trends and research hotspots within this field. It represents a significant and comprehensive meta-analysis of irAE incidence and spectrum in HCC patients treated with ICIs. In comparison to previous studies,^[49,50, 51]^ our meta-analysis includes a larger number of studies and covers a wider range of irAE types. Identifying these irAEs at an earlier stage can significantly slow their progression to higher grades of adverse events, thereby enabling patients to safely continue treatment.^[52]^

In this study, we firstly conducted a bibliometric analysis to investigate the developmental trends and research hotspots concerning irAEs in HCC. Our analysis of countries/regions highlighted China and the United States as leaders in the overall development of this field, with a close connection between them. Among the top ten researchers, a Chinese researcher (WANG Y) ranks among the top three. However, most of these top researchers are from the United States and France, indicating that Chinese researchers are actively pursuing research hotspots in this field. Furthermore, we examined the most influential journals in this domain and identified papers with the highest LCS (citation score). Our results revealed that the “JOURNAL FOR IMMUNOTHERAPY OF CANCER” was the most prolific journal and boasted a high IF in this field. Generally, the top ten journals all had high IFs, with approximately 25% of articles in this field published in these journals. This suggests that researchers investigating irAEs in cancer patients have a favorable environment for publishing high-quality articles.

Moreover, to provide valuable guidance for clinical practice, we conducted co-occurrence and cluster analyses on keywords, revealing that irAEs hold significant research value within the realm of cancer. IrAEs have the potential to affect virtually every organ system and are considered a notable challenge associated with this class of therapeutic agents.^[53]^ Among numerous keywords, we categorized different types of cancer based on their frequency, enabling us to track research hotspots more effectively. Given our statistical findings and the unique prominence of HCC in China, along with our research team’s outstanding achievements in this area, we observed that HCC ranks relatively low among the top ten research topics and offers ample room for further investigation. Consequently, we conducted separate searches for “ICIs in HCC” and “irAEs in HCC.” Notably, the study of the combination of HCC and irAEs emerged later than that of HCC and ICIs. Thus, we infer that there is considerable research potential in this direction, which motivated us to conduct a comprehensive meta-analysis to determine the incidence of irAEs in HCC patients using ICI drugs. The results of our meta-analysis offer a comprehensive overview of the incidence of immune-related adverse events (irAEs) following treatment with immune checkpoint inhibitors (ICIs) in patients with hepatocellular carcinoma (HCC). When examining the overall incidence of irAEs, most cases fall into the grade 1–2 category, with only a minority of patients experiencing severe irAEs. As per a previous meta-analysis,^[52]^ specifically a retrospective review by Das *et al*., a higher incidence of irAEs is associated with better outcomes in HCC patients receiving ICIs.

Notably, we categorized irAEs based on the affected organ or system and discovered that the incidence of irAEs varies across organs. Any-grade irAEs were most common in the skin system, with skin-associated irAEs being particularly prevalent with RCCEP, albeit progressing to severe irAEs in only 0.5% of patients. RCCEP, a common irAE linked to camrelizumab, typically resolves spontaneously upon treatment completion.^[54]^ Grade ≥ 3 irAEs were most frequent in the liver, with elevated AST levels being the primary manifestation. This elevation may be attributed to pre-existing hepatitis B or C among HCC patients before commencing ICI treatment, warranting close clinical monitoring. Grade ≥ 3 irAEs can lead to treatment discontinuation or even patient fatality, demanding heightened clinical attention. Most importantly, our research indicated that irAEs related to the endocrine system had the lowest incidence, both in any-grade and grade ≥ 3 irAEs.

However, a prior review^[55]^ suggests that endocrine irAEs are among the most common. Due to their nonspecific symptoms, clinicians must possess a thorough understanding of each endocrine irAE’s clinical characteristics for proper management, as their development can have life-threatening consequences. In this study, we conducted subgroup analysis on the ten most common irAEs based on treatment strategies. The incidence of certain irAEs varied depending on the treatment strategy, with combination therapy demonstrating higher rates compared to ICIs monotherapy. Subgroup analysis allowed us to classify irAEs related to treatment according to their respective treatment regimens and incidence rates. Notably, camrelizumab appeared across all treatment regimens involving RCCEP, while the treatment regimens for the mentioned irAEs were predominantly ICIs-targeted combination therapies, mainly involving PD-1 inhibitors. Bevacizumab was frequently incorporated into treatment regimens for thrombocytopenia and hypertension. These findings are expected to guide clinicians in the future utilization of ICIs for liver cancer patients.

Nevertheless, this study has certain limitations. In the bibliometrics section, we restricted our analysis to articles and reviews written in English and indexed in SCI-expanded, potentially excluding relevant research. In the meta-analysis section, we faced limitations such as a small number of included papers, making it difficult to perform regression analysis and publication bias tests for some irAEs. Most studies were retrospective and single-arm, with fewer randomized controlled trials (RCTs), potentially impacting the overall quality of the articles. This single-arm meta-analysis introduced a high degree of heterogeneity, and partial publication bias may have affected the credibility of our results. Additionally, the assessment of irAEs involves investigator subjectivity, making strict standardization challenging, especially when determining whether specific adverse events are immune-related.

## Conclusion

Bibliometric analysis reveals a rapid evolution in research on irAEs among cancer patients, with ample opportunities for further investigation into irAEs in HCC patients. Our meta-analysis provides a comprehensive overview of irAEs associated with ICIs in HCC patients. Our findings indicate that ICIs generally result in manageable irAEs in HCC patients, though the incidence and profiles of these events vary with different treatment strategies. Notably, combination therapies exhibit a higher incidence of irAEs, underscoring the importance of early diagnosis for timely intervention with ICIs to mitigate severity and improve patient outcomes. However, it is essential to approach these conclusions cautiously, as more high-quality and adequately powered randomized controlled trials are required to further solidify our findings.

## Supplementary Information

Figure S1-S74: Forest plots of the meta-analysis of incidents of irAEs.

Figure S75-S92: Forest plots of the subgroup analysis of the incidence of irAEs.

Figure S93: Assessment of risk of bias of included RCTs

Figure S94-S107: Funnel plot for studies of irAEs.

Table S1: Search algorithm and results.

Table S2-S3: Risk of bias and quality assessment of cohort studies and non-randomized studies.

Supplementary information of this article can be found online at www.intern-med.com.

## Supplementary Material

Supplementary Material
