# Supplementary material for: A bibliometric analysis of immune-related adverse events in cancer patients and a meta-analysis of immune-related adverse events in patients with hepatocellular carcinoma
Source: J Transl Int Med. 2024 Jul 27;12(3):225–43. doi: 10.2478/jtim-2024-0003 (PMC11285746; doi:10.2478/jtim-2024-0003)
Supplement: Supplementary file 1 — Supplementary Material [file jtim-2024-0003_sm.pdf]

A bibliometric analysis of immune-related adverse events in cancer patients and a meta-analysis of immune-related adverse events in patients with hepatocellular carcinoma

Bengang Wang[1], Xiangjun Hao[2], Jinshan Yan[3], Xin Li[3], Mingfang Zhao[2*], Tao Han[1*]

[1]Department of Hepatological surgery, The First Hospital of China Medical University, Shenyang 110001, Liaoning Province, China

[2]School of Life Science and Biopharmaceutics, Shenyang Pharmaceutical University, Shenyang 110001, Liaoning Province, China

[3]Department of Medical Oncology, The First Hospital of China Medical University, Shenyang 110001, Liaoning Province, China

***Correspondence author:** Tao Han, Department of Medical Oncology, The First Hospital of China Medical University, 155 North Nanjing Street, Heping District, Shenyang 110001, Liaoning Province, China. E-mail: than1984@sina.com; Mingfang Zhao, Department of Medical Oncology, The First Hospital of China Medical University, 155 North Nanjing Street, Heping District, Shenyang 110001, Liaoning Province, China. E-mail: zhaomf618@126.com

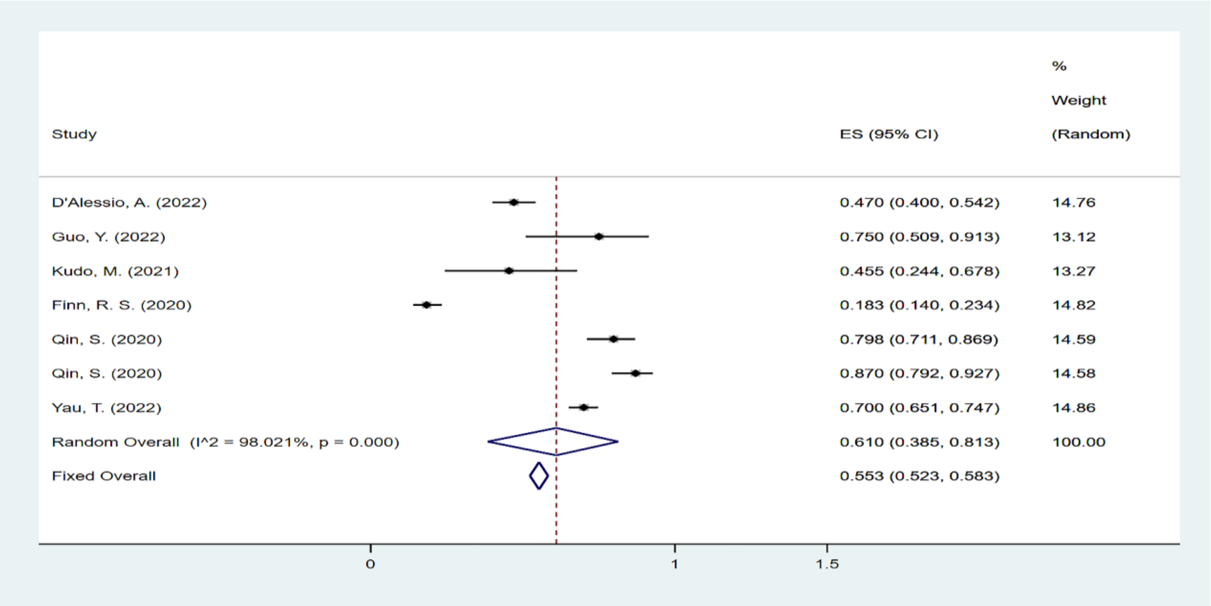

Supplementary Figure S1 Meta-analysis of the incidence of overall any-grade irAEs.

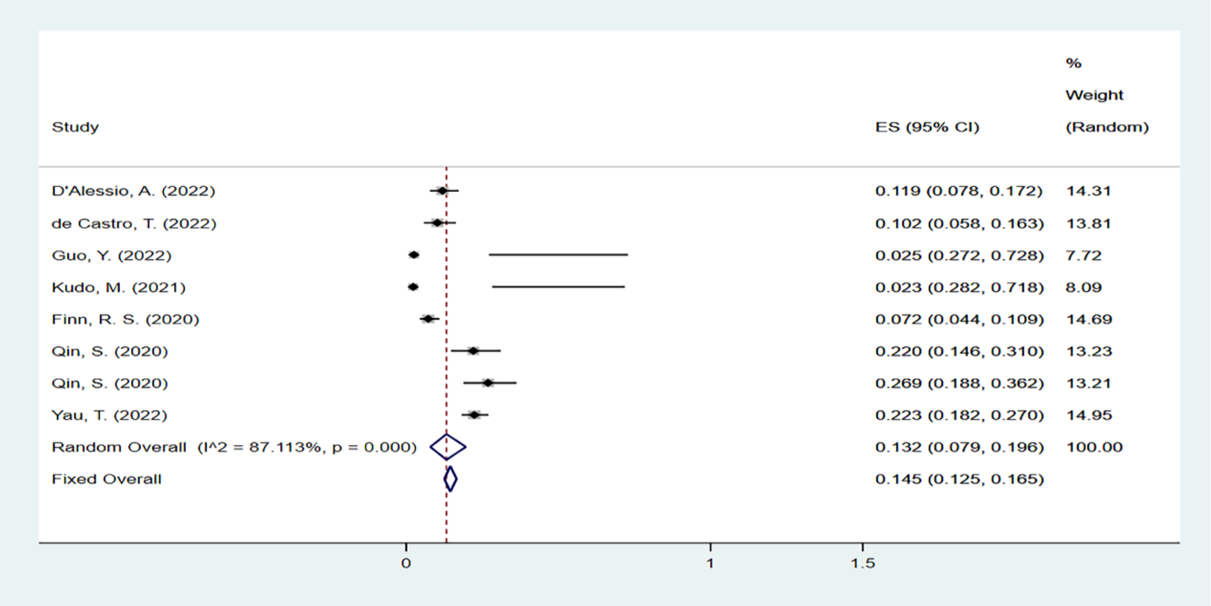

Supplementary Figure S2 Meta-analysis of the incidence of overall grade ≥ 3 irAEs

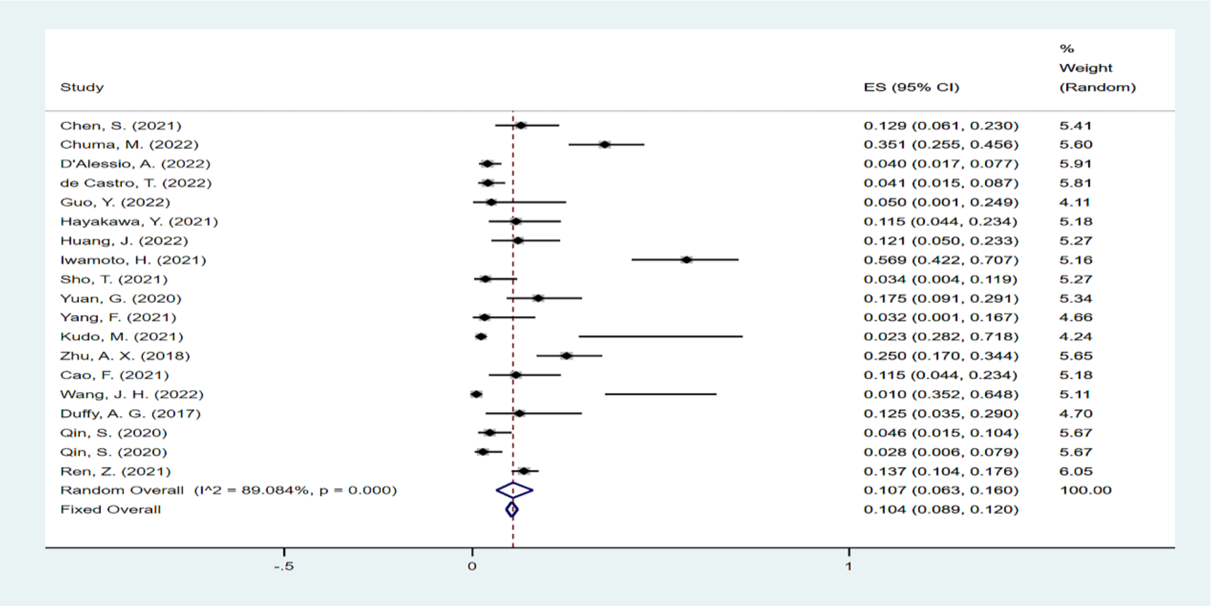

Supplementary Figure S3 Meta-analysis of the incidence of treat-related treatment discontinuation

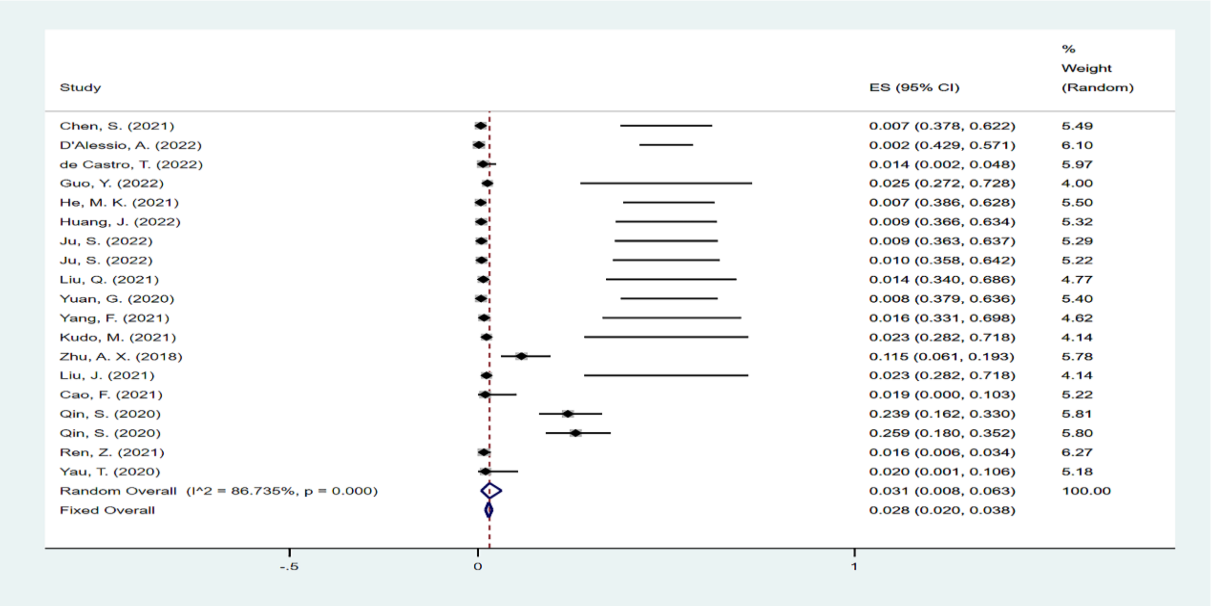

Supplementary Figure S4 Meta-analysis of the incidence of treat-related mortality

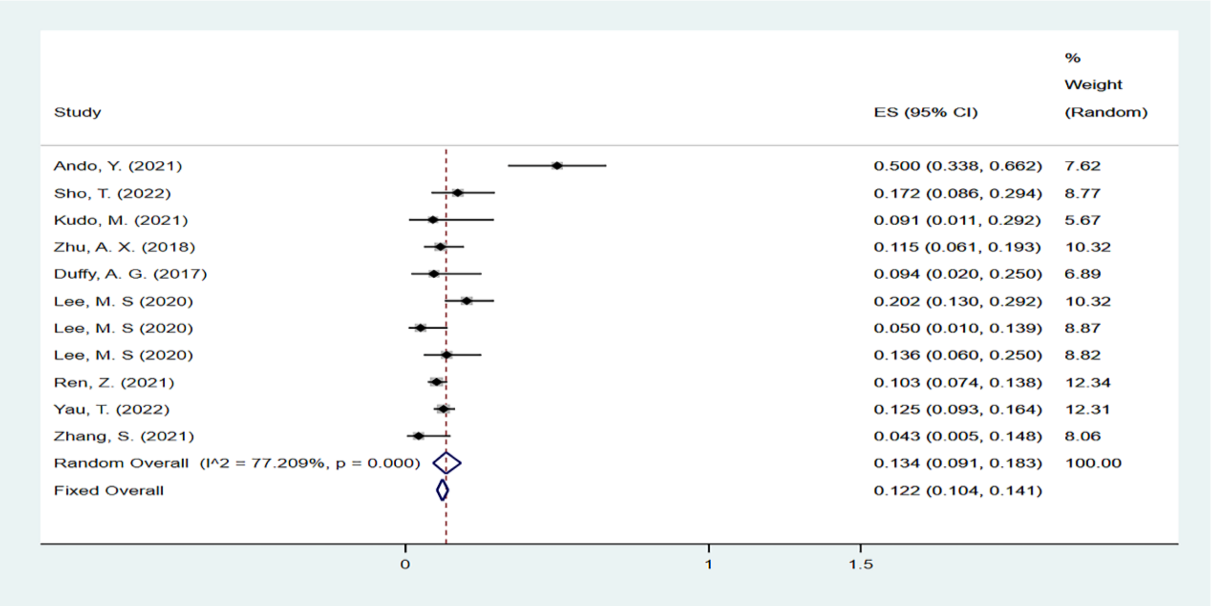

Supplementary Figure S5 Meta-analysis of the incidence of any-grade pruritus

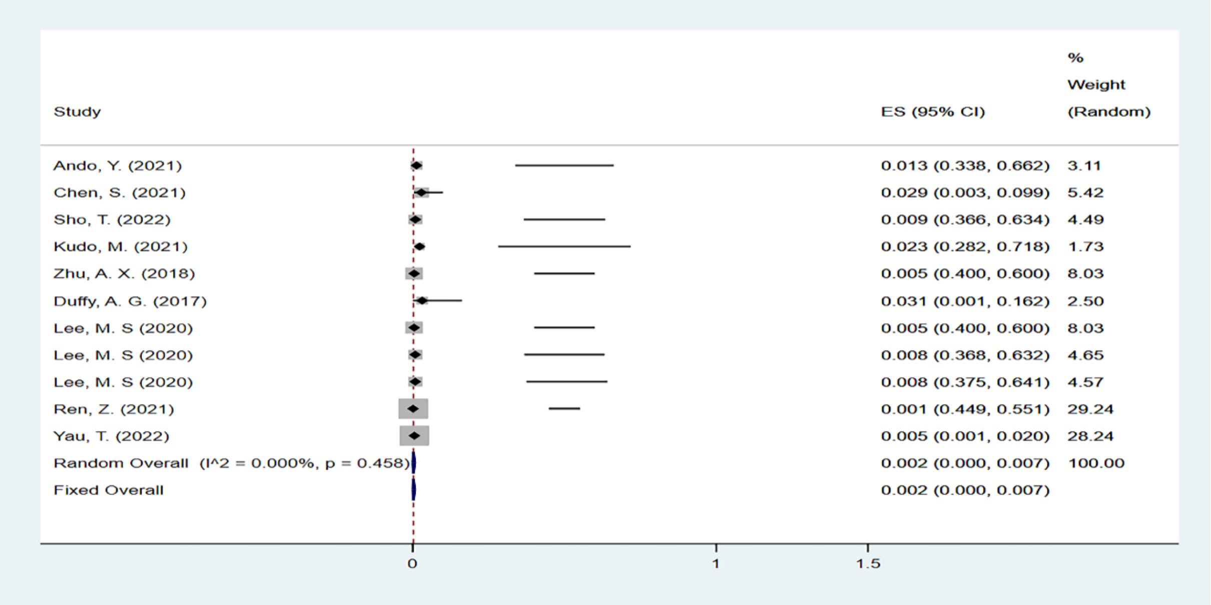

Supplementary Figure S6 Meta-analysis of the incidence of grade ≥ 3 pruritus

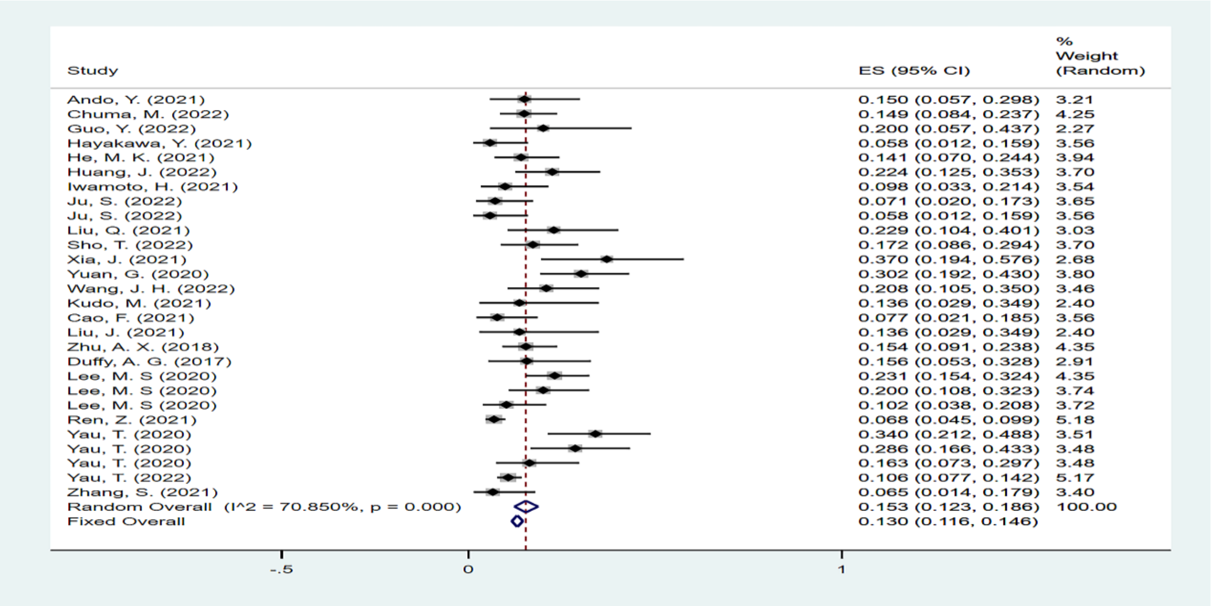

Supplementary Figure S7 Meta-analysis of the incidence of any-grade rash

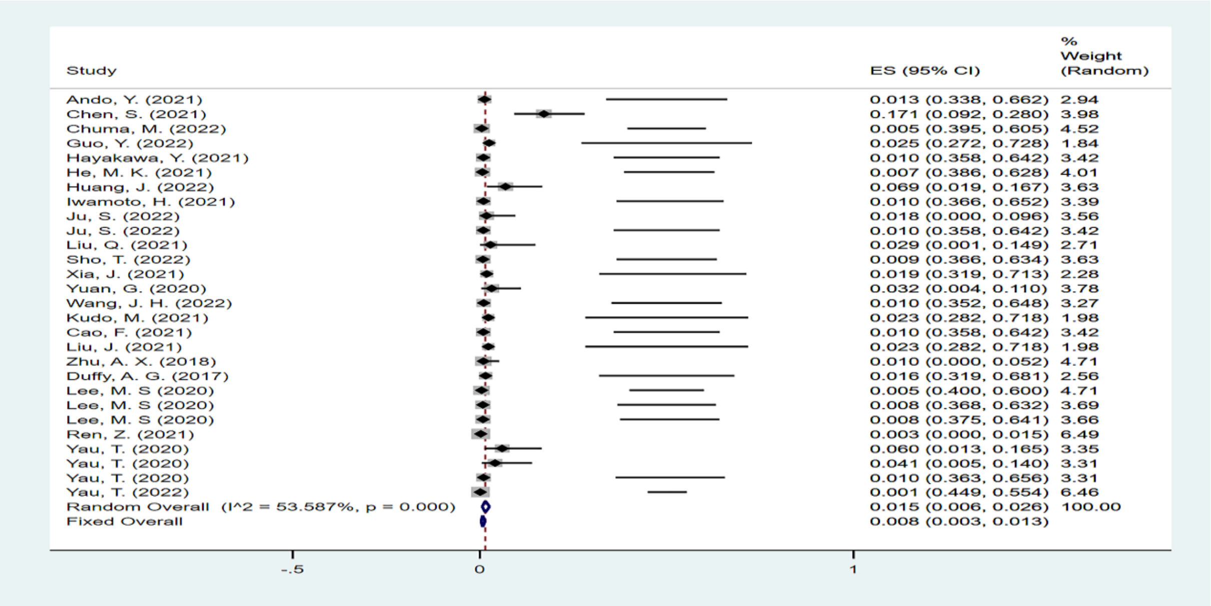

Supplementary Figure S8 Meta-analysis of the incidence of grade ≥ 3 rash

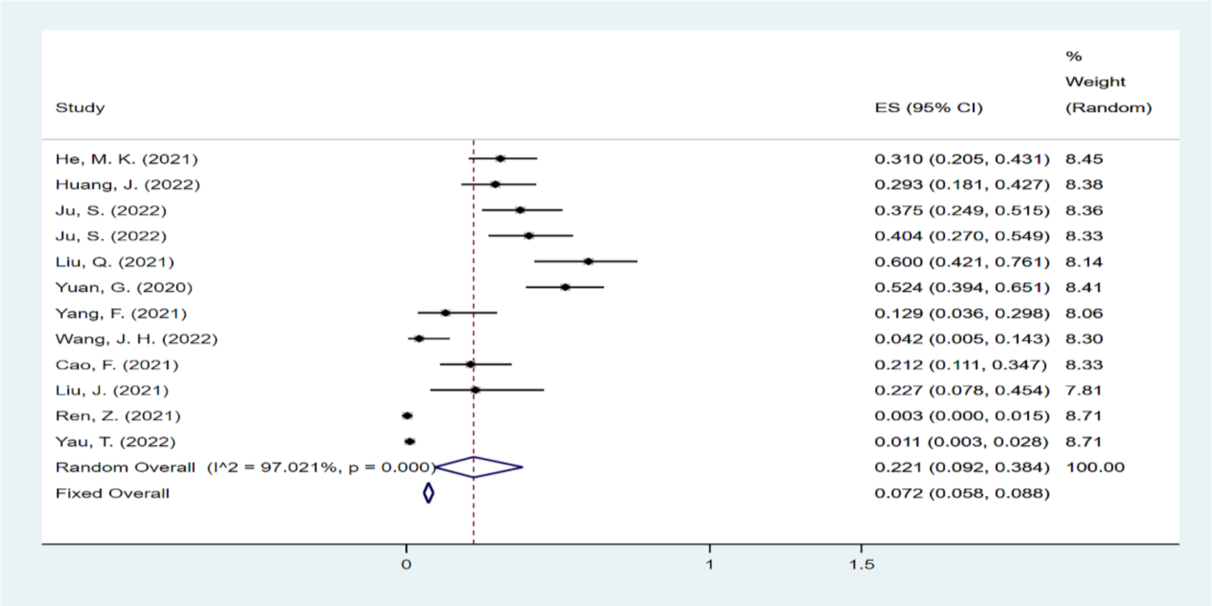

Supplementary Figure S9 Meta-analysis of the incidence of any-grade hand–foot skin reaction

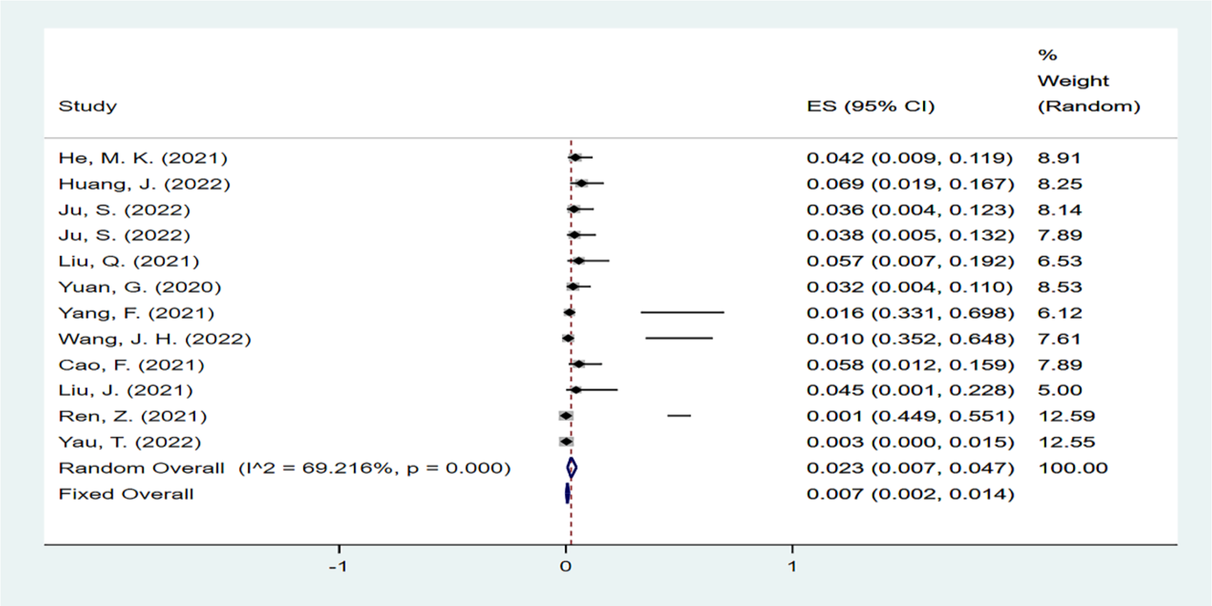

Supplementary Figure S10 Meta-analysis of the incidence of grade ≥ 3 hand–foot skin reaction

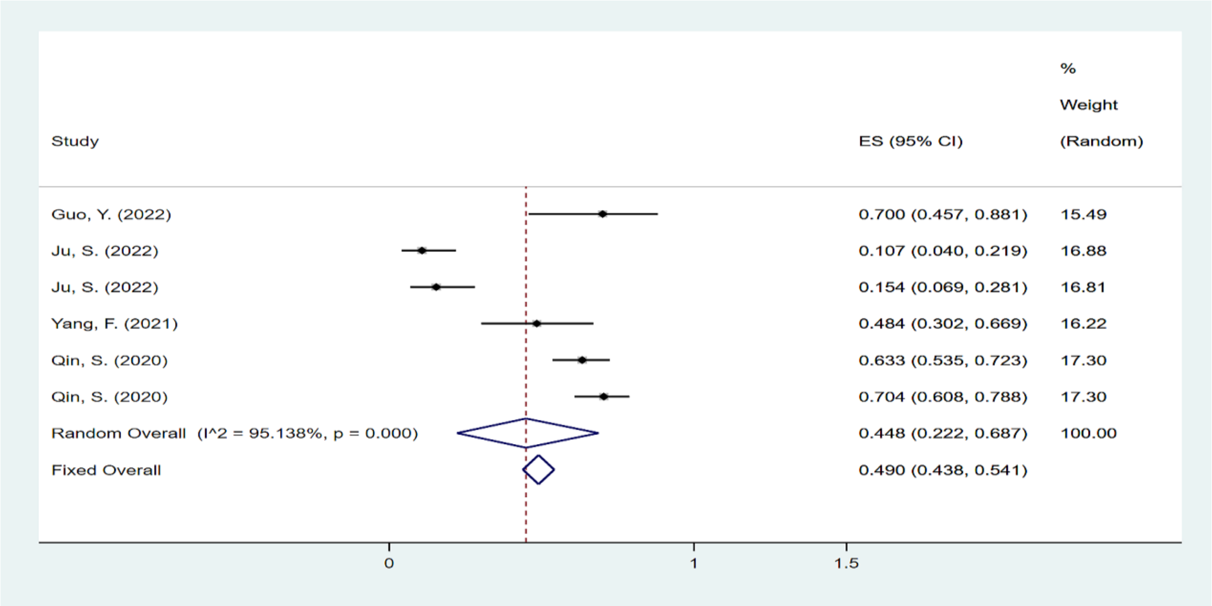

Supplementary Figure S11 Meta-analysis of the incidence of any-grade RCCEP

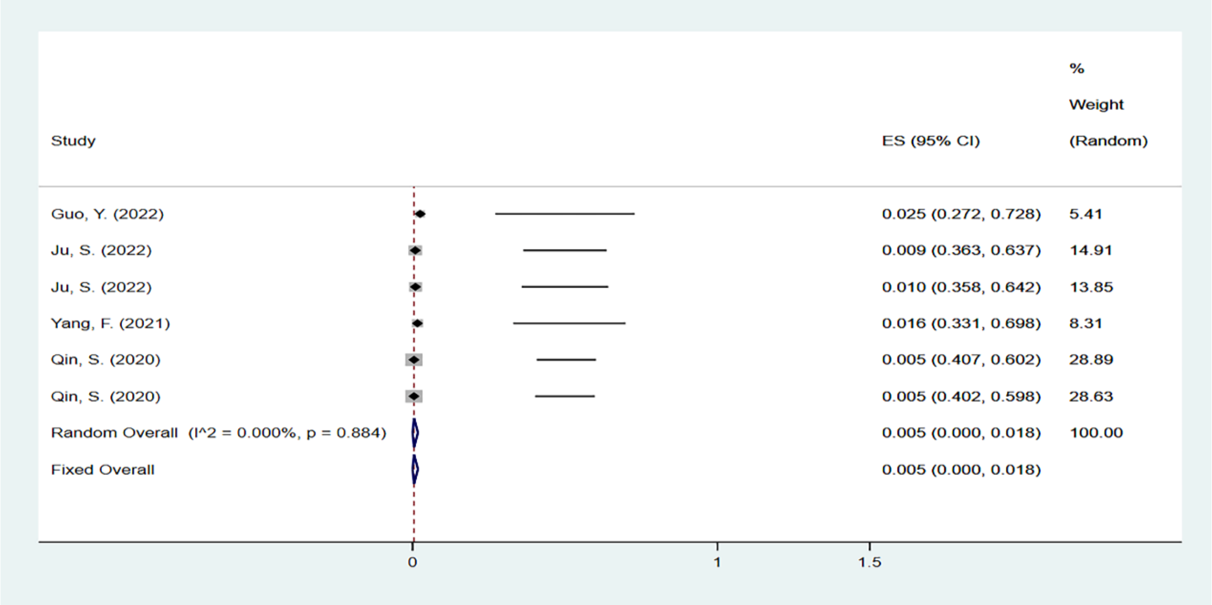

Supplementary Figure S12 Meta-analysis of the incidence of grade ≥ 3 RCCEP

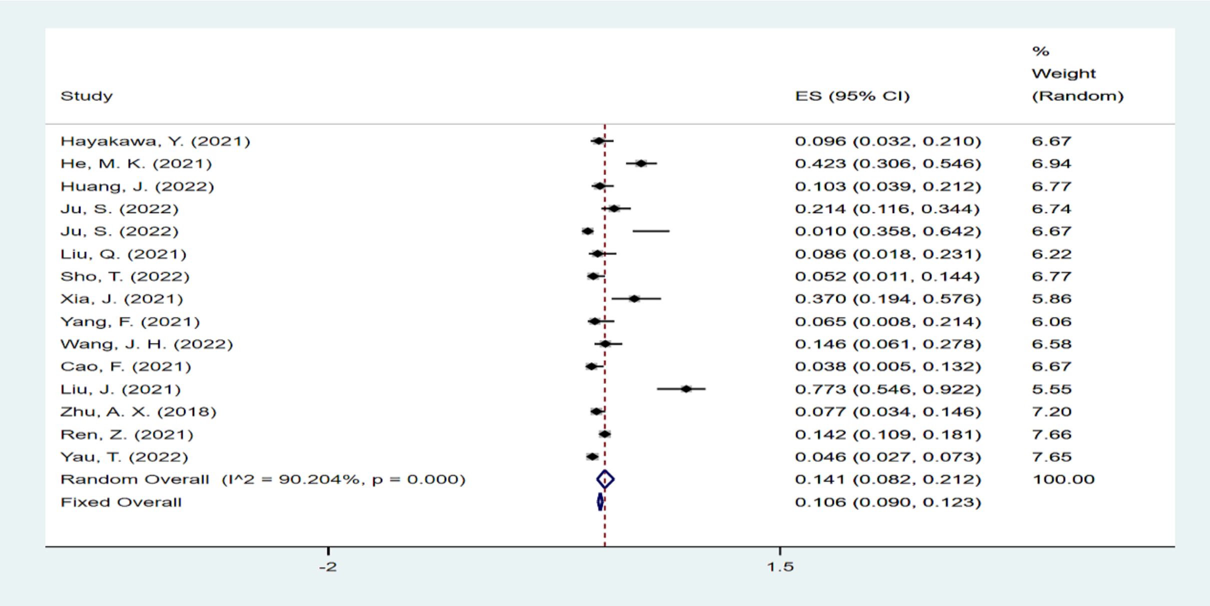

Supplementary Figure S13 Meta-analysis of the incidence of any-grade nausea and vomiting

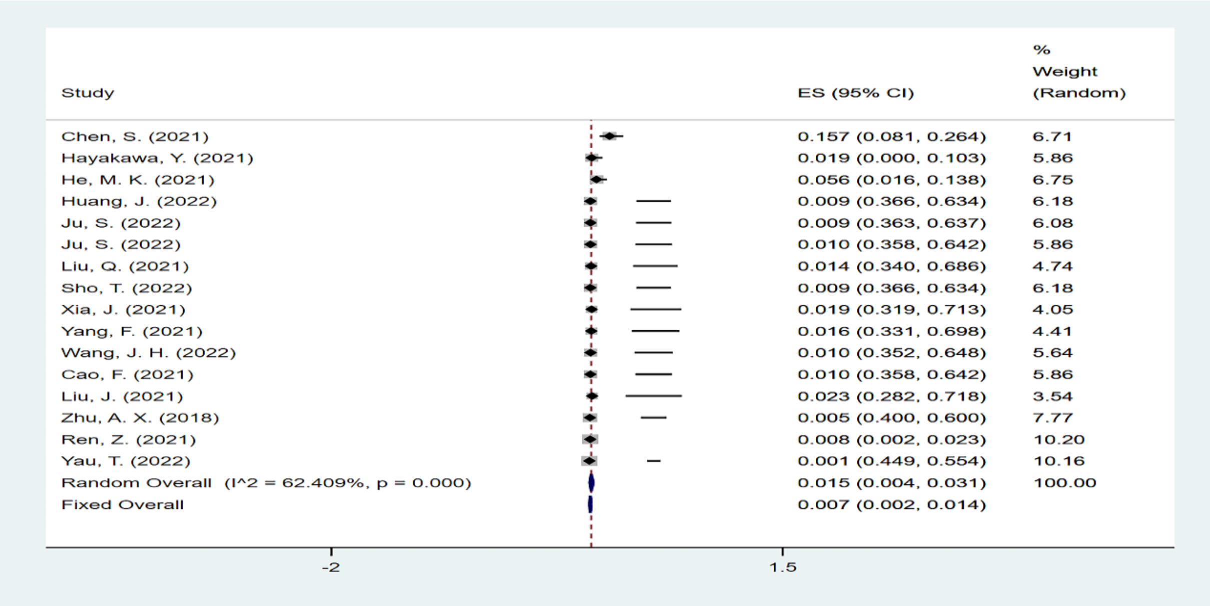

Supplementary Figure S14 Meta-analysis of the incidence of grade ≥ 3 nausea and vomiting

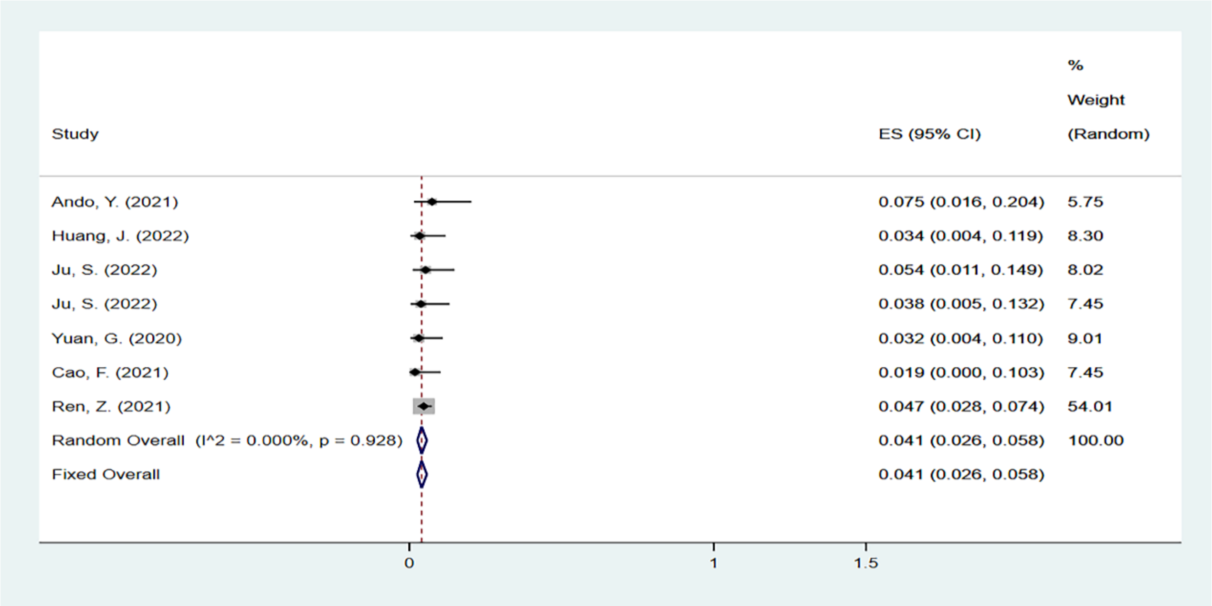

Supplementary Figure S15 Meta-analysis of the incidence of any-grade gastrointestinal bleeding

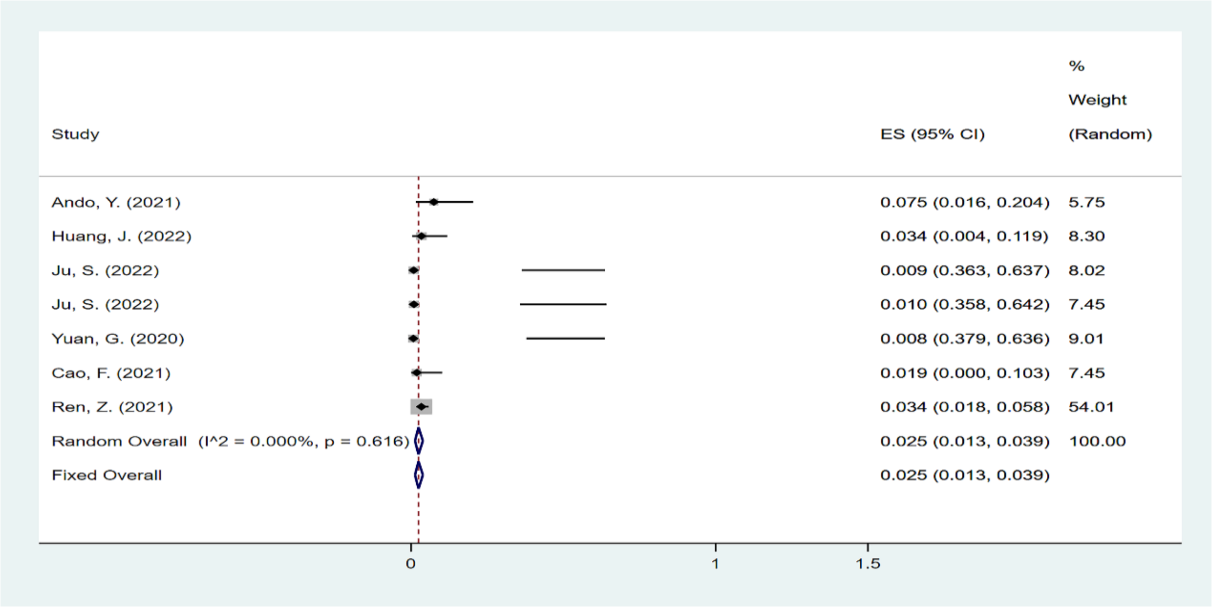

Supplementary Figure S16 Meta-analysis of the incidence of grade ≥ 3 gastrointestinal bleeding

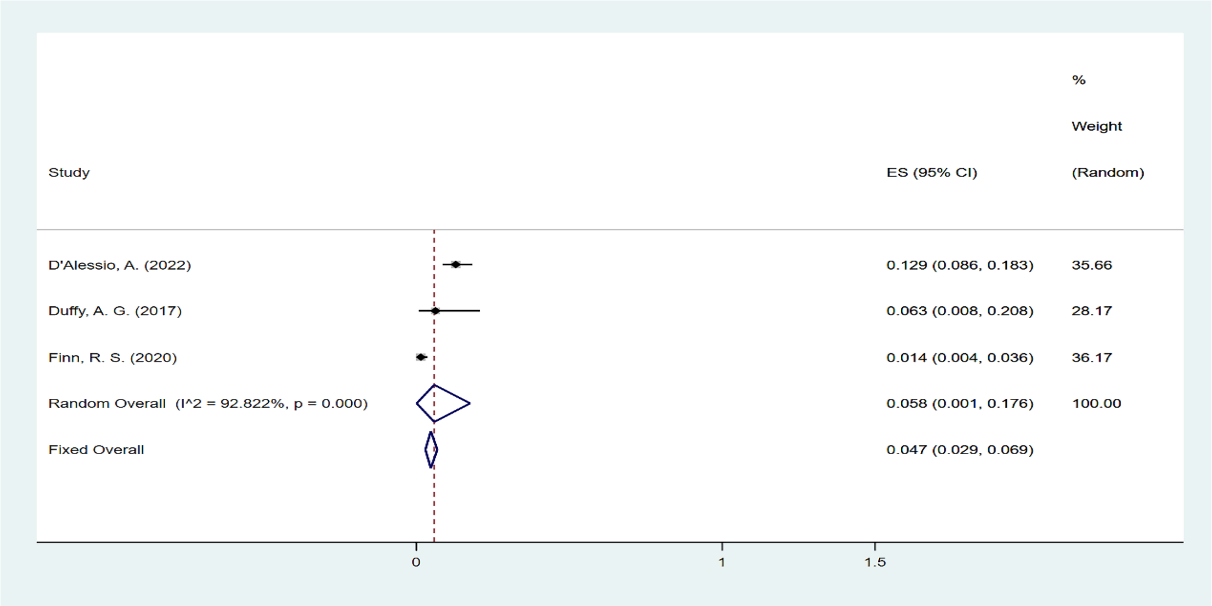

Supplementary Figure S17 Meta-analysis of the incidence of any-grade colitis

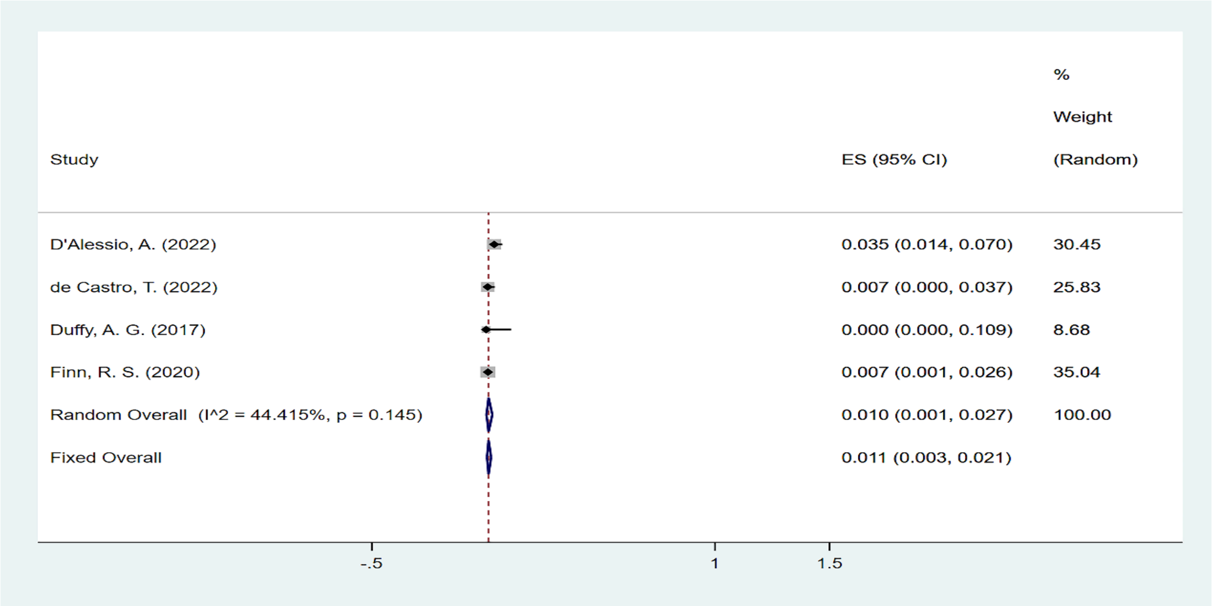

Supplementary Figure S18 Meta-analysis of the incidence of grade ≥ 3 colitis

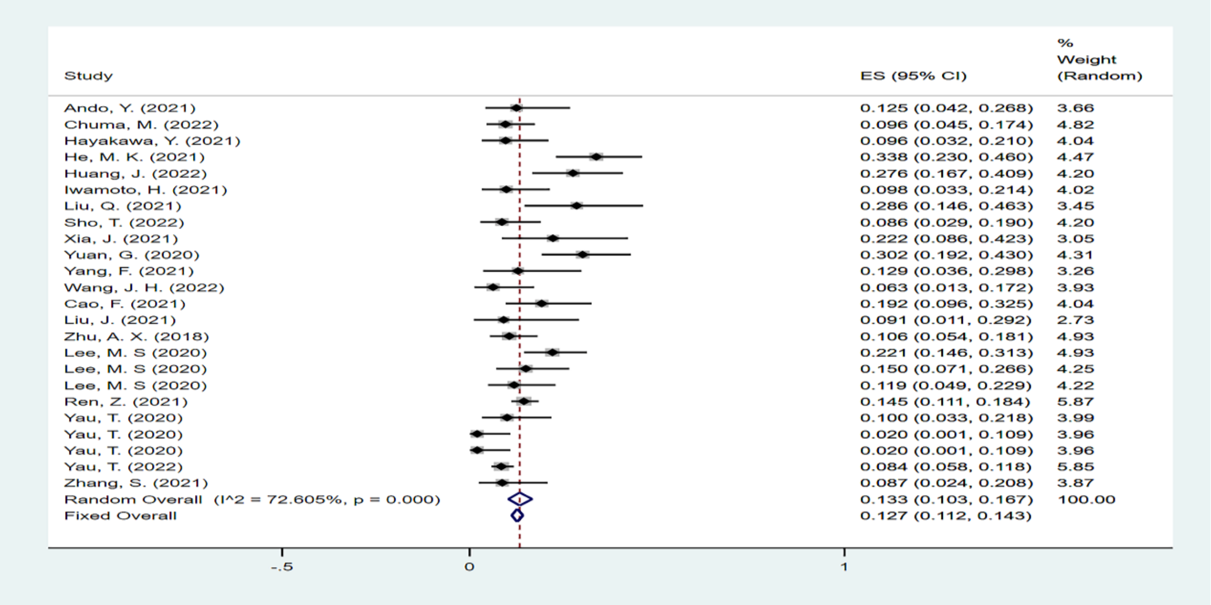

Supplementary Figure S19 Meta-analysis of the incidence of any-grade diarrhea

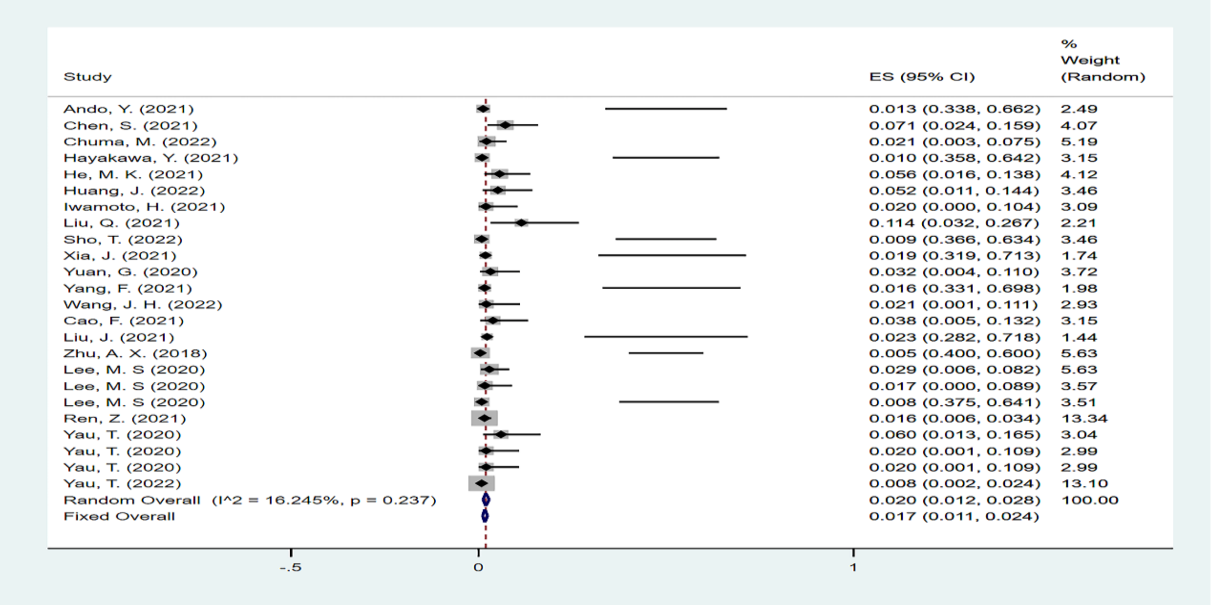

Supplementary Figure S20 Meta-analysis of the incidence of grade ≥ 3 diarrhea

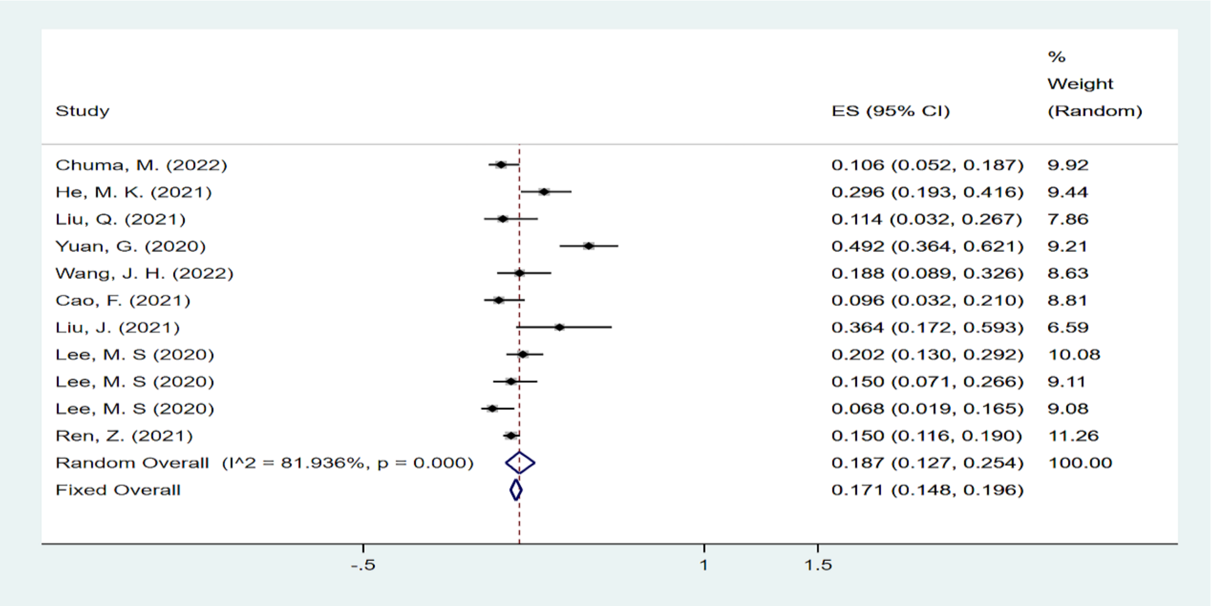

Supplementary Figure S21 Meta-analysis of the incidence of any-grade abdominal pain

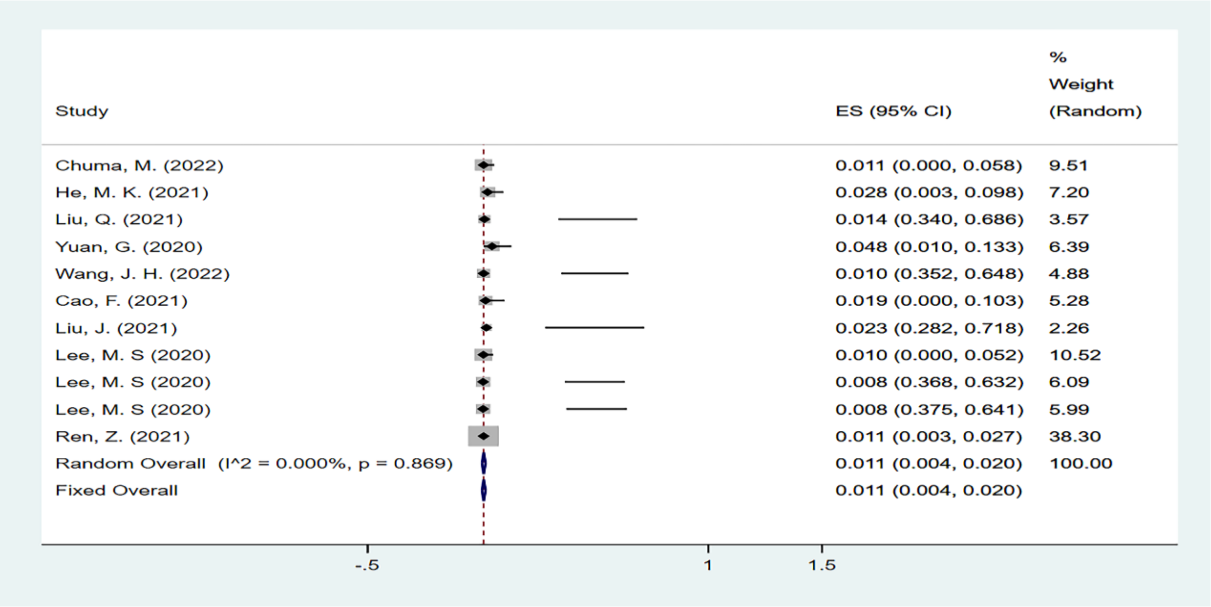

Supplementary Figure S22 Meta-analysis of the incidence of grade ≥ 3 abdominal pain

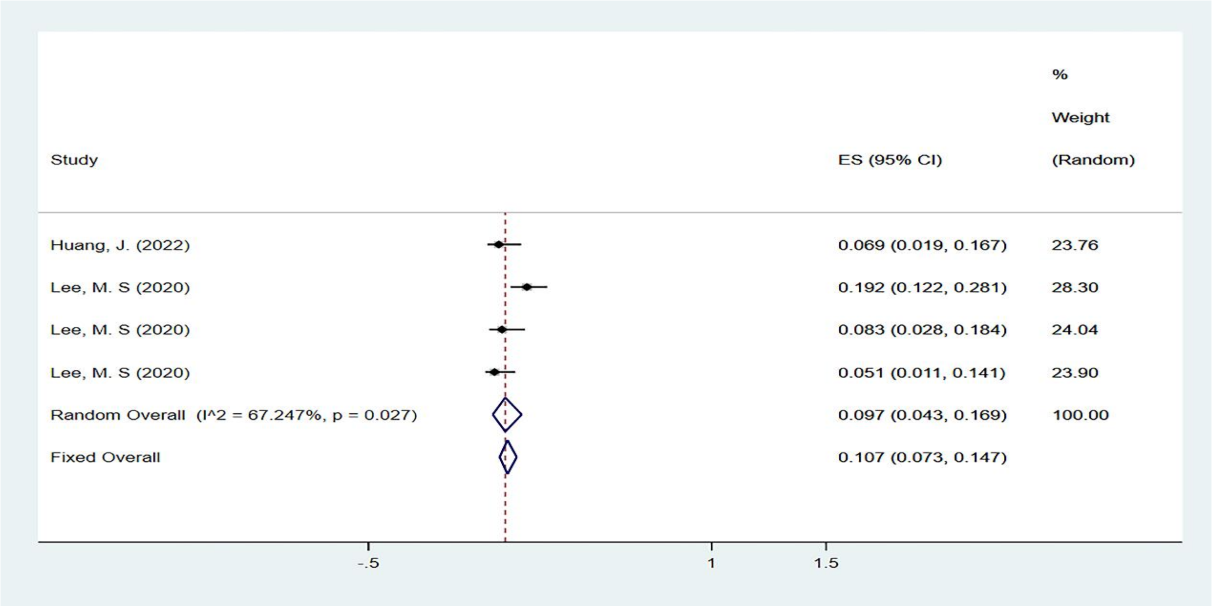

Supplementary Figure S23 Meta-analysis of the incidence of any-grade constipation

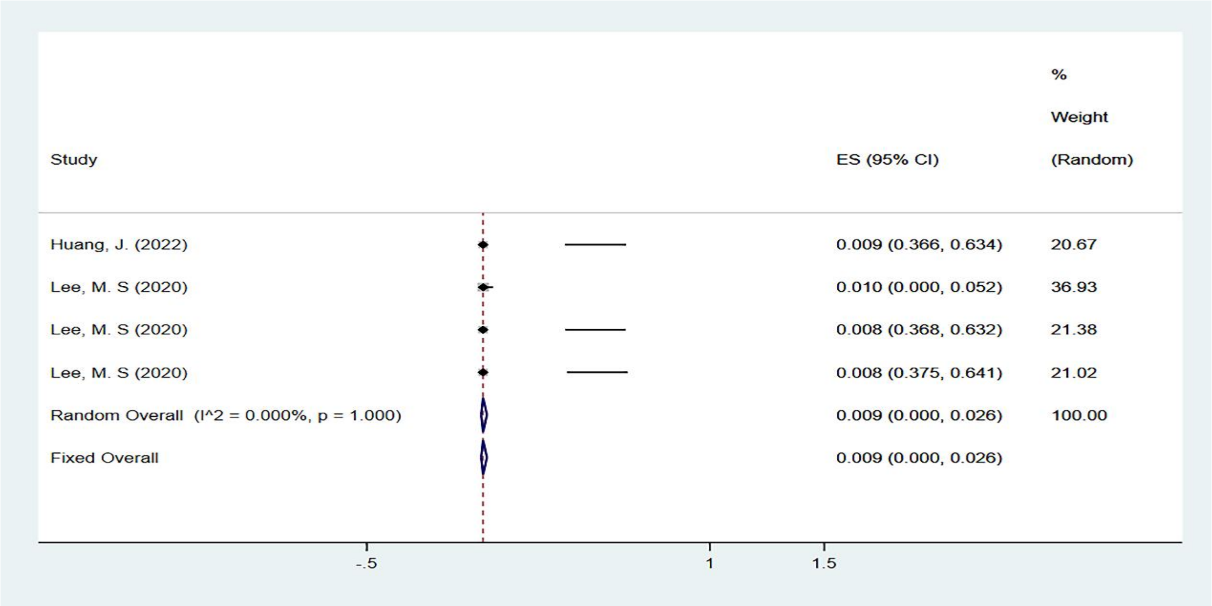

Supplementary Figure S24 Meta-analysis of the incidence of grade ≥ 3 constipation

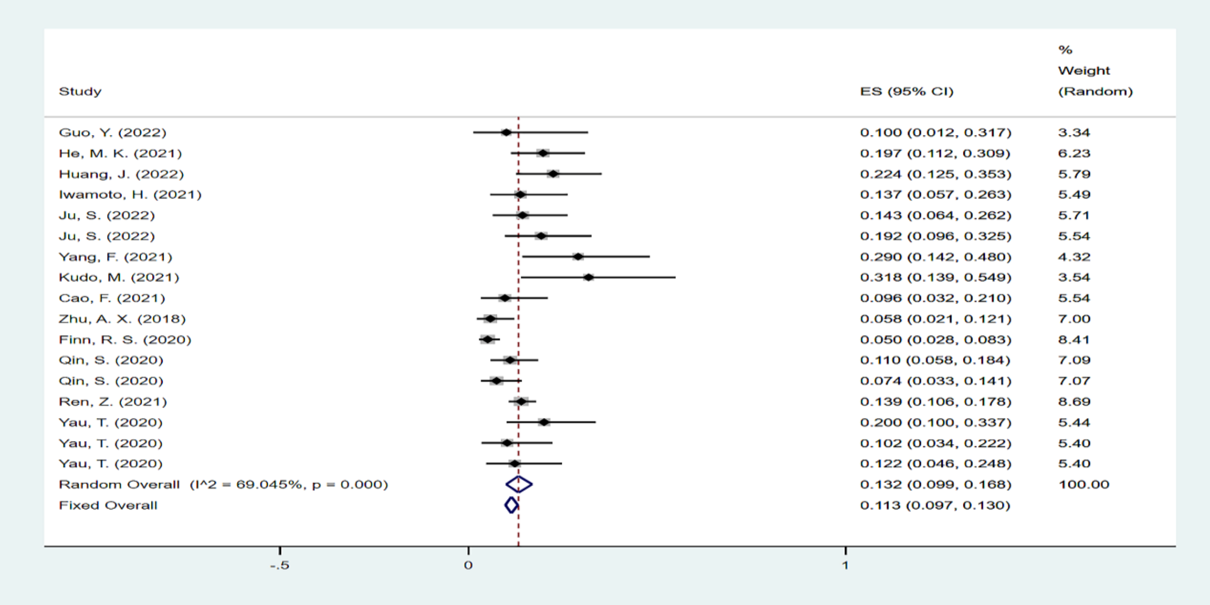

Supplementary Figure S25 Meta-analysis of the incidence of any-grade hypothyroidism

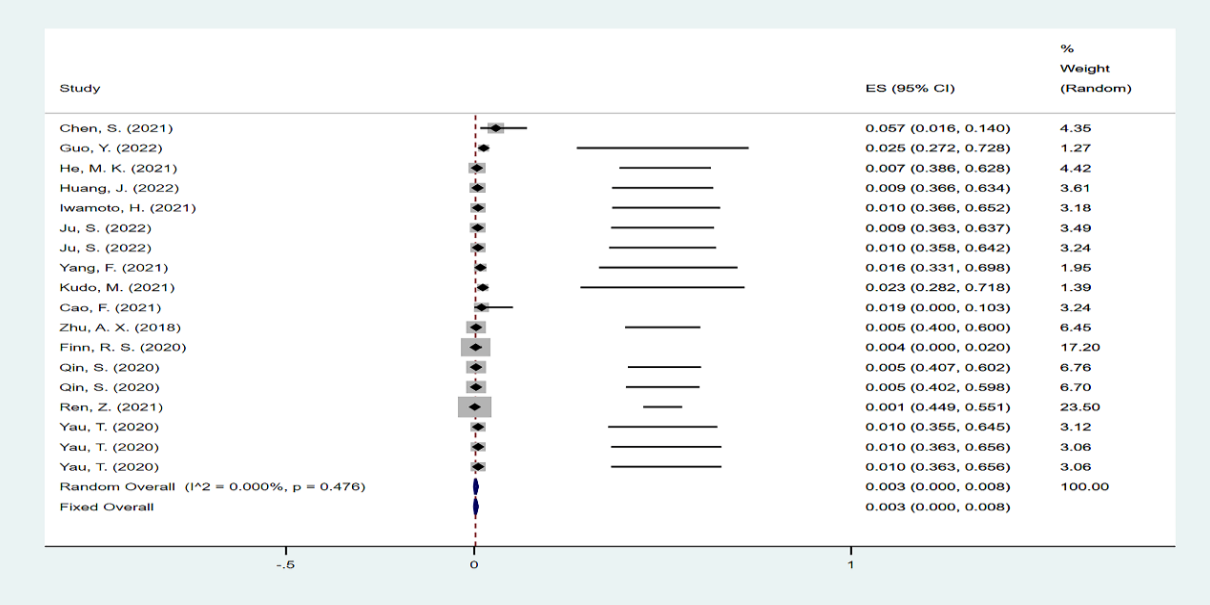

Supplementary Figure S26 Meta-analysis of the incidence of grade ≥ 3 hypothyroidism

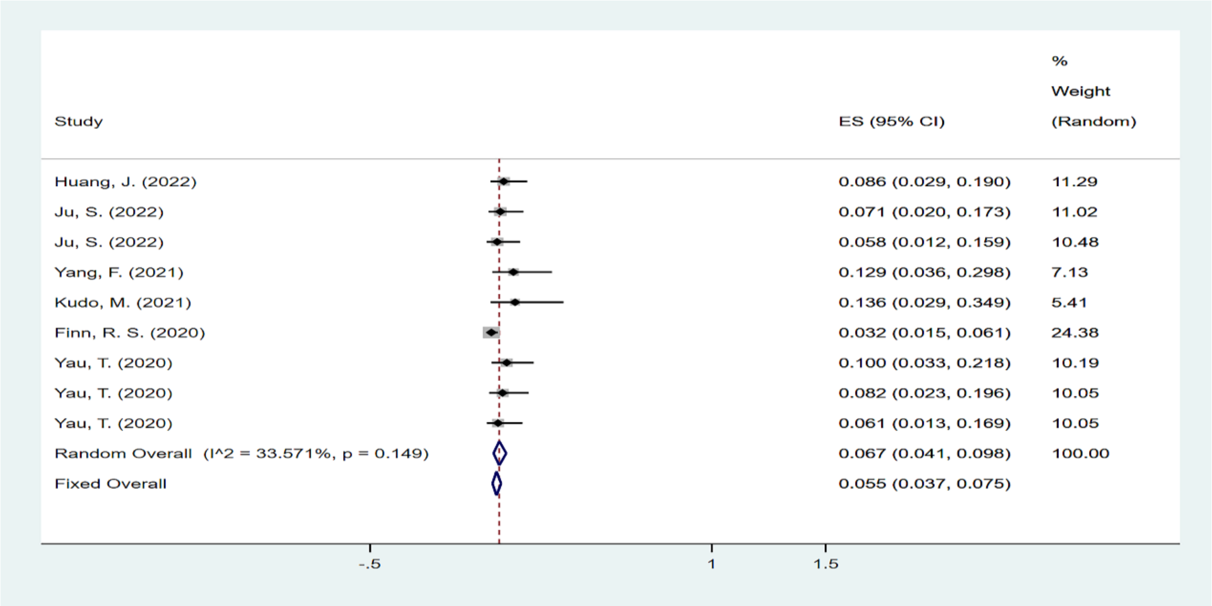

Supplementary Figure S27 Meta-analysis of the incidence of any-grade hyperthyroidism

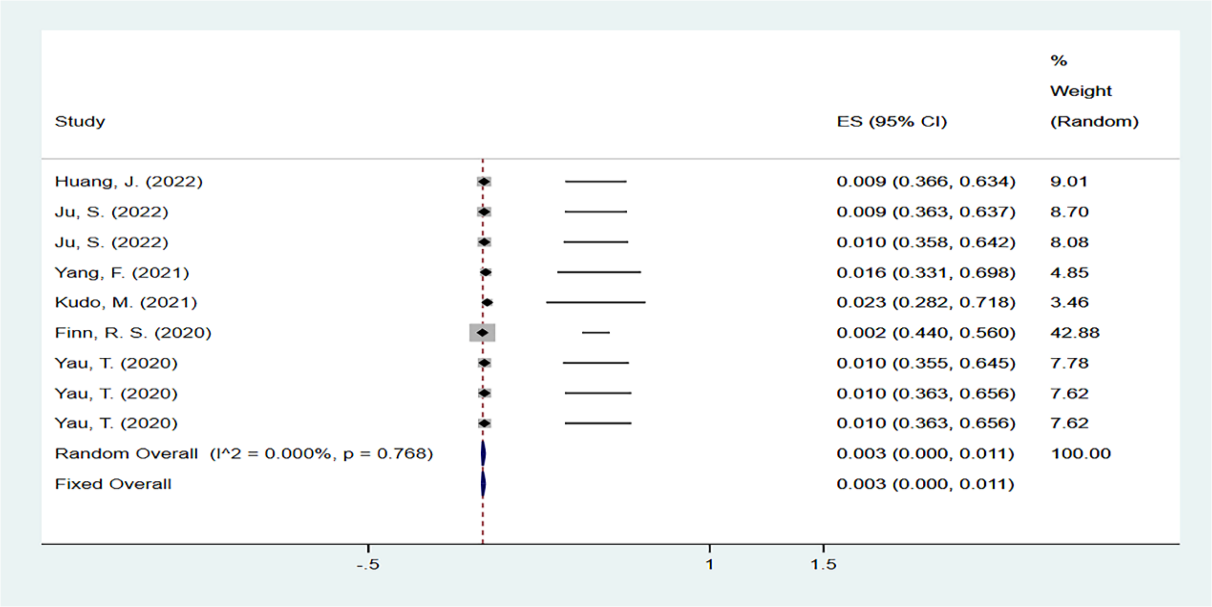

Supplementary Figure S28 Meta-analysis of the incidence of grade ≥ 3 hyperthyroidism

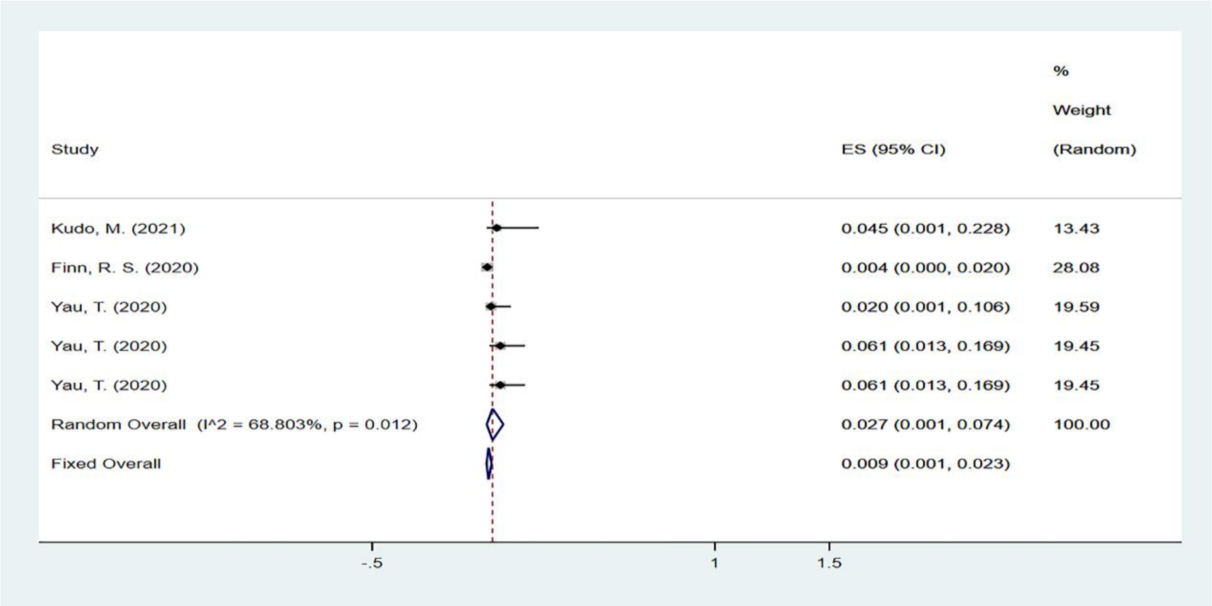

Supplementary Figure S29 Meta-analysis of the incidence of any-grade thyroiditis chronic

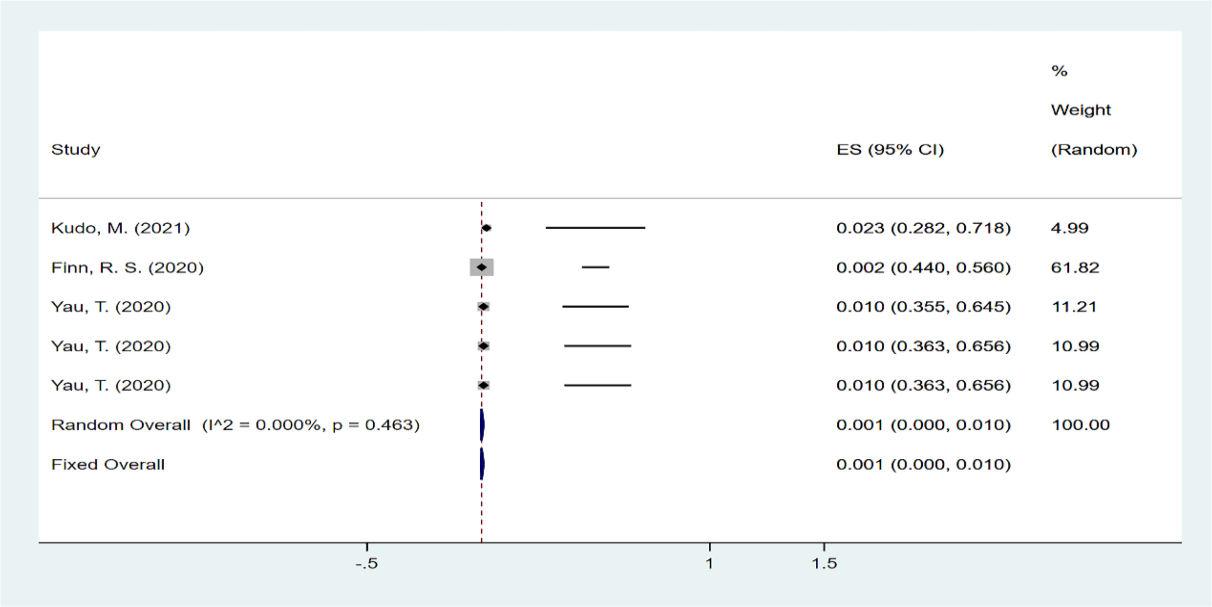

Supplementary Figure S30 Meta-analysis of the incidence of grade ≥ 3 thyroiditis chronic

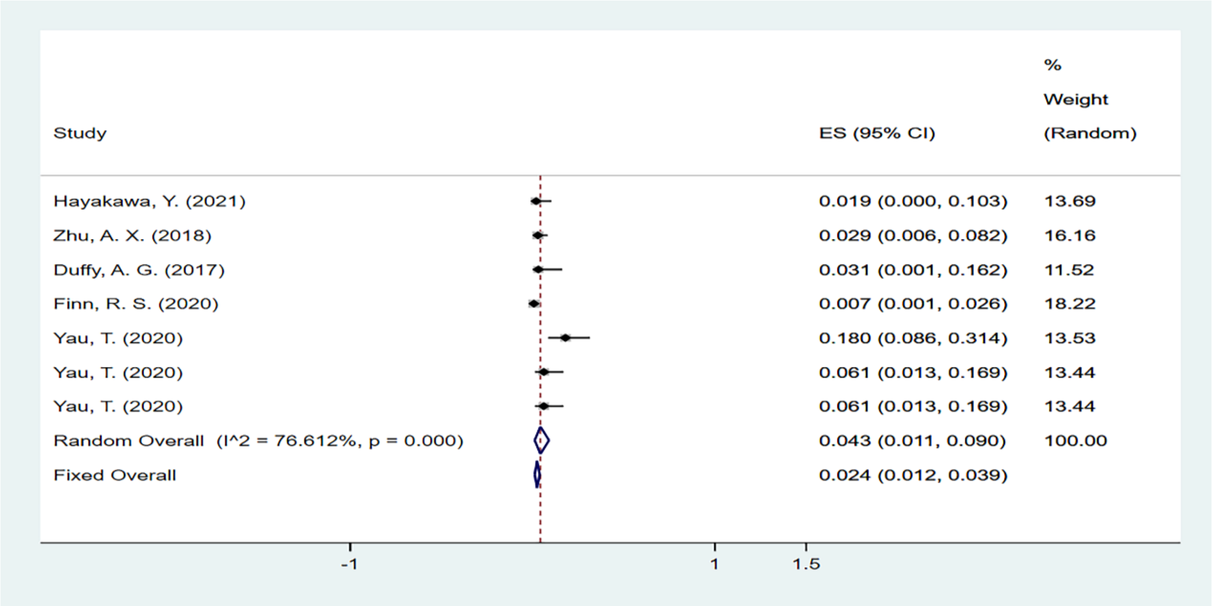

Supplementary Figure S31 Meta-analysis of the incidence of any-grade adrenal insufficiency

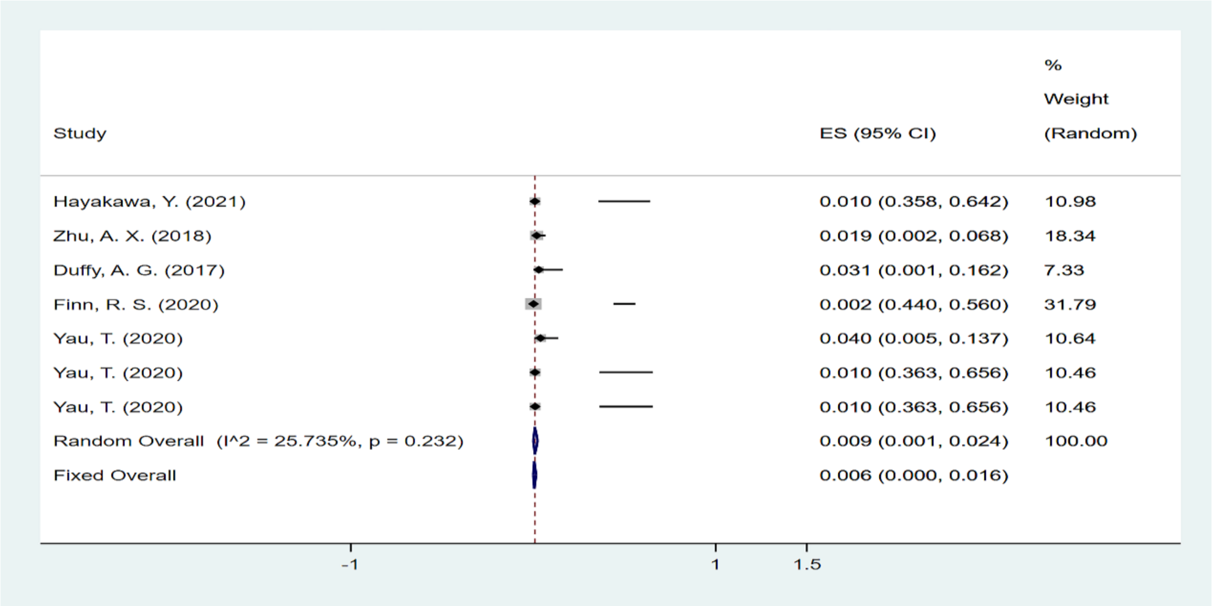

Supplementary Figure S32 Meta-analysis of the incidence of grade ≥ 3 adrenal insufficiency

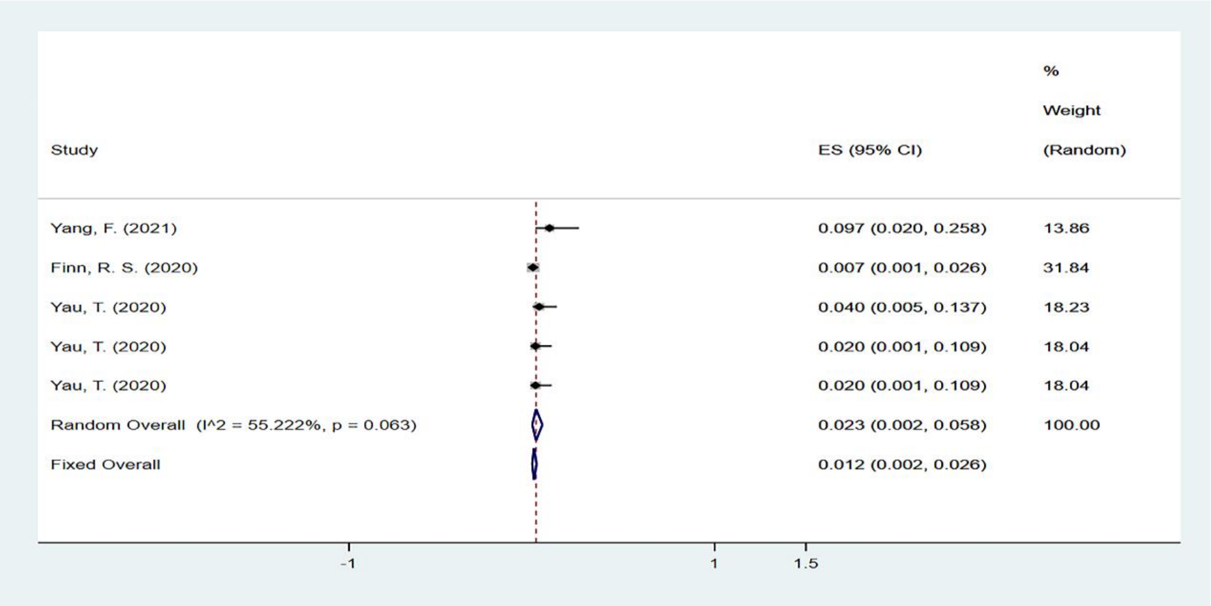

Supplementary Figure S33 Meta-analysis of the incidence of any-grade hypophysitis

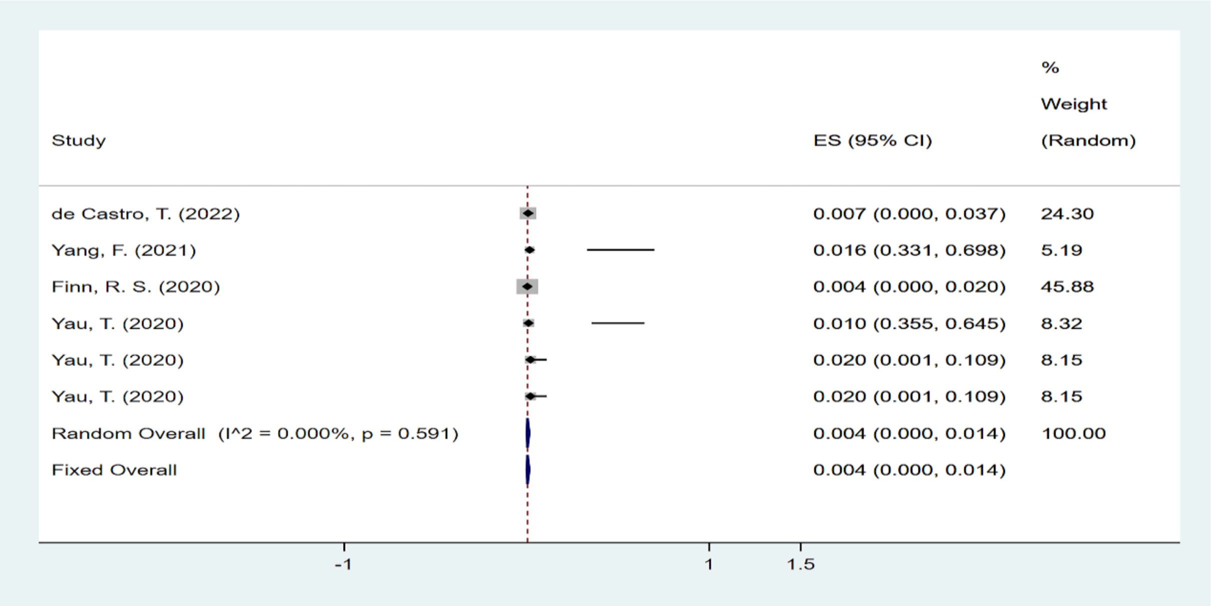

Supplementary Figure S34 Meta-analysis of the incidence of grade ≥ 3 hypophysitis

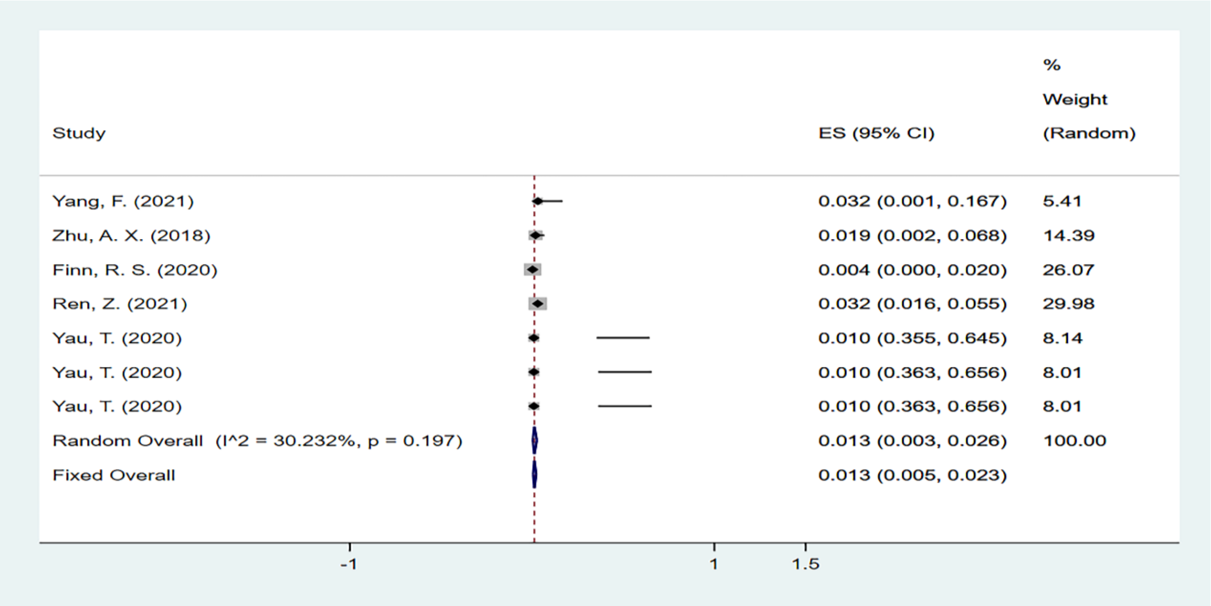

Supplementary Figure S35 Meta-analysis of the incidence of any-grade hyperglycemia

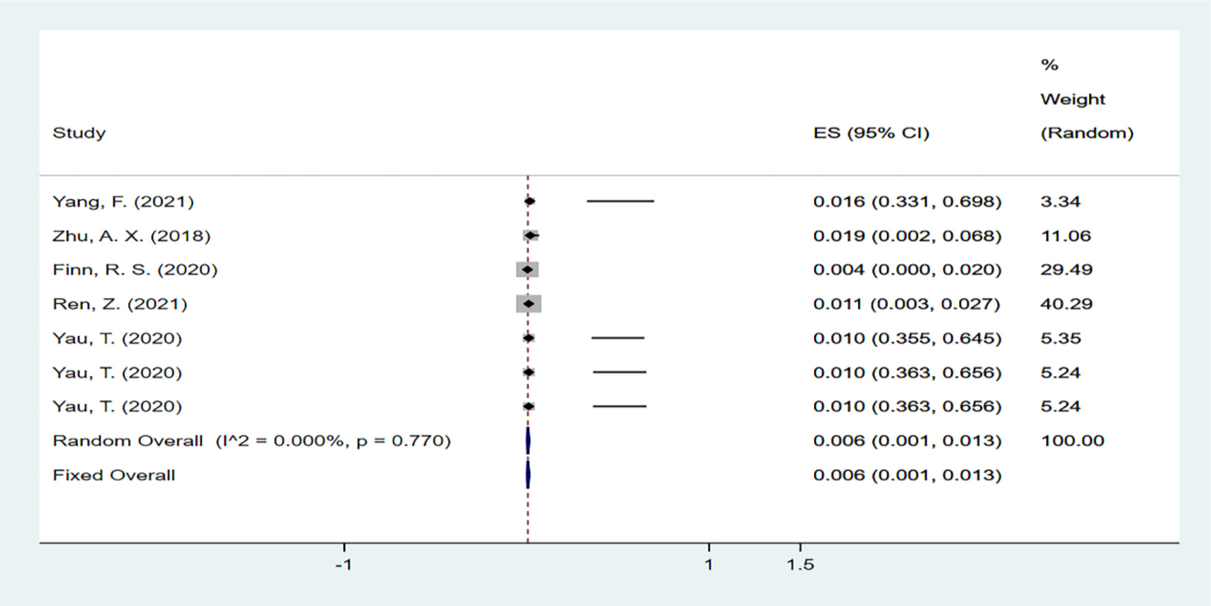

Supplementary Figure S36 Meta-analysis of the incidence of grade ≥ 3 hyperglycemia

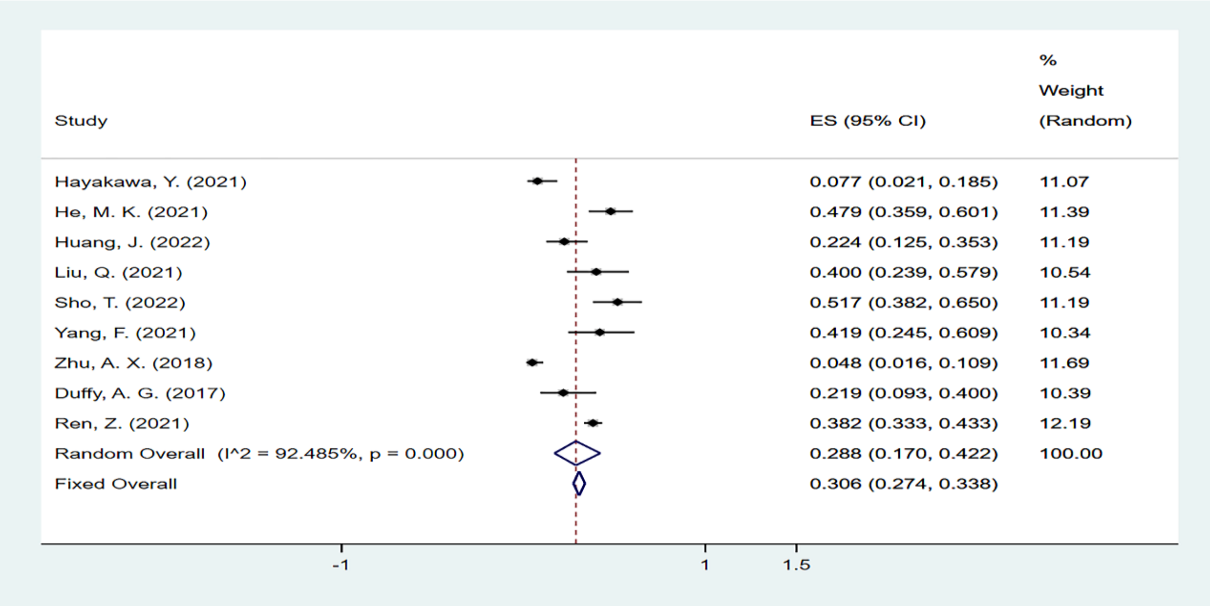

Supplementary Figure S37 Meta-analysis of the incidence of any-grade hyperbilirubinemia

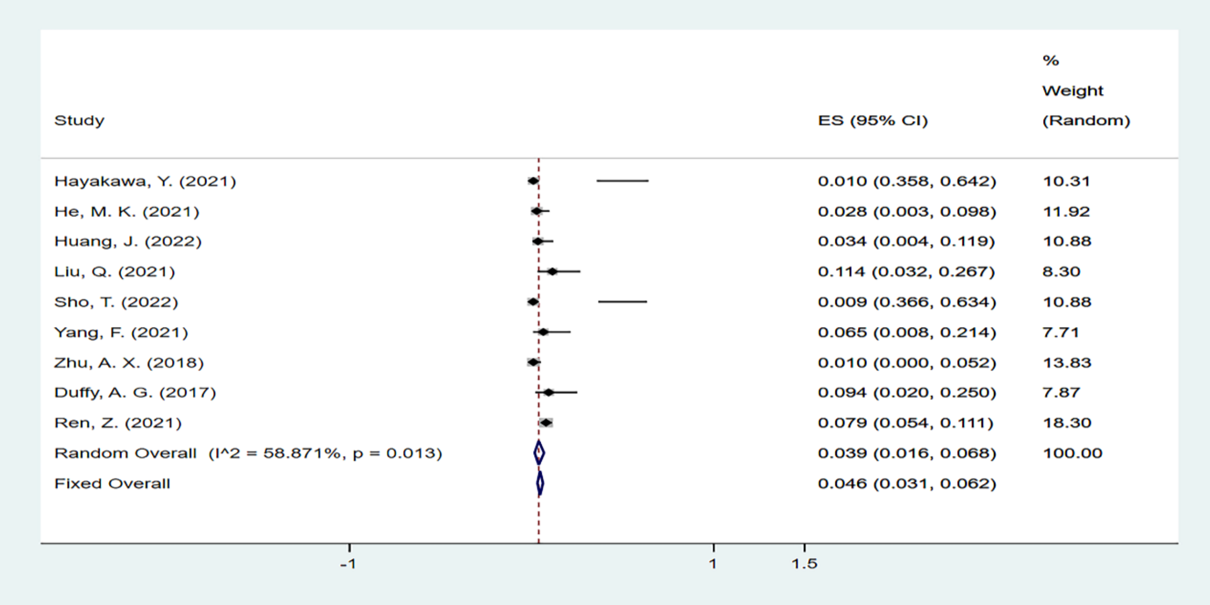

Supplementary Figure S38 Meta-analysis of the incidence of grade ≥ 3 hyperbilirubinemia

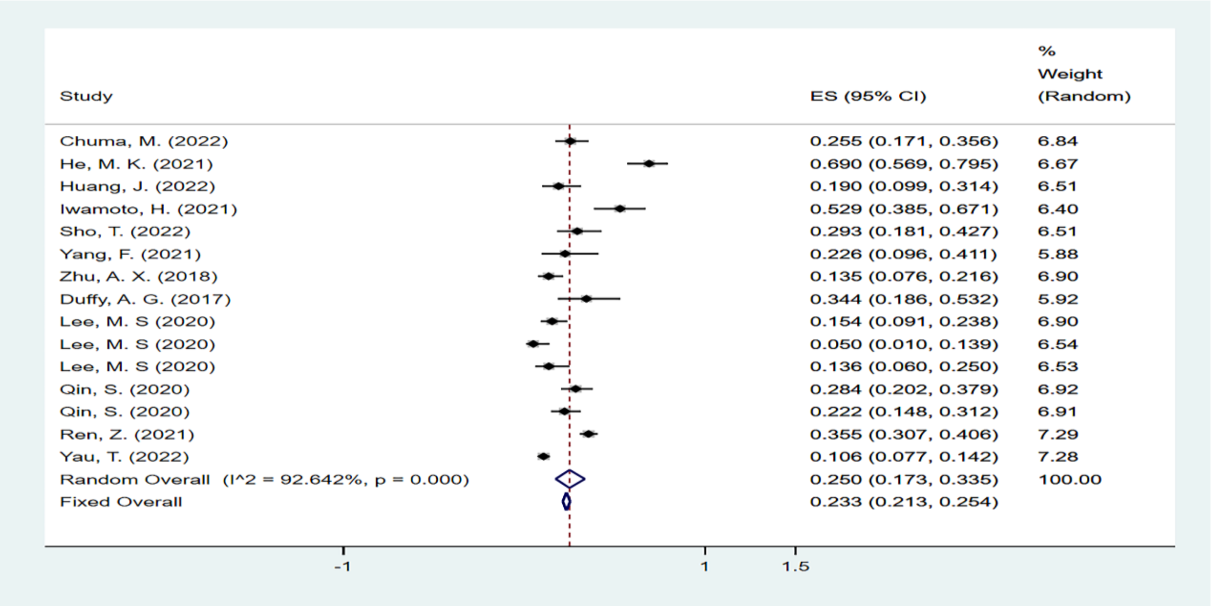

Supplementary Figure S39 Meta-analysis of the incidence of any-grade elevated AST

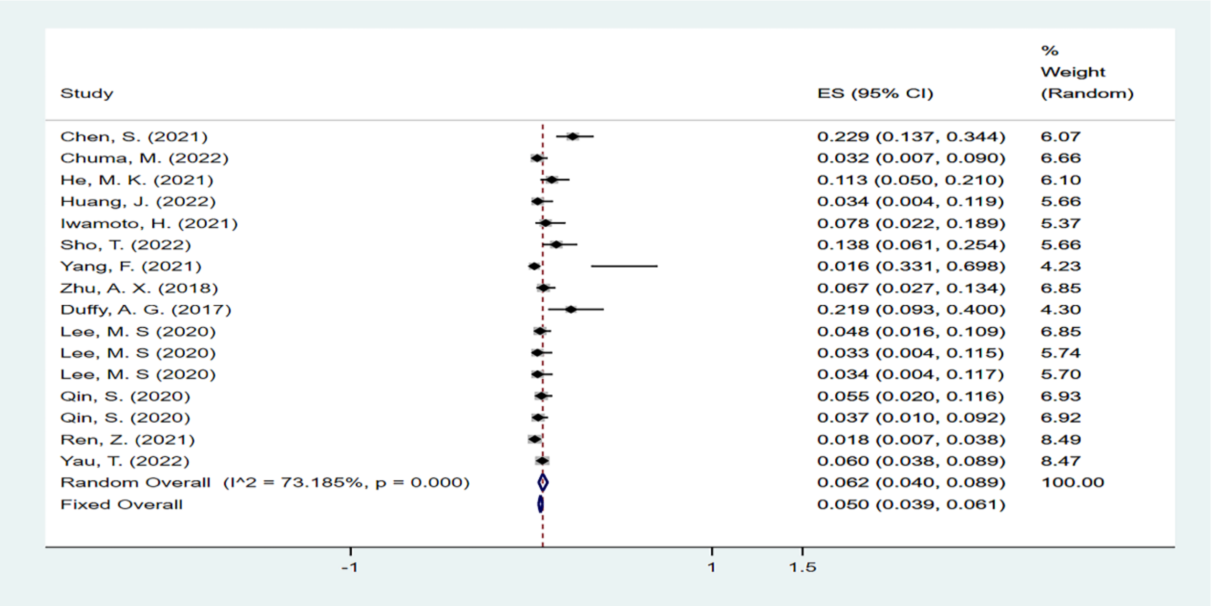

Supplementary Figure S40 Meta-analysis of the incidence of grade ≥ 3 elevated AST

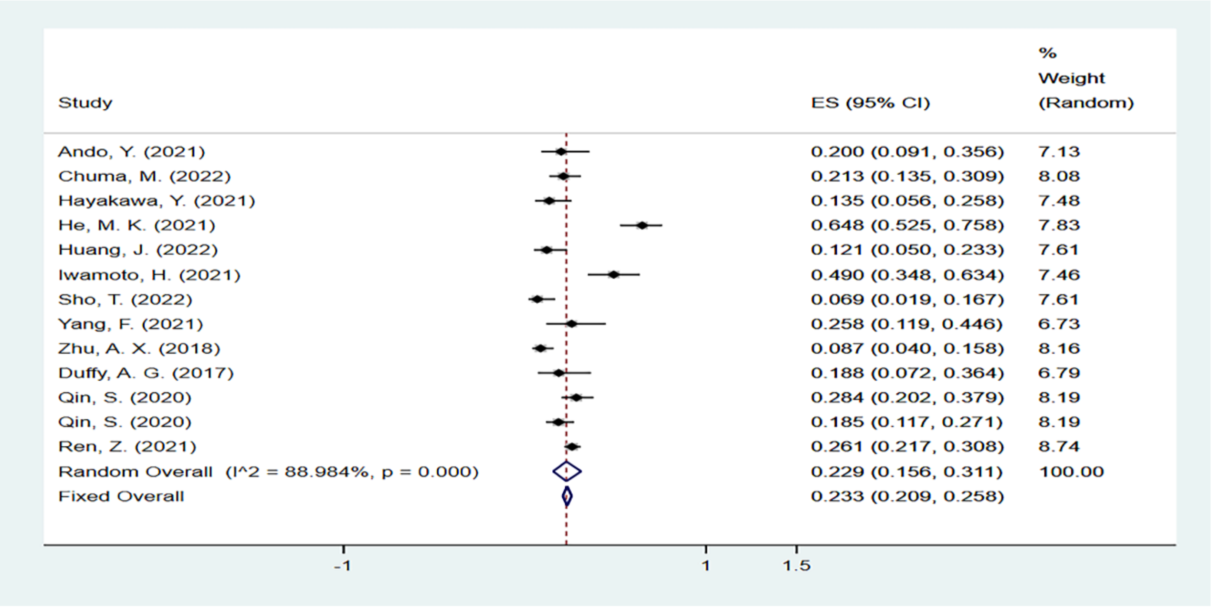

Supplementary Figure S41 Meta-analysis of the incidence of any-grade elevated ALT

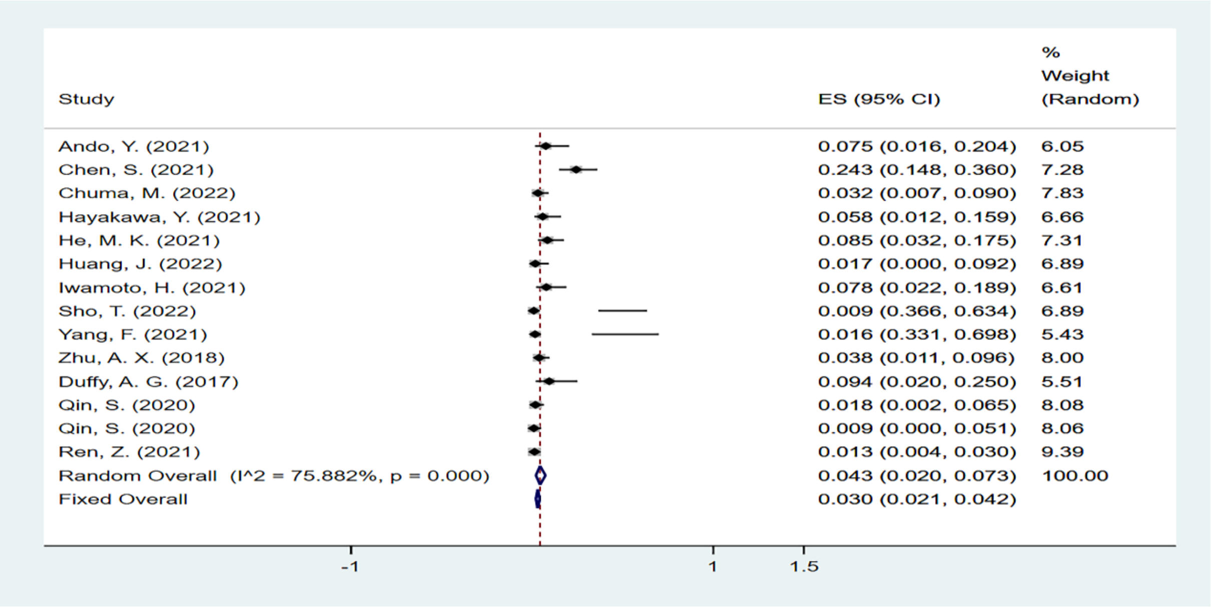

Supplementary Figure S42 Meta-analysis of the incidence of grade ≥ 3 elevated ALT

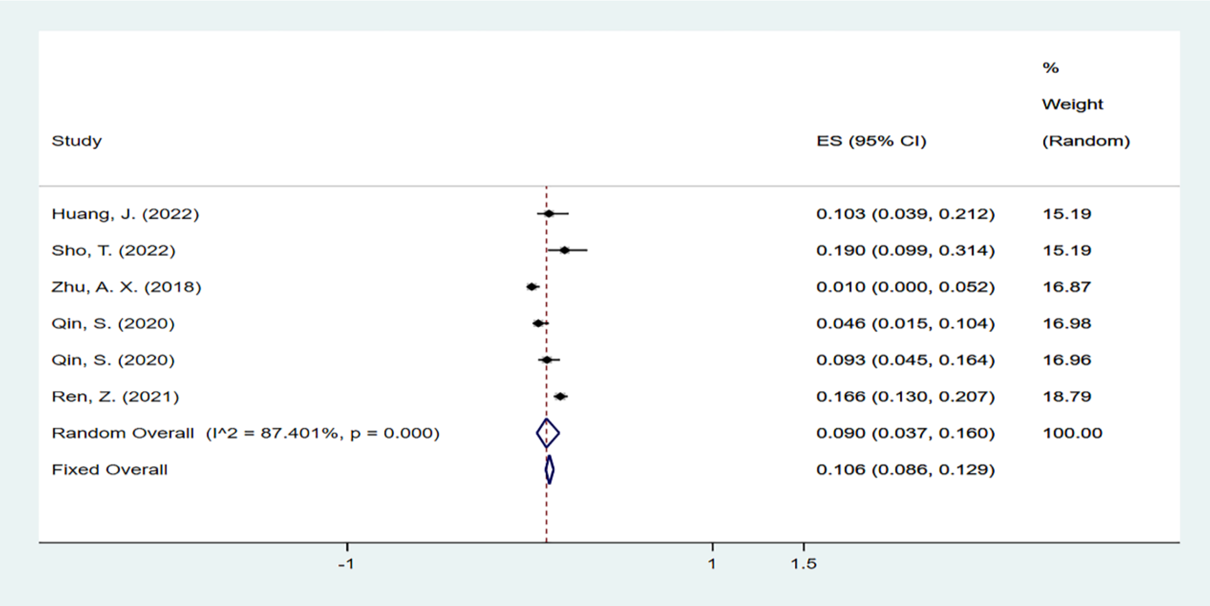

Supplementary Figure S43 Meta-analysis of the incidence of any-grade increased γ-glutamyltransferase

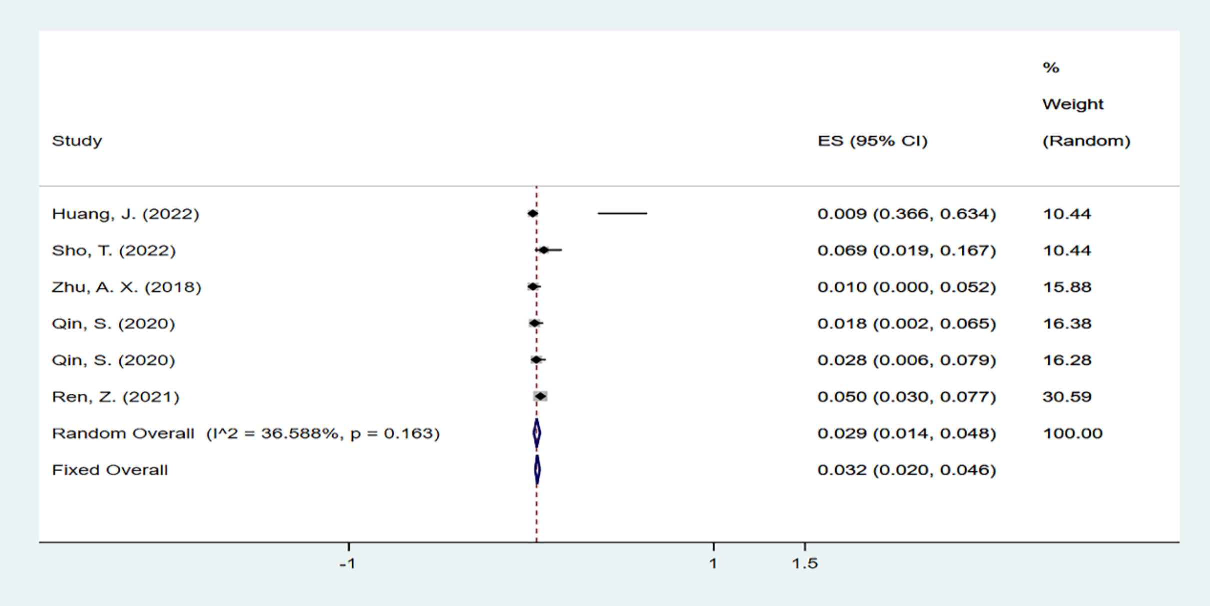

Supplementary Figure S44 Meta-analysis of the incidence of grade ≥ 3 increased γ-glutamyltransferase

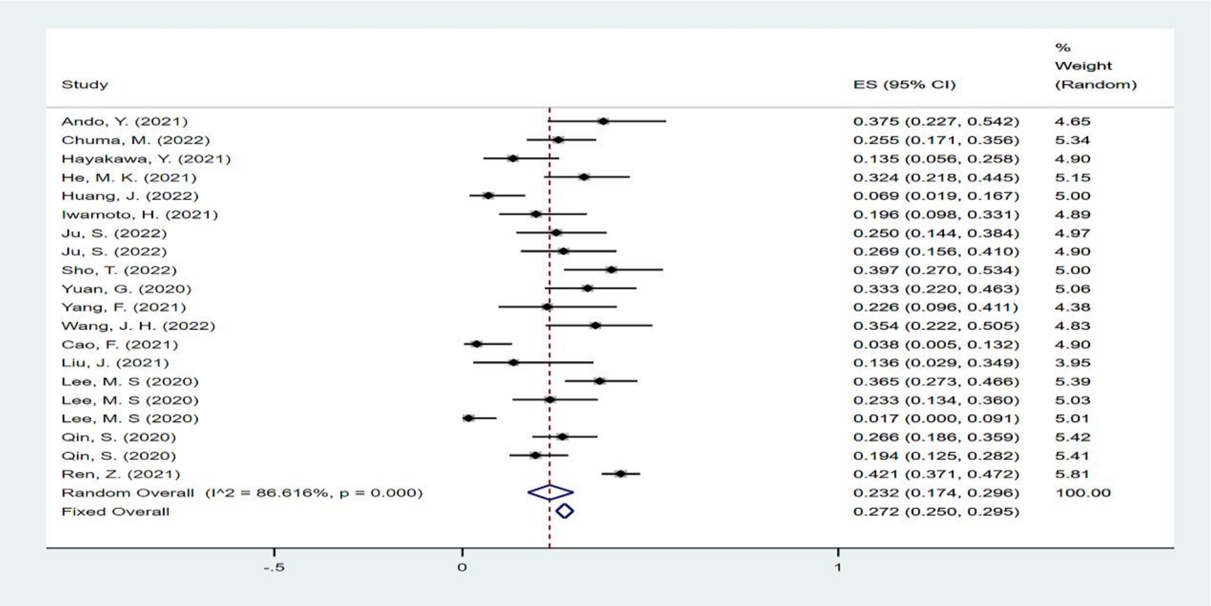

Supplementary Figure S45 Meta-analysis of the incidence of any-grade proteinuria

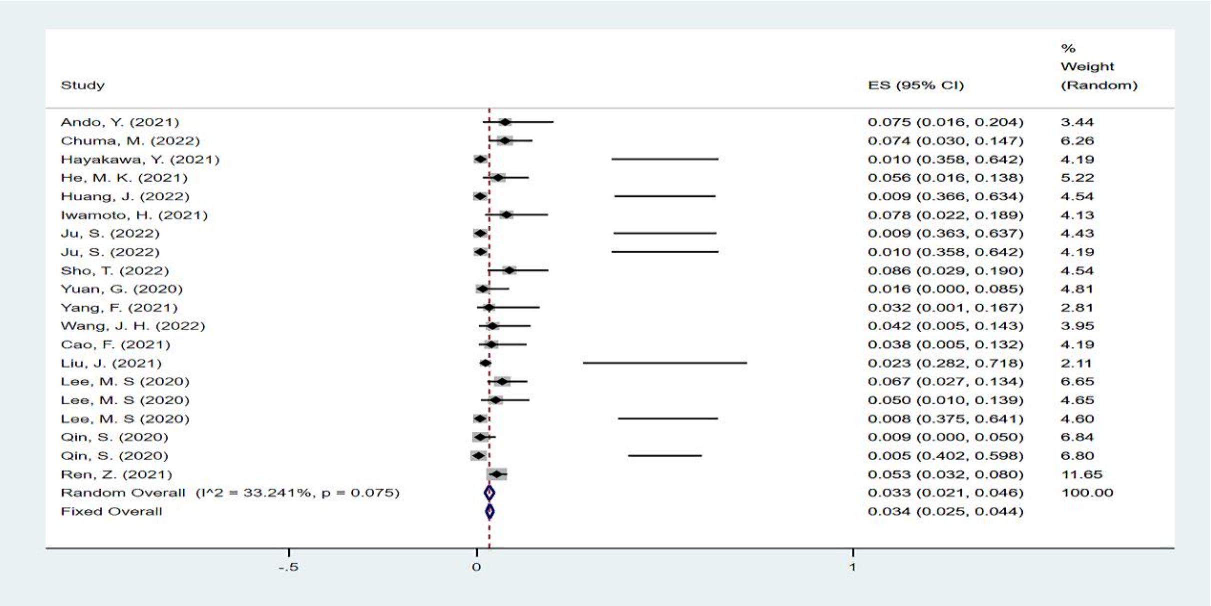

Supplementary Figure S46 Meta-analysis of the incidence of grade ≥ 3 proteinuria

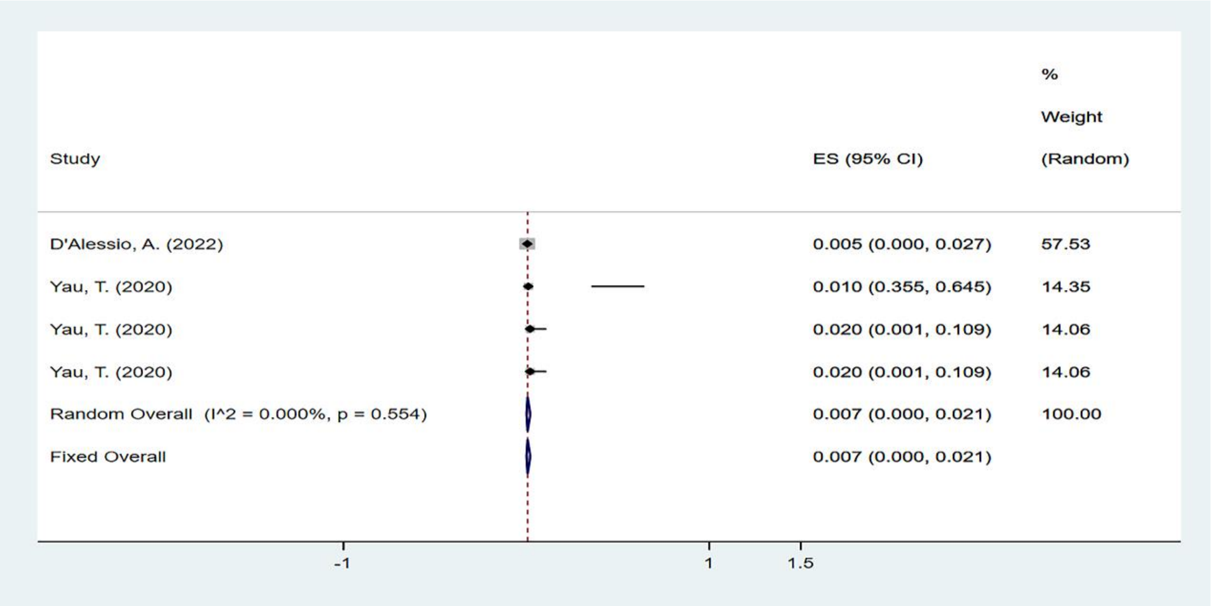

Supplementary Figure S47 Meta-analysis of the incidence of any-grade nephritis

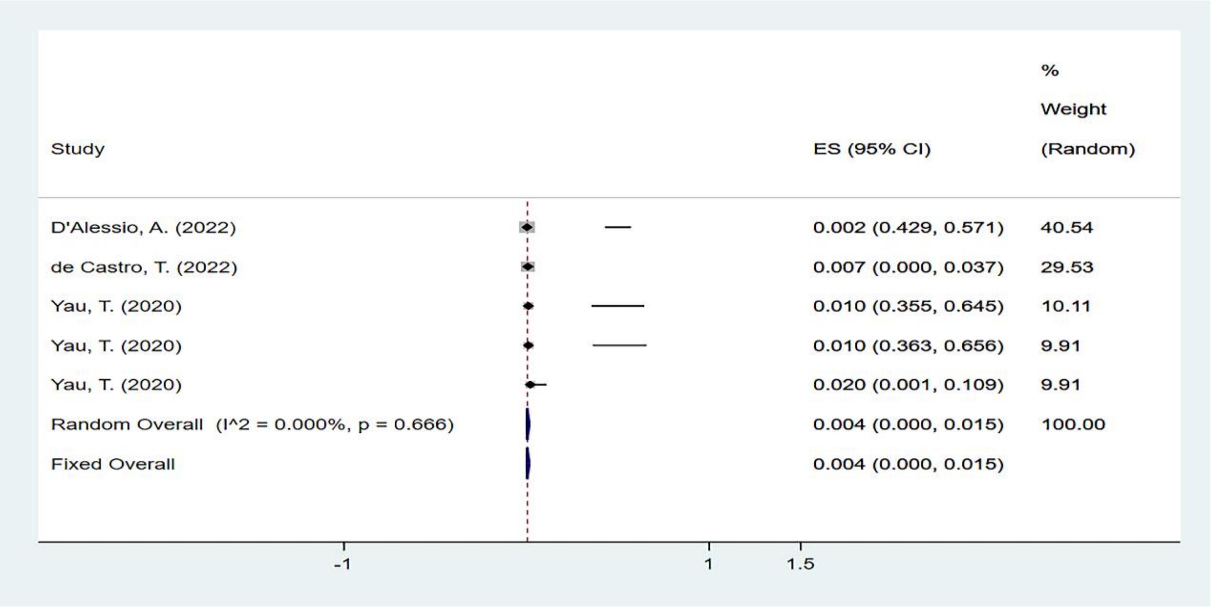

Supplementary Figure S48 Meta-analysis of the incidence of grade ≥ 3 nephritis

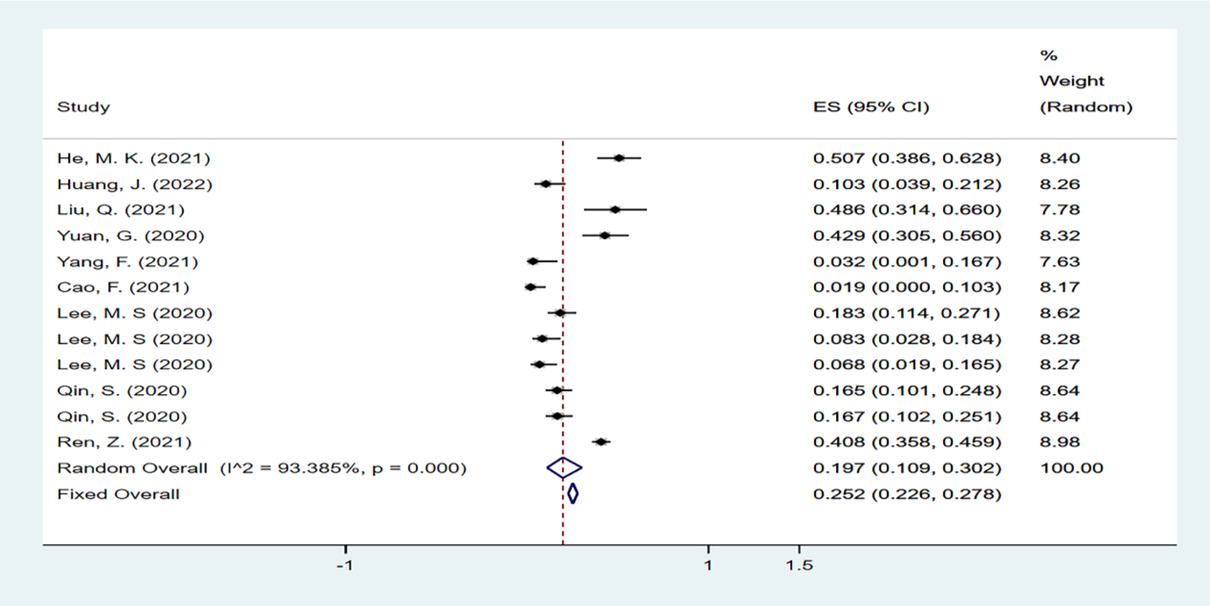

Supplementary Figure S49 Meta-analysis of the incidence of any-grade thrombocytopaenia

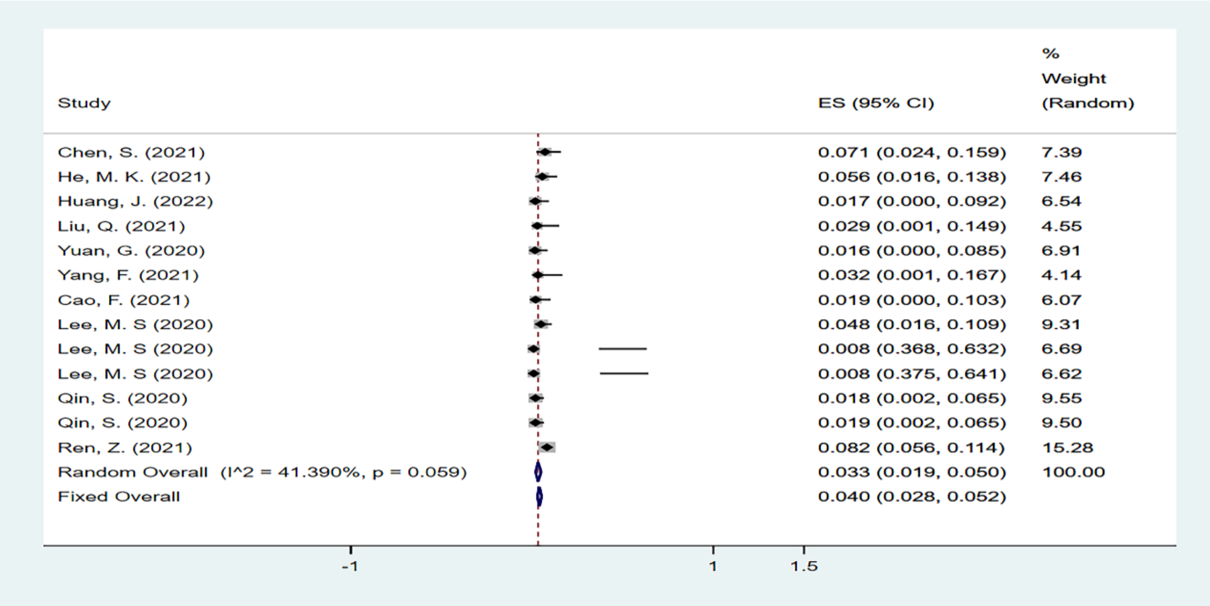

Supplementary Figure S50 Meta-analysis of the incidence of grade ≥ 3 thrombocytopaenia

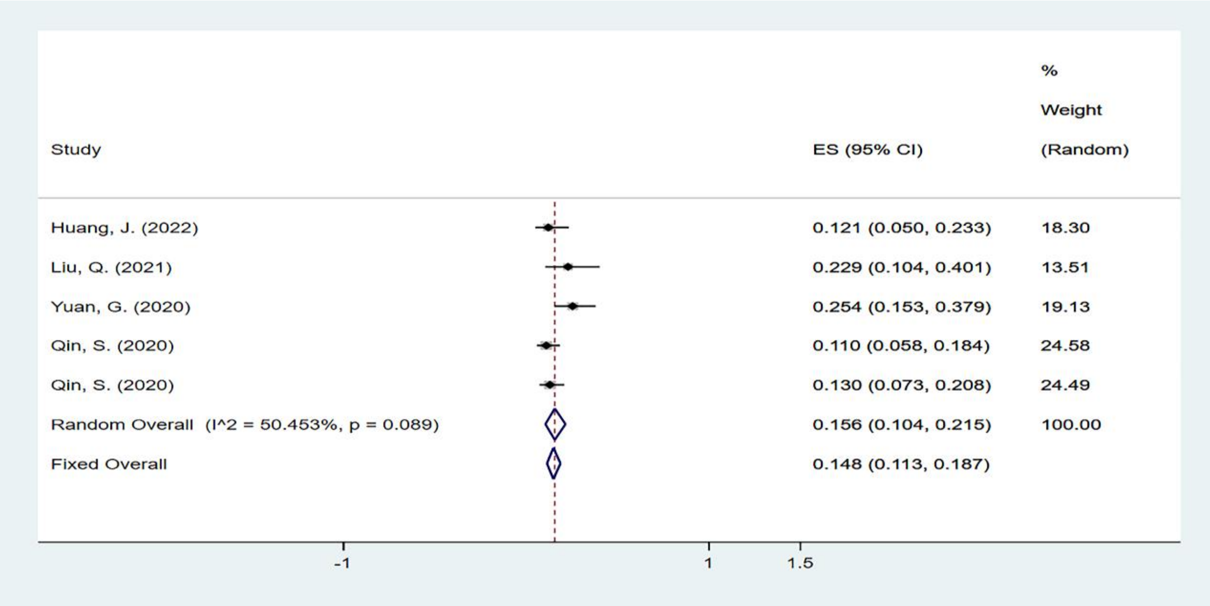

Supplementary Figure S51 Meta-analysis of the incidence of any-grade leukopenia

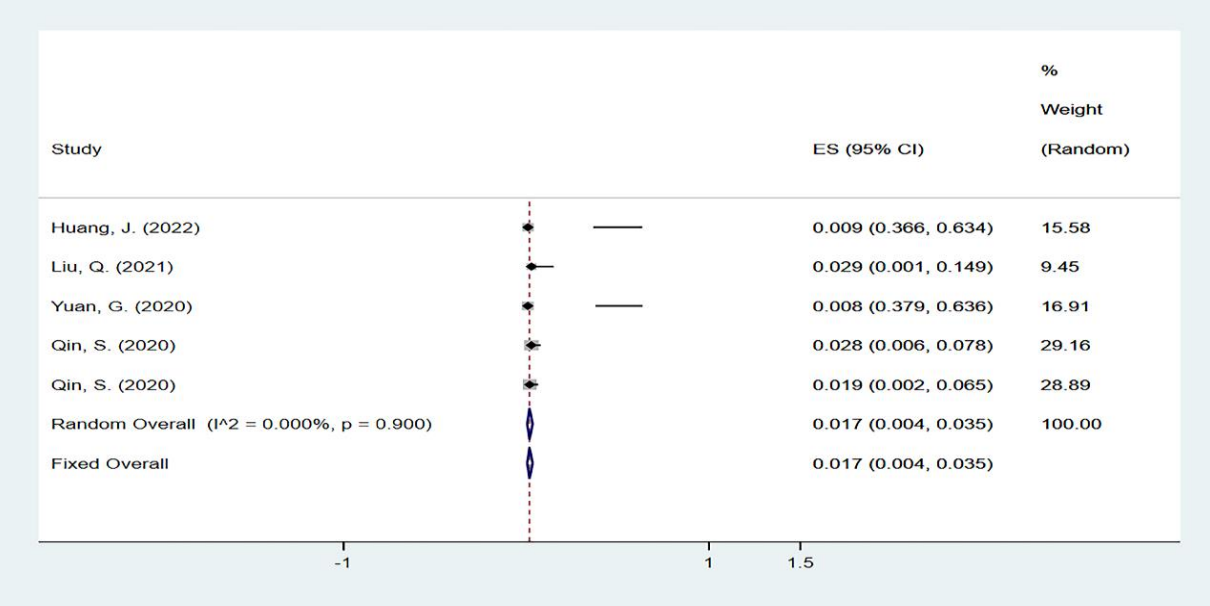

Supplementary Figure S52 Meta-analysis of the incidence of grade ≥ 3 leukopenia

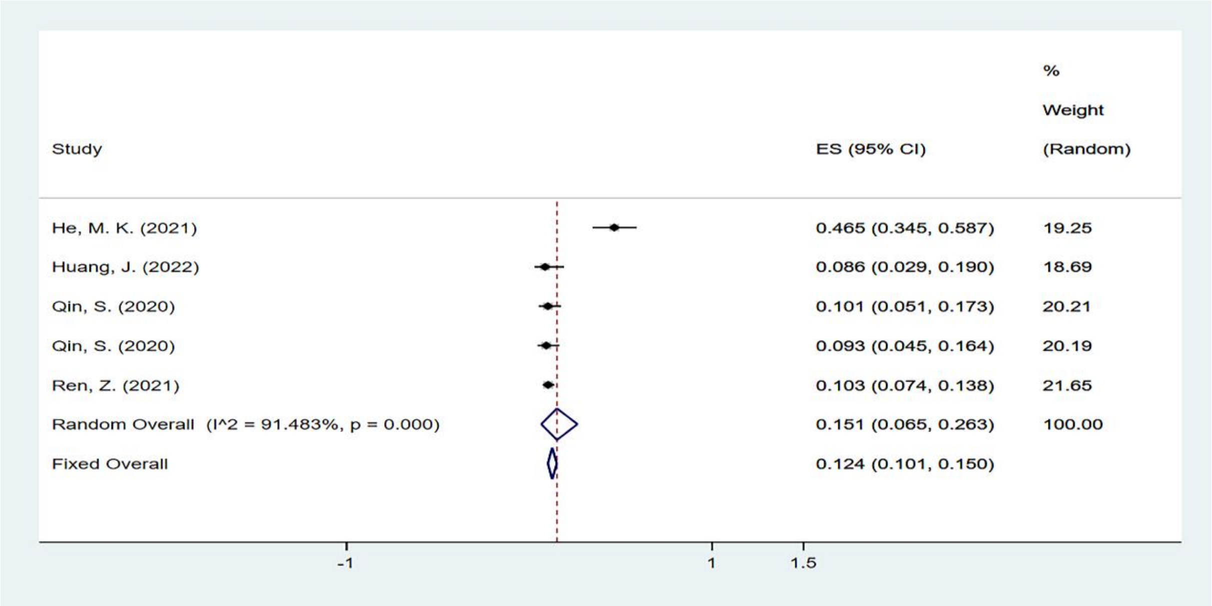

Supplementary Figure S53 Meta-analysis of the incidence of any-grade neutropenia

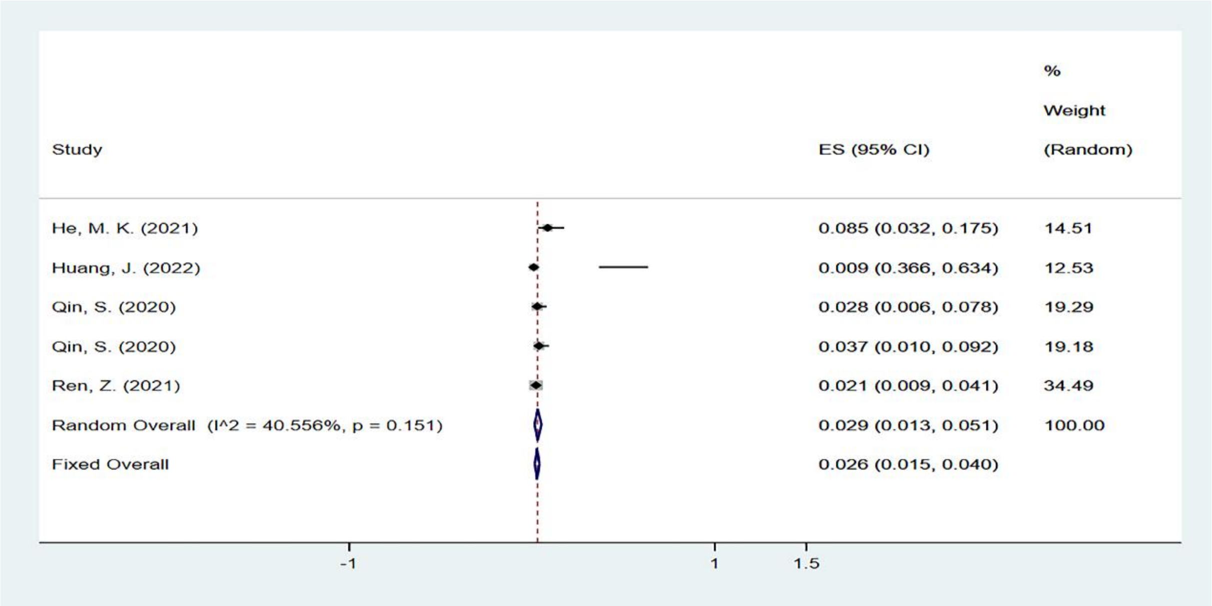

Supplementary Figure S54 Meta-analysis of the incidence of grade ≥ 3 neutropenia

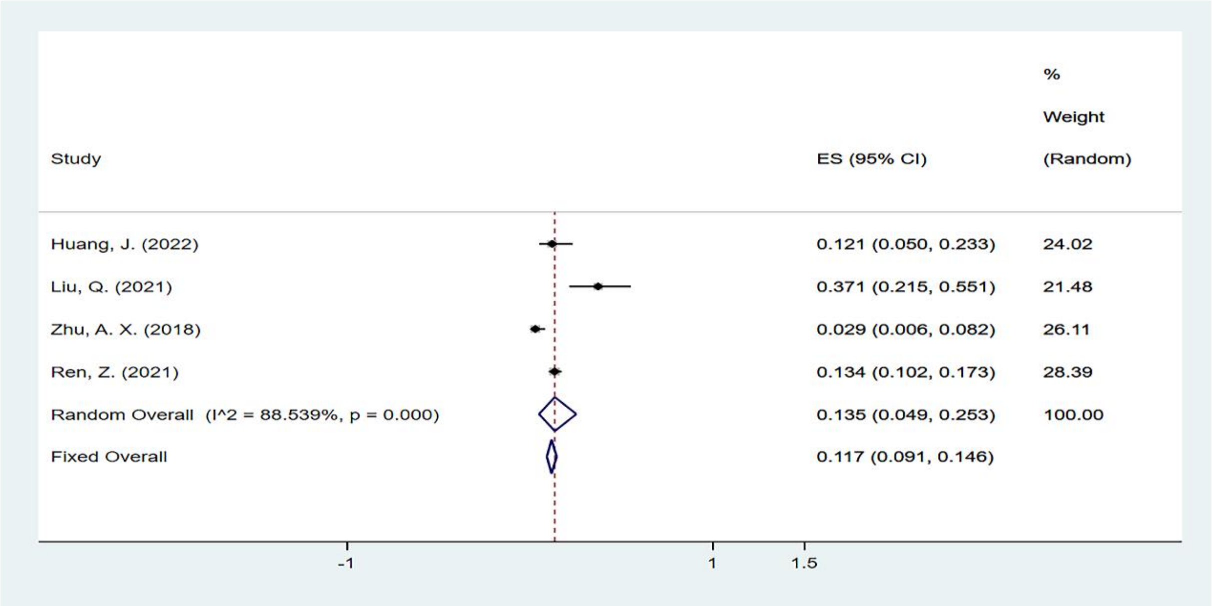

Supplementary Figure S55 Meta-analysis of the incidence of any-grade anemia

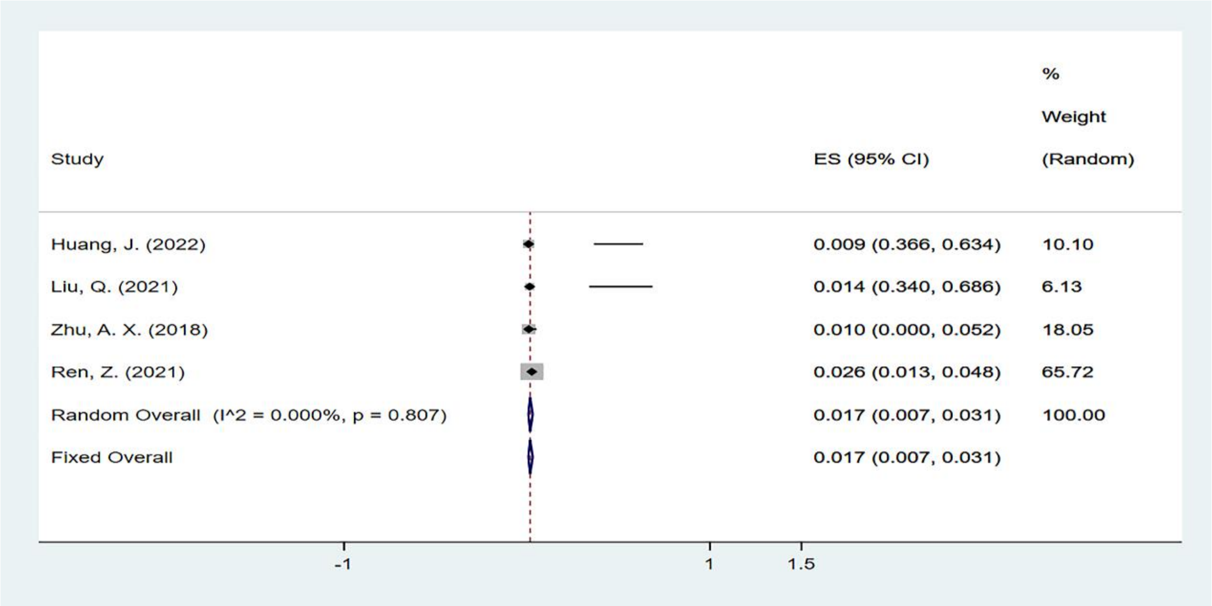

Supplementary Figure S56 Meta-analysis of the incidence of grade ≥ 3 anemia

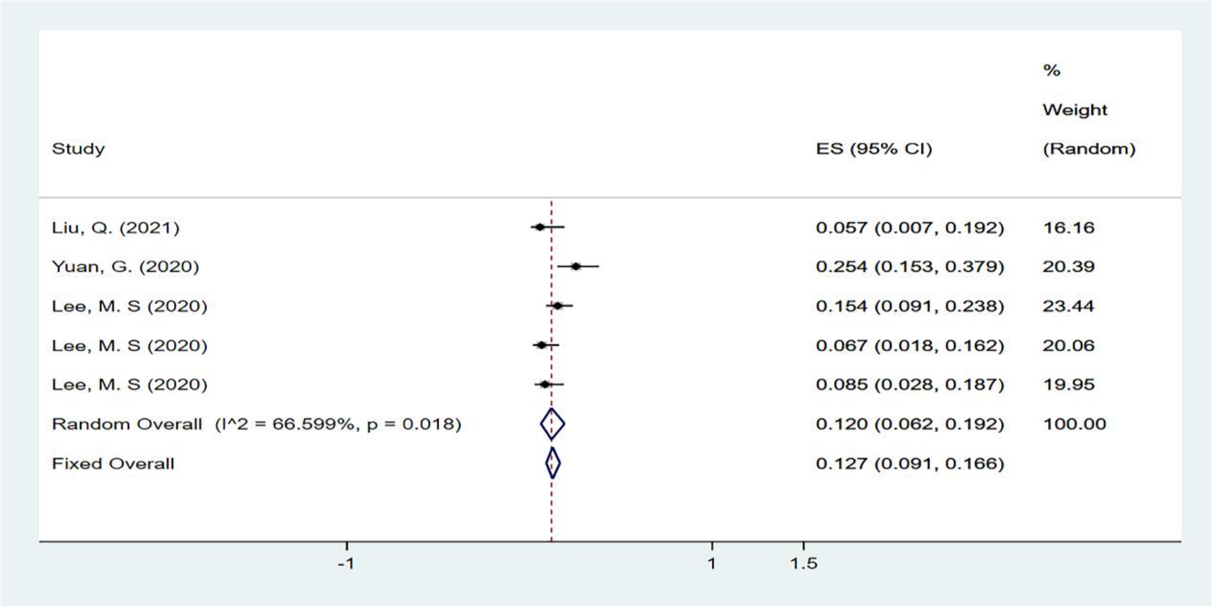

Supplementary Figure S57 Meta-analysis of the incidence of any-grade cough

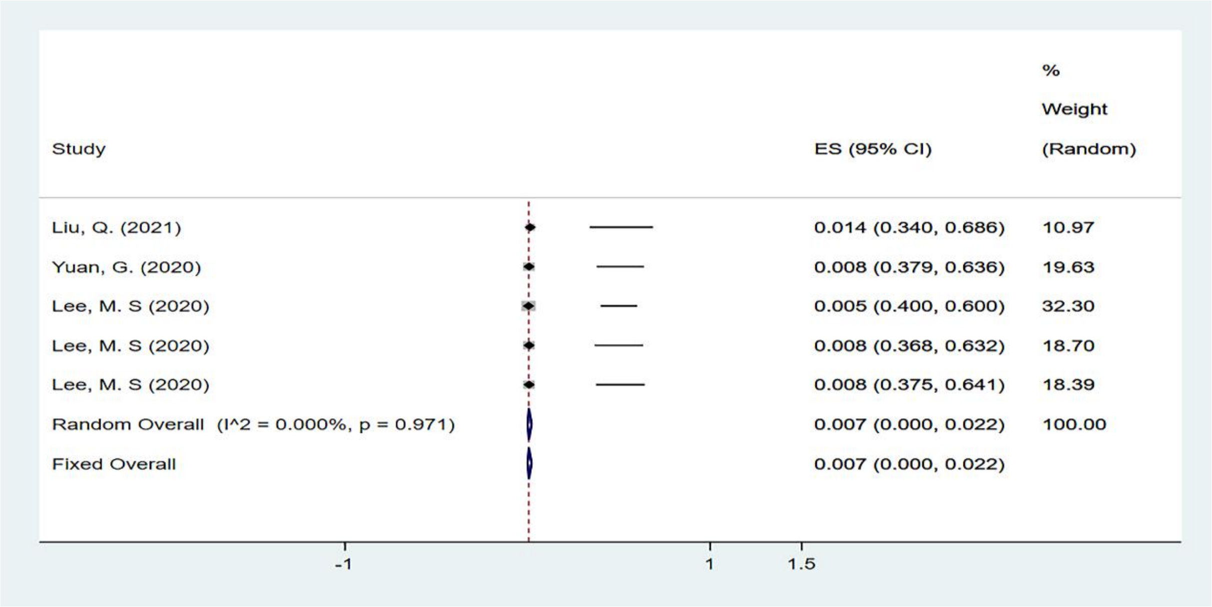

Supplementary Figure S58 Meta-analysis of the incidence of grade ≥ 3 cough

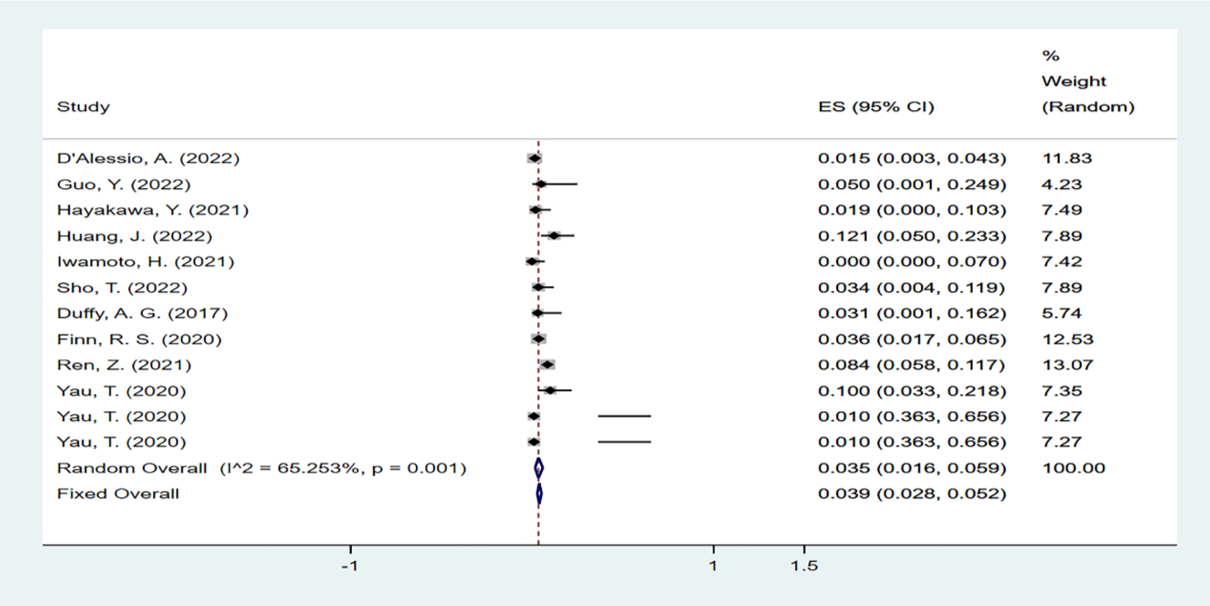

Supplementary Figure S59 Meta-analysis of the incidence of any-grade pneumonitis

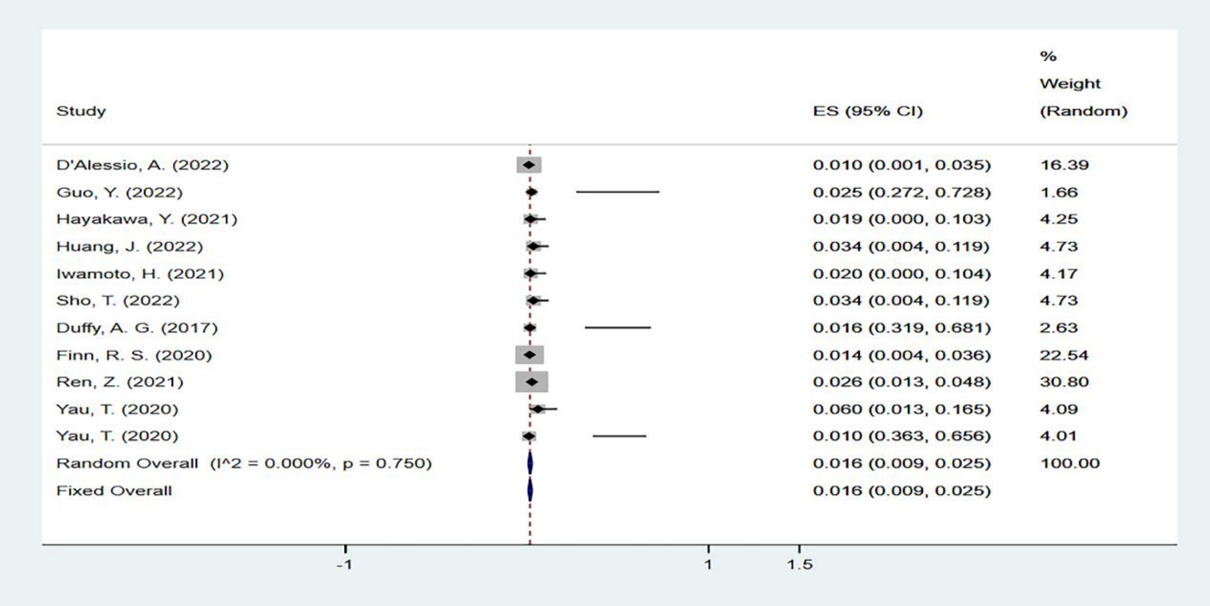

Supplementary Figure S60 Meta-analysis of the incidence of grade ≥ 3 pneumonitis

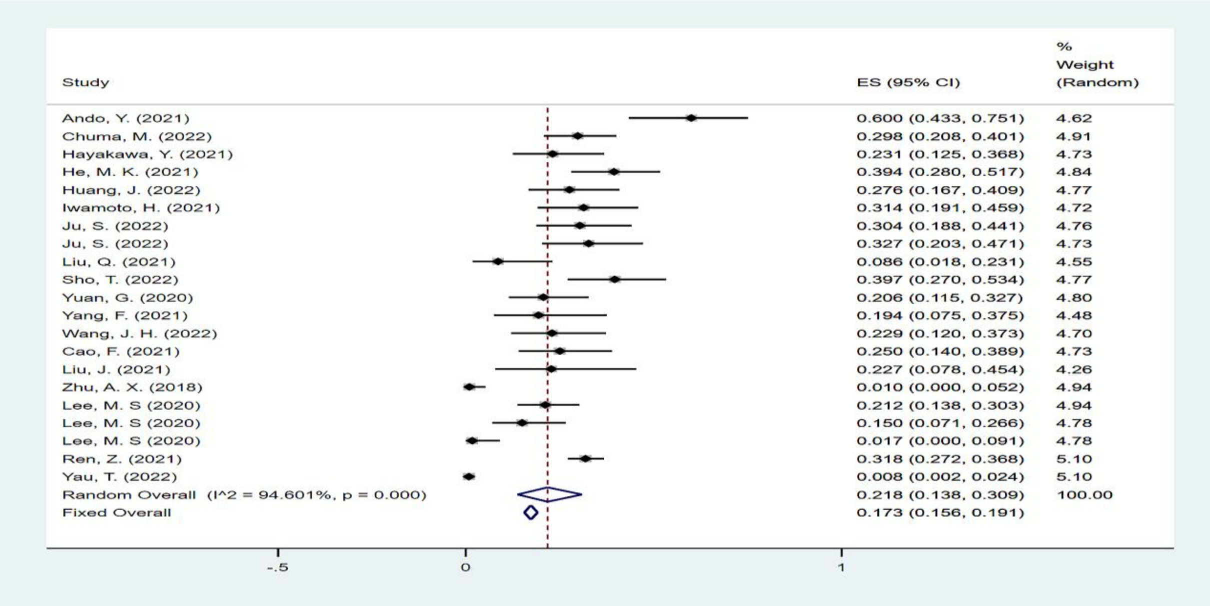

Supplementary Figure S61 Meta-analysis of the incidence of any-grade hypertension

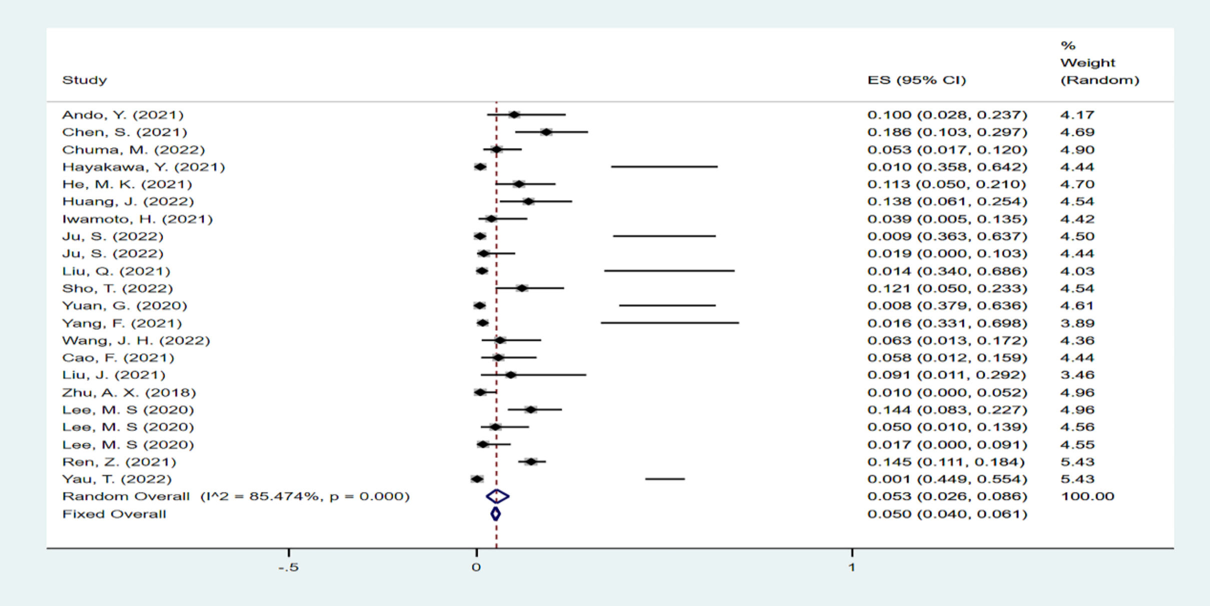

Supplementary Figure S62 Meta-analysis of the incidence of grade ≥ 3 hypertension

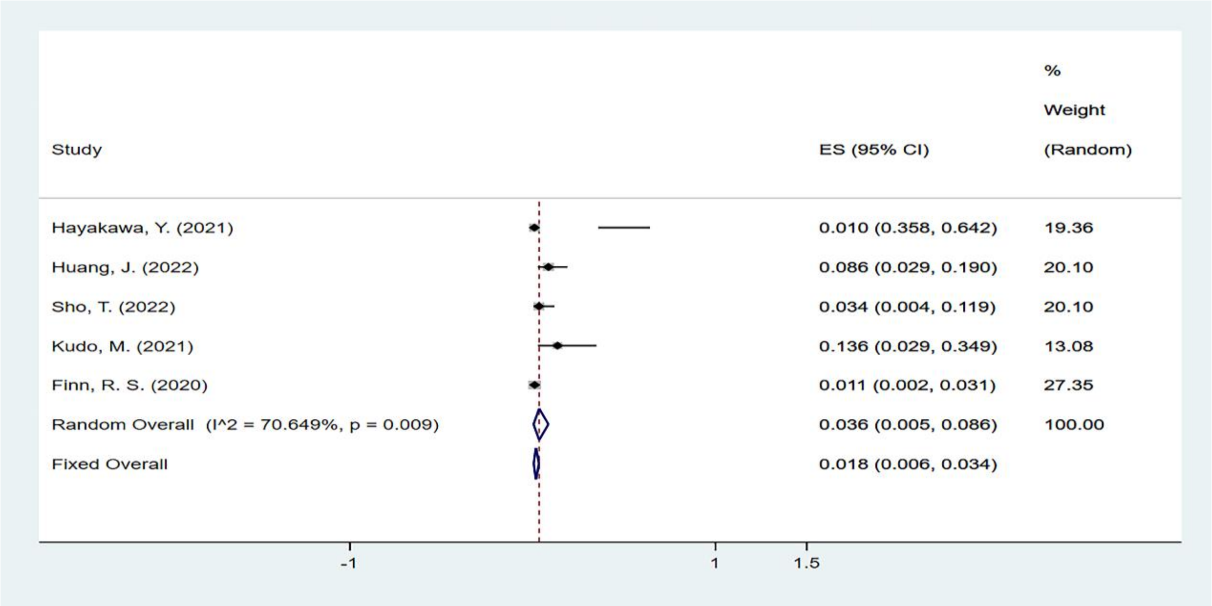

Supplementary Figure S63 Meta-analysis of the incidence of any-grade infusion reaction

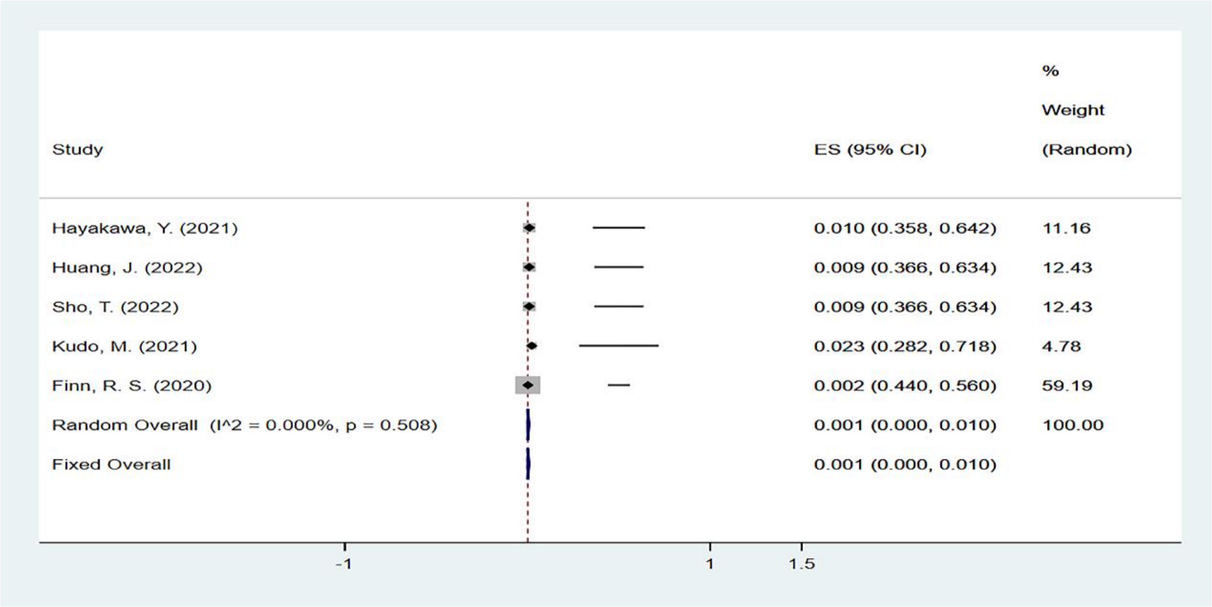

Supplementary Figure S64 Meta-analysis of the incidence of grade ≥ 3 infusion reaction

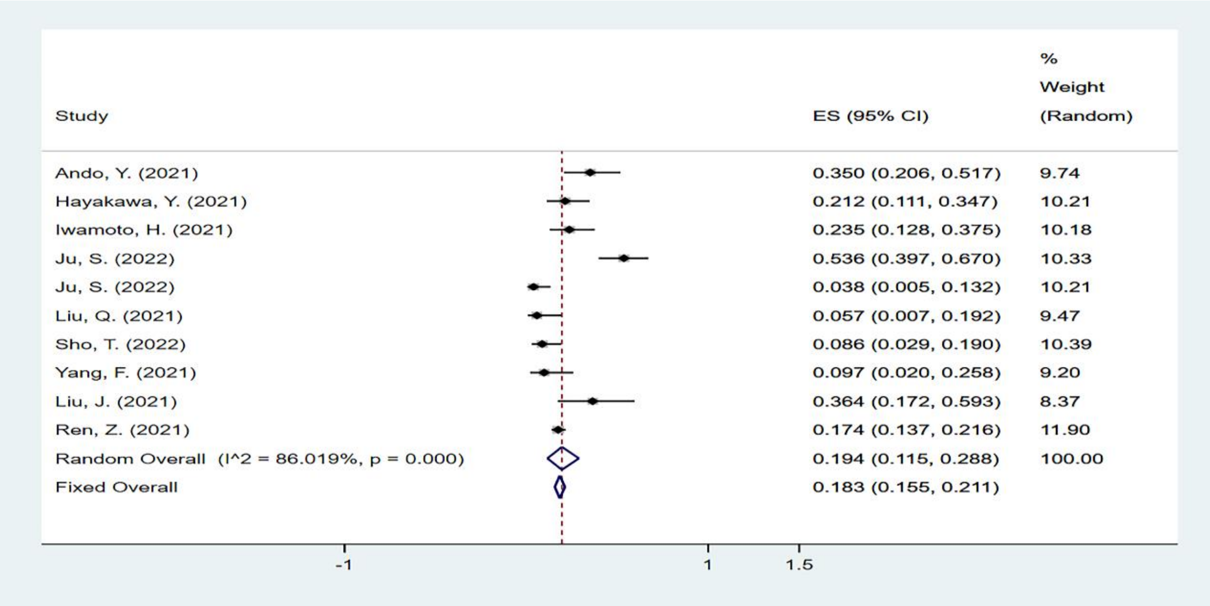

Supplementary Figure S65 Meta-analysis of the incidence of any-grade fever

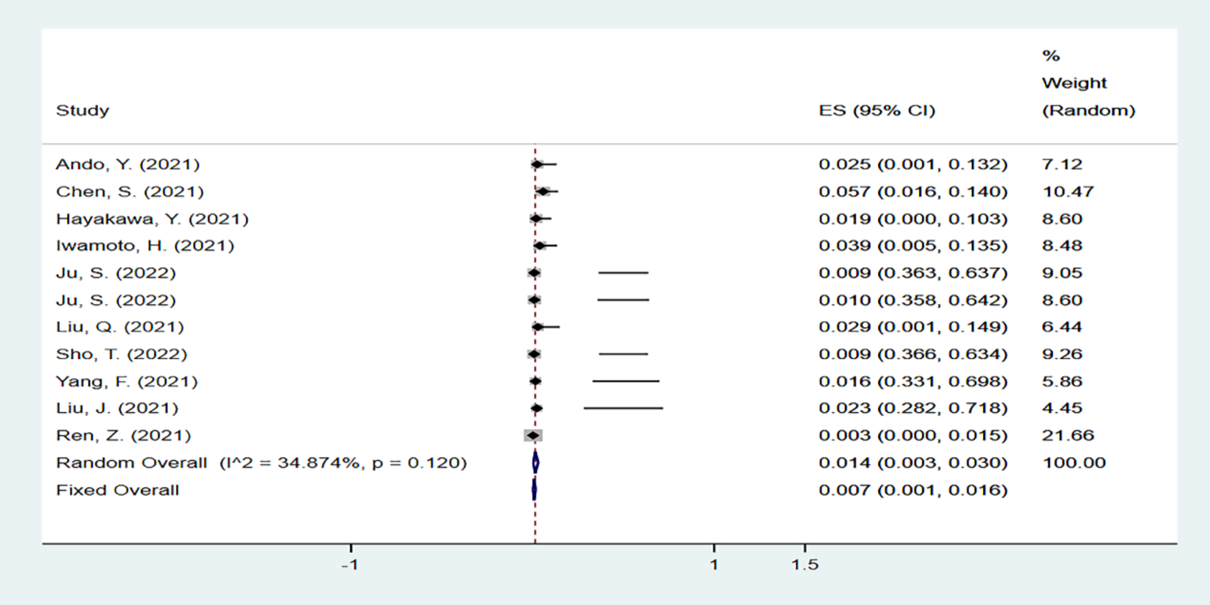

Supplementary Figure S66 Meta-analysis of the incidence of grade ≥ 3 fever

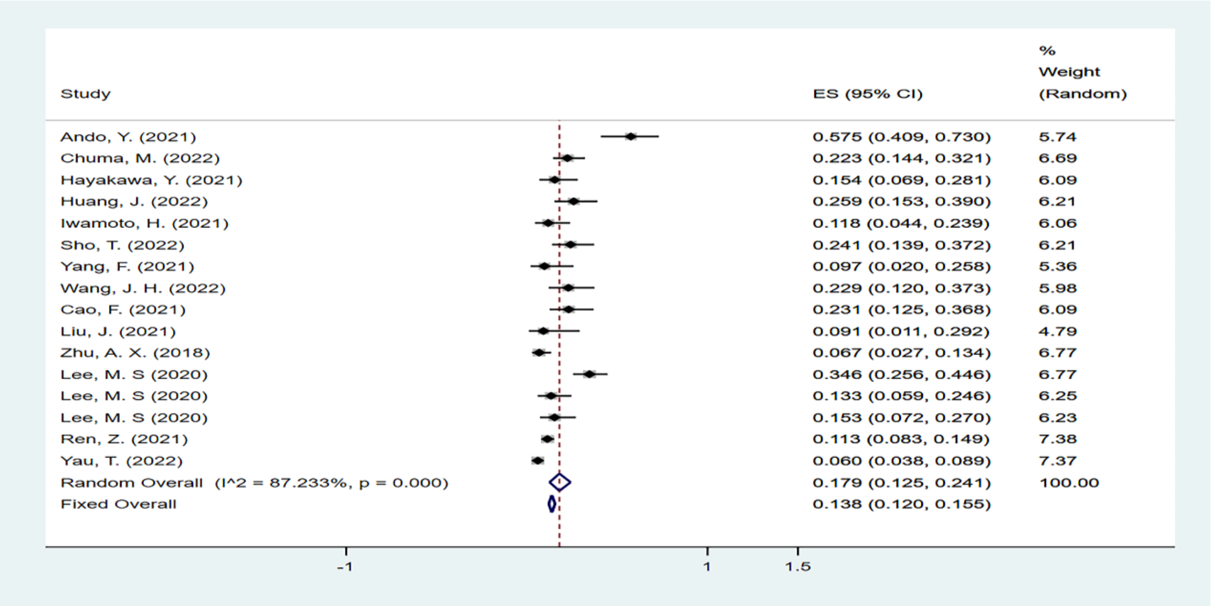

Supplementary Figure S67 Meta-analysis of the incidence of any-grade decreased appetite

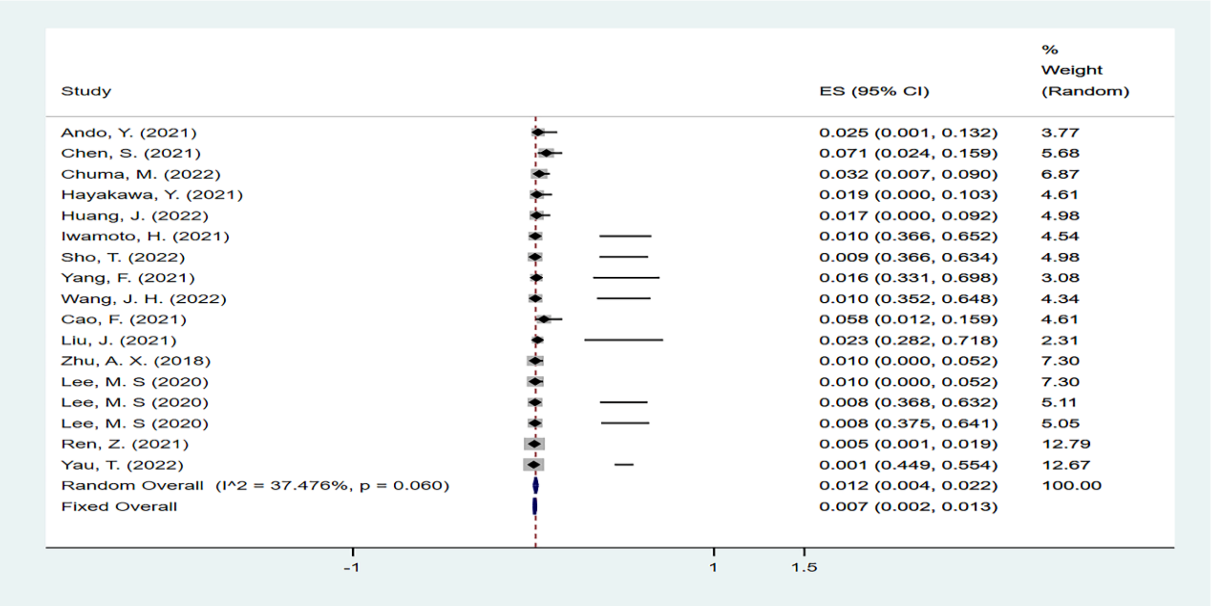

Supplementary Figure S68 Meta-analysis of the incidence of grade ≥ 3 decreased appetite

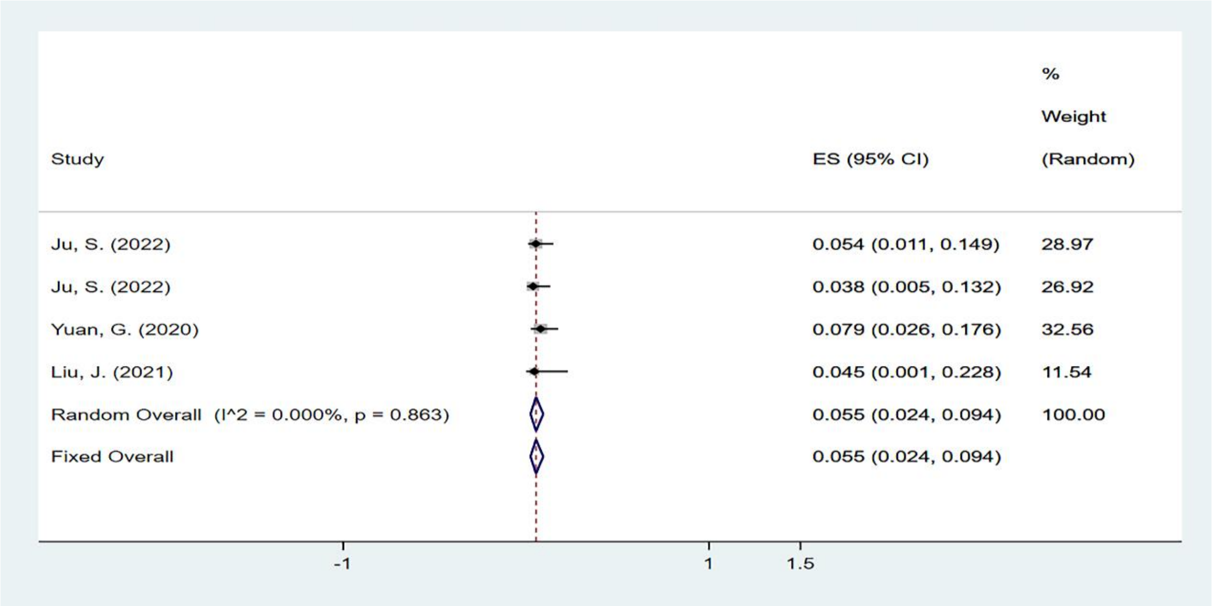

Supplementary Figure S69 Meta-analysis of the incidence of any-grade mouth ulcers

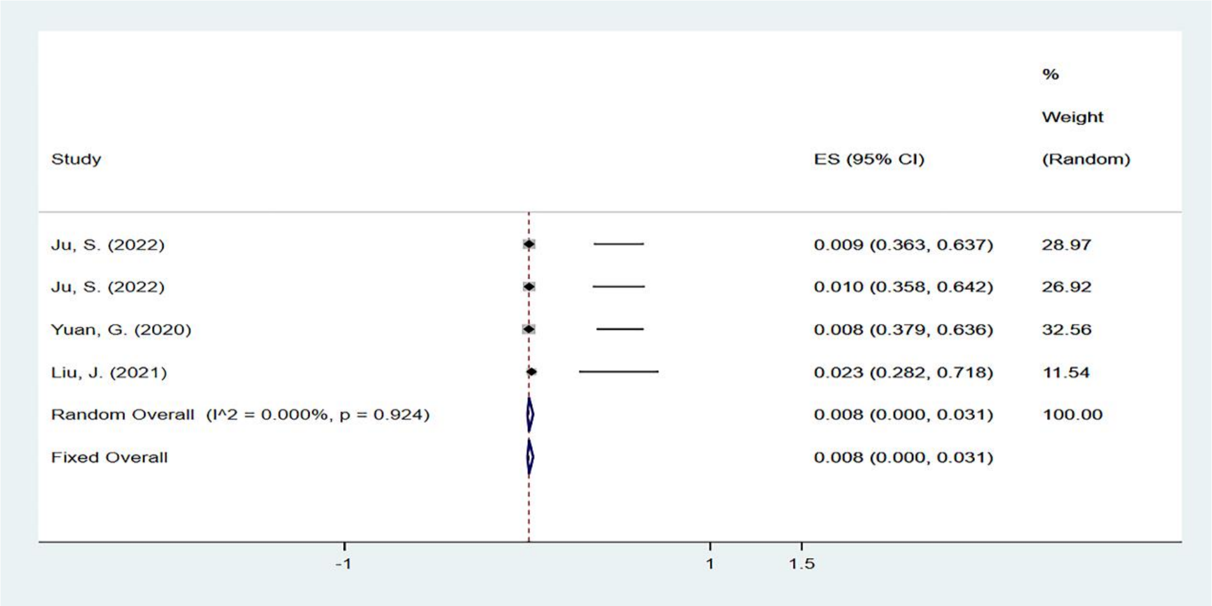

Supplementary Figure S70 Meta-analysis of the incidence of grade ≥ 3 mouth ulcers

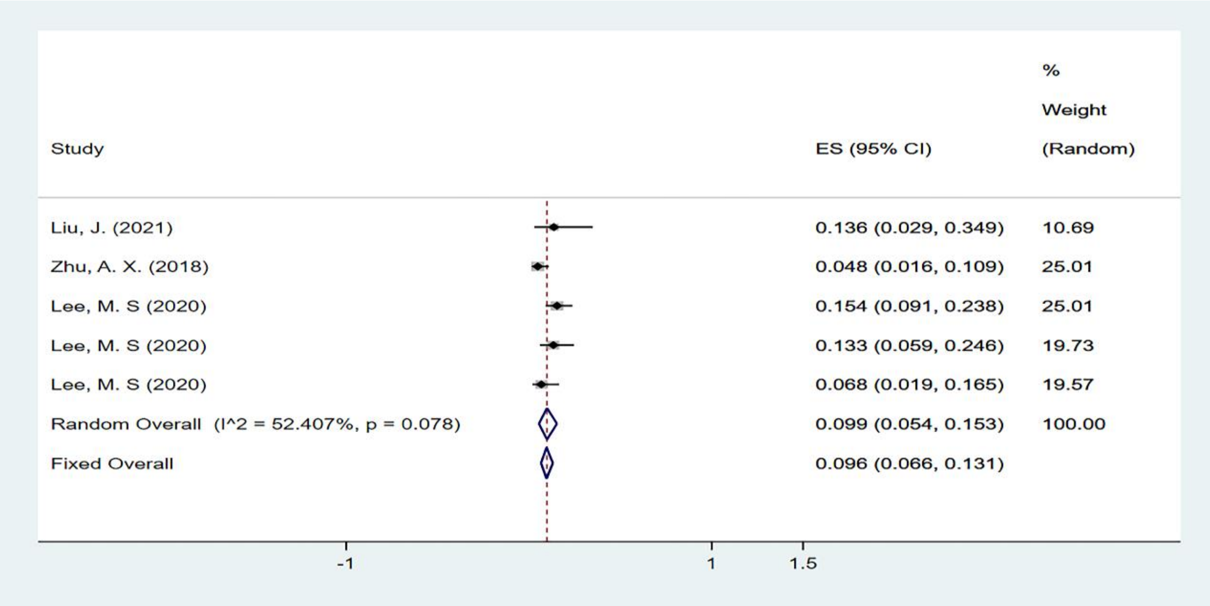

Supplementary Figure S71 Meta-analysis of the incidence of any-grade arthralgia

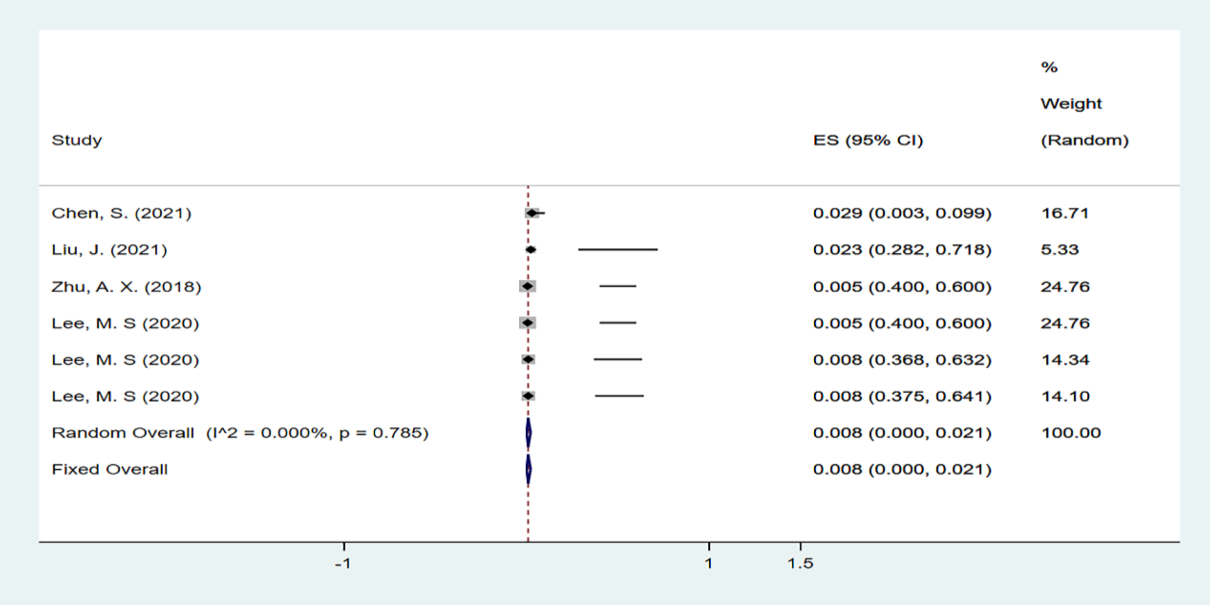

Supplementary Figure S72 Meta-analysis of the incidence of grade ≥ 3 arthralgia

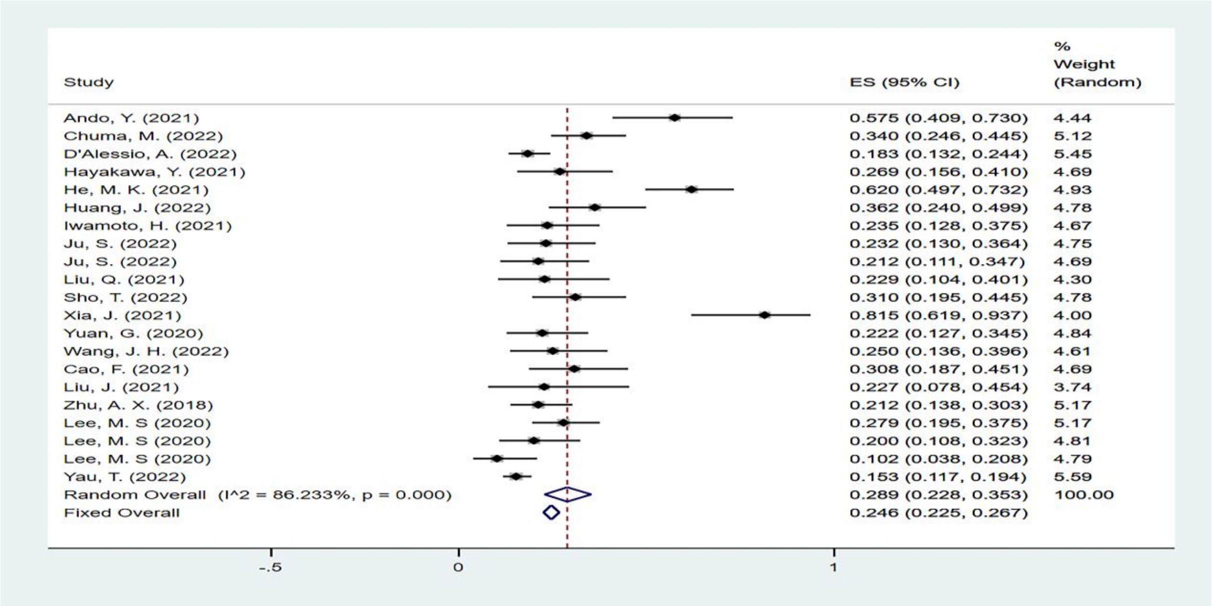

Supplementary Figure S73 Meta-analysis of the incidence of any-grade fatigue

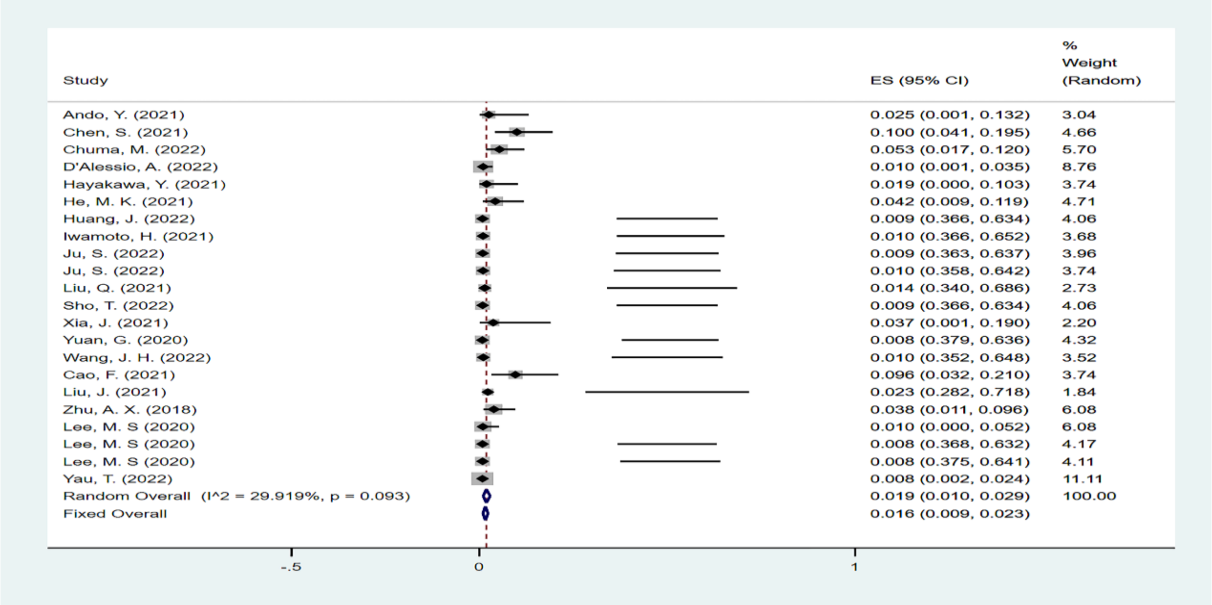

Supplementary Figure S74 Meta-analysis of the incidence of grade ≥ 3 fatigue

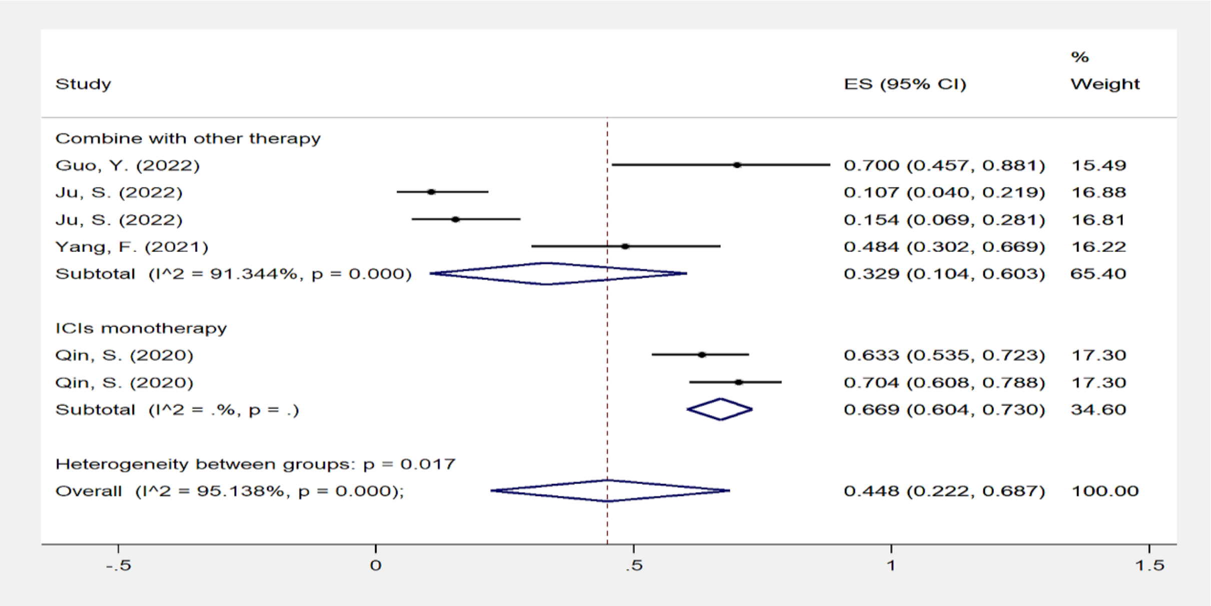

Supplementary Figure S75 Subgroup analysis of the incidence of any-grade RCCEP

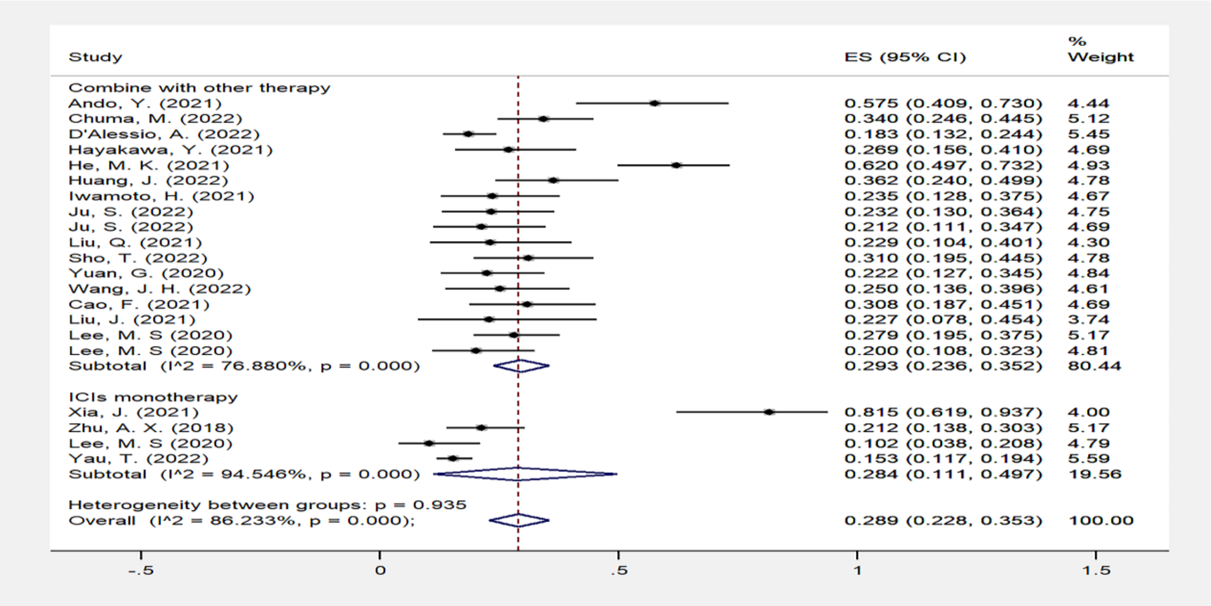

Supplementary Figure S76 Subgroup analysis of the incidence of any-grade fatigue

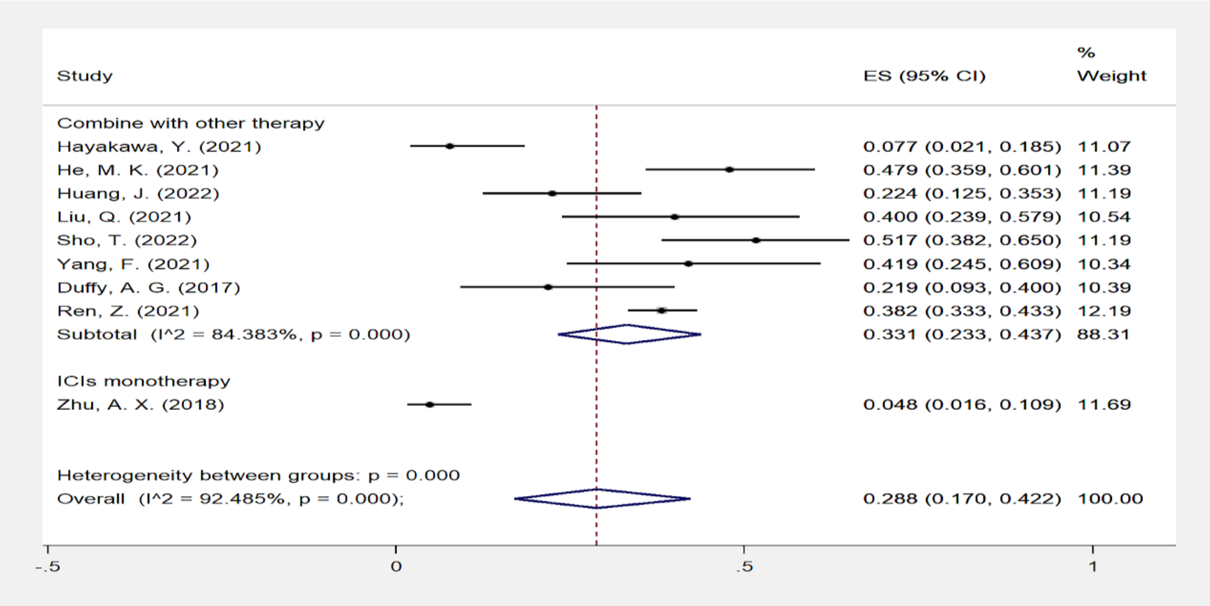

Supplementary Figure S77 Subgroup analysis of the incidence of any-grade hyperbilirubinemia

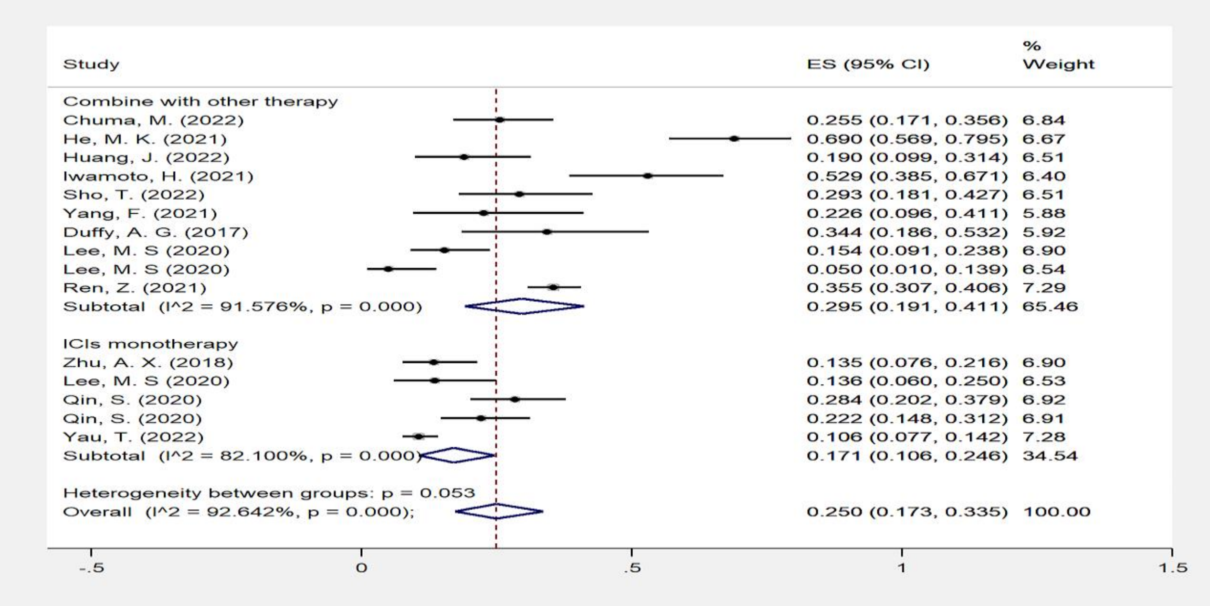

Supplementary Figure S78 Subgroup analysis of the incidence of any-grade elevated AST

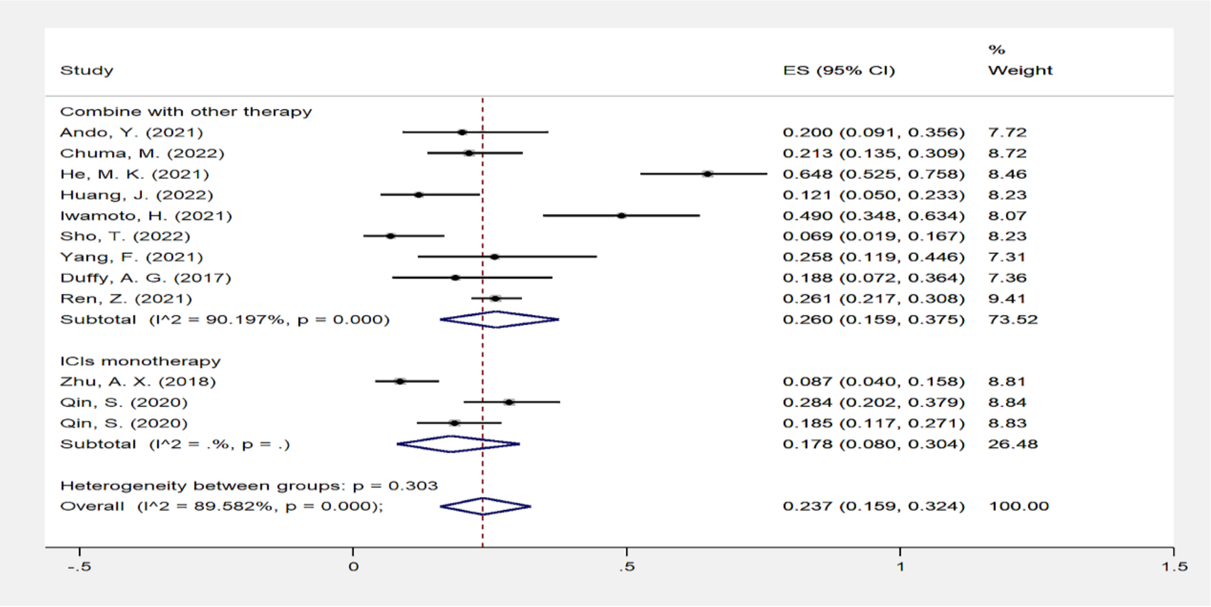

Supplementary Figure S79 Subgroup analysis of the incidence of any-grade elevated ALT

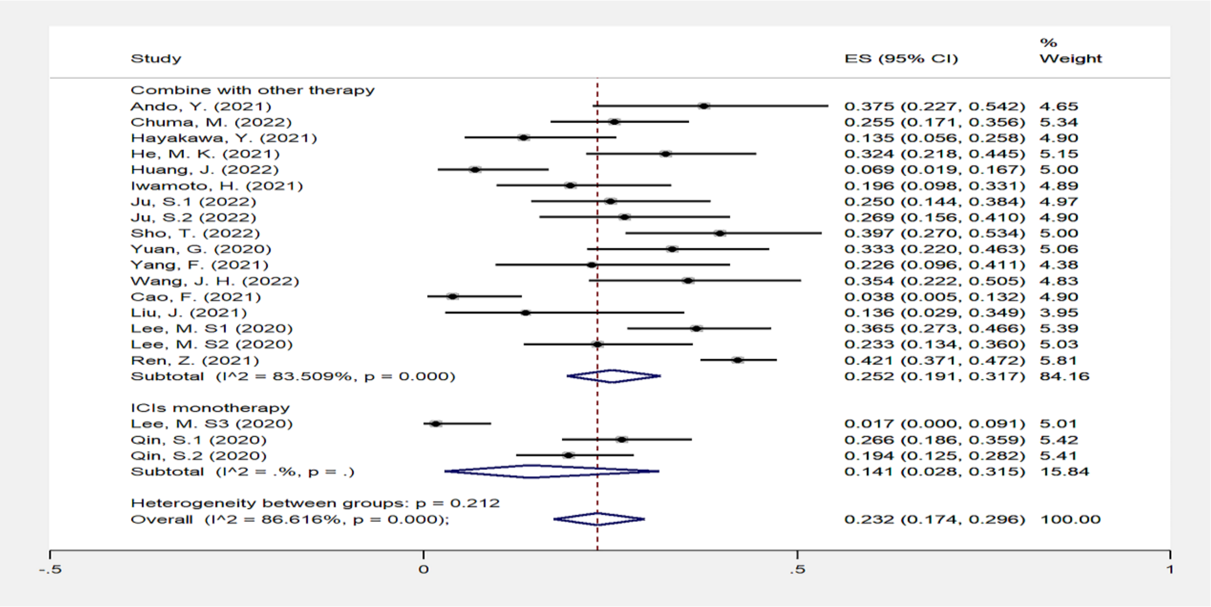

Supplementary Figure S80 Subgroup analysis of the incidence of any-grade proteinuria

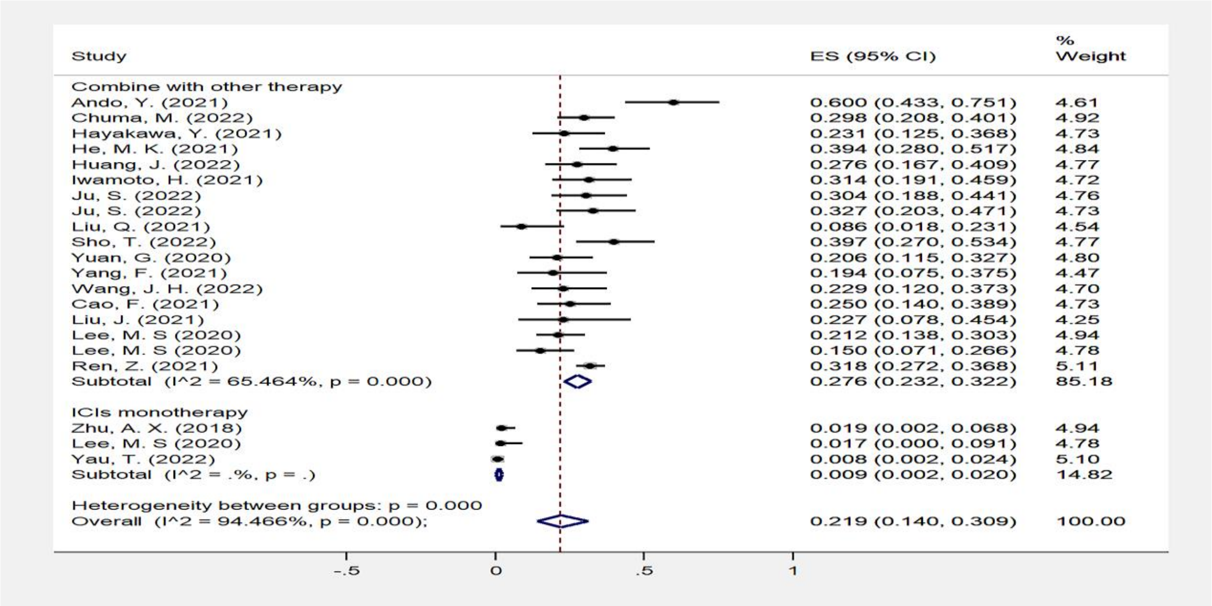

Supplementary Figure S81 Subgroup analysis of the incidence of any-grade hypertension

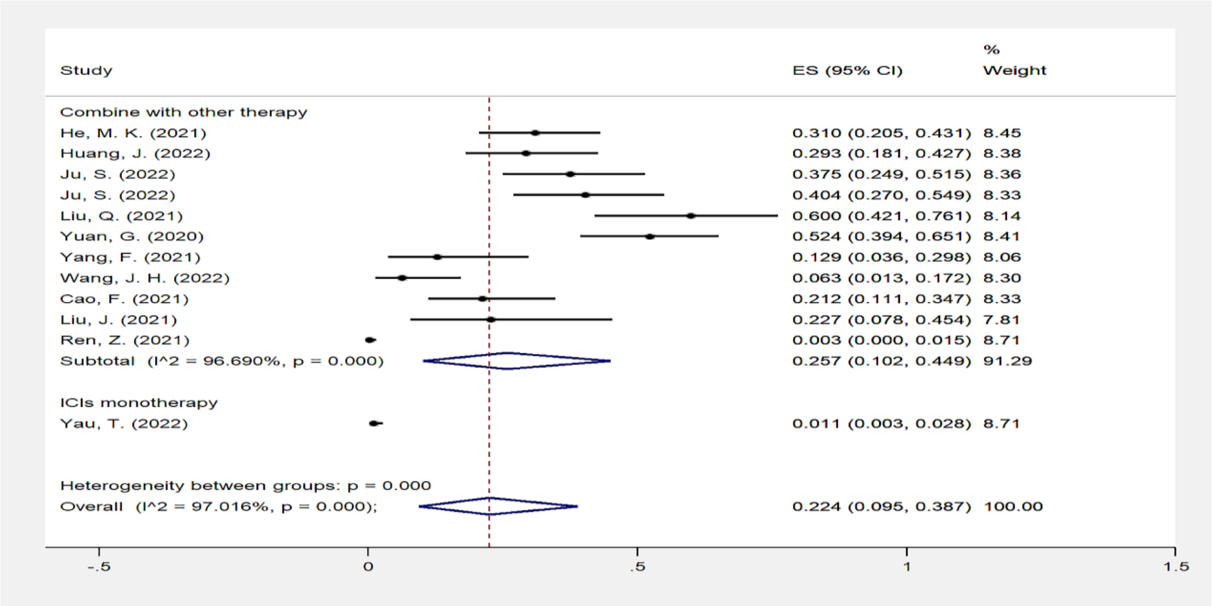

Supplementary Figure S82 Subgroup analysis of the incidence of any-grade hand-foot skin reaction

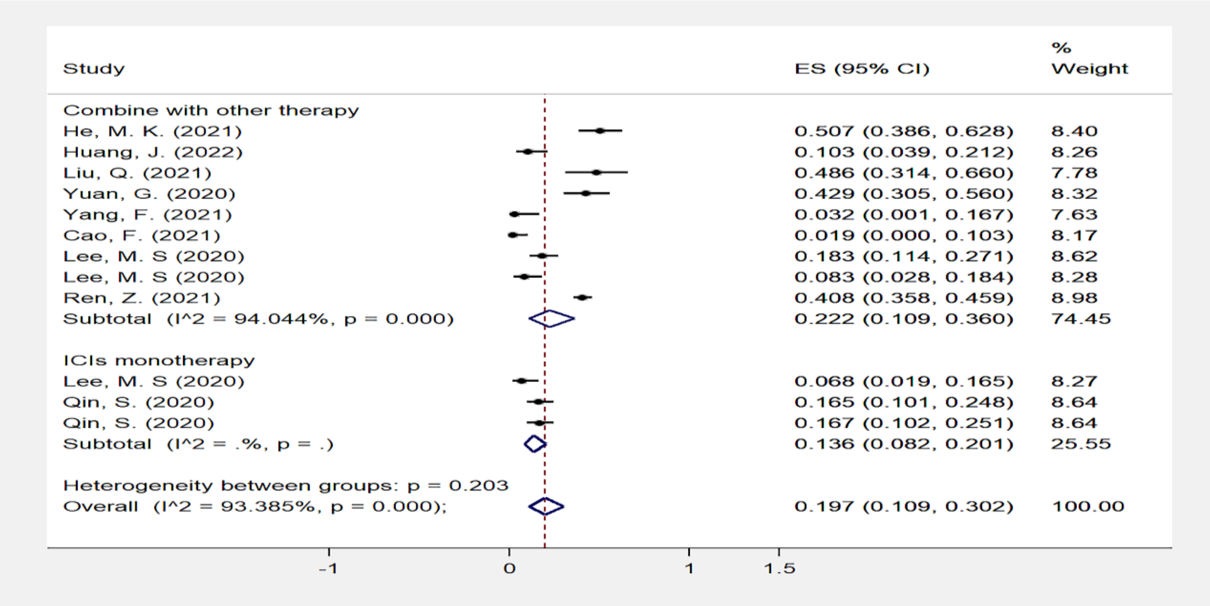

Supplementary Figure S83 Subgroup analysis of the incidence of any-grade thrombocytopaenia

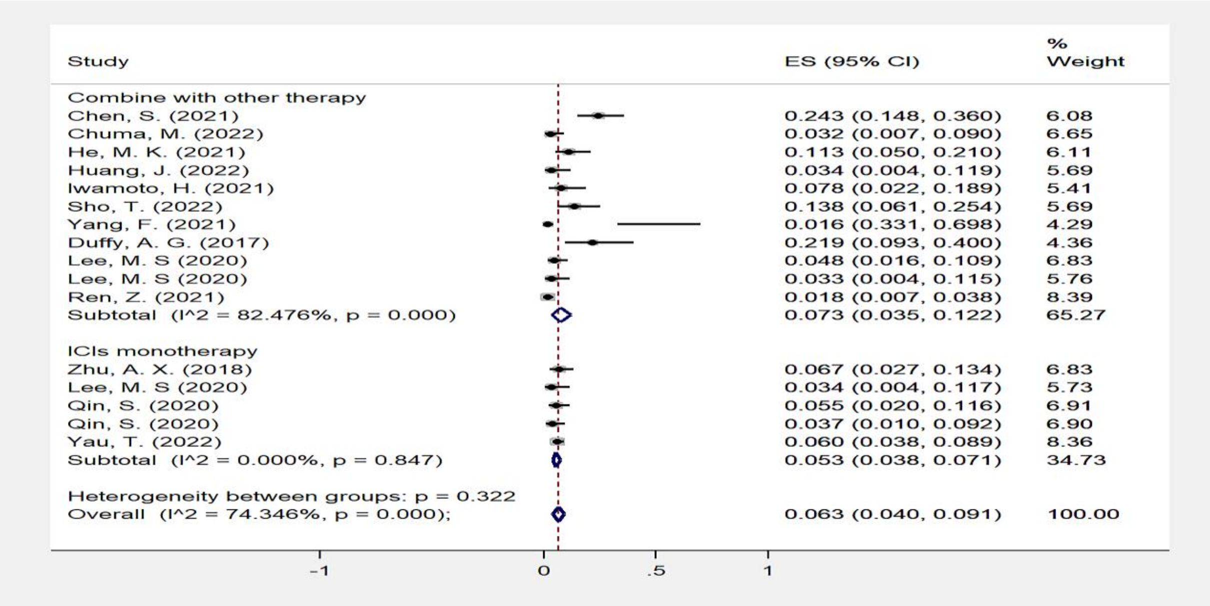

Supplementary Figure S84 Subgroup analysis of the incidence of grade ≥ 3 elevated AST

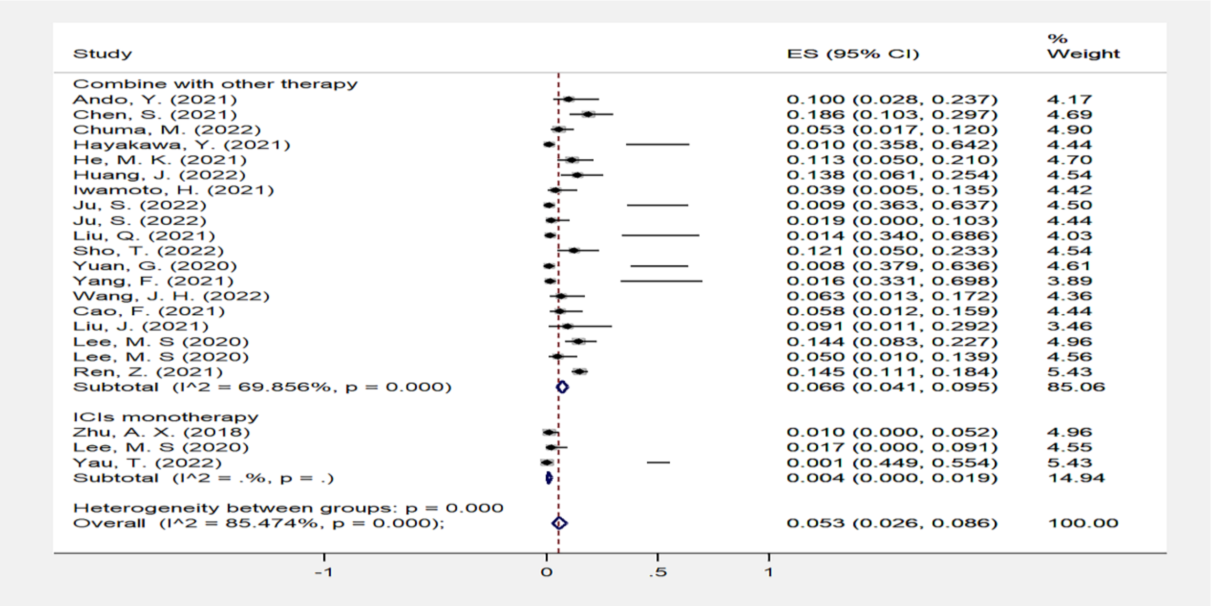

Supplementary Figure S85 Subgroup analysis of the incidence of grade ≥ 3 hypertension

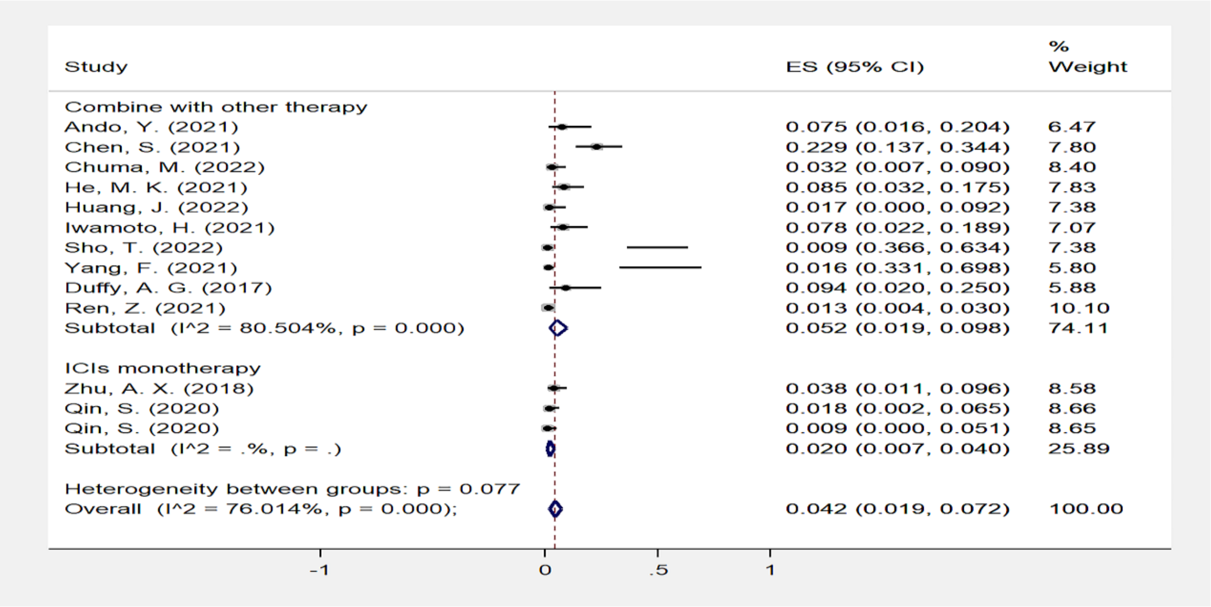

Supplementary Figure S86 Subgroup analysis of the incidence of grade ≥ 3 elevated ALT

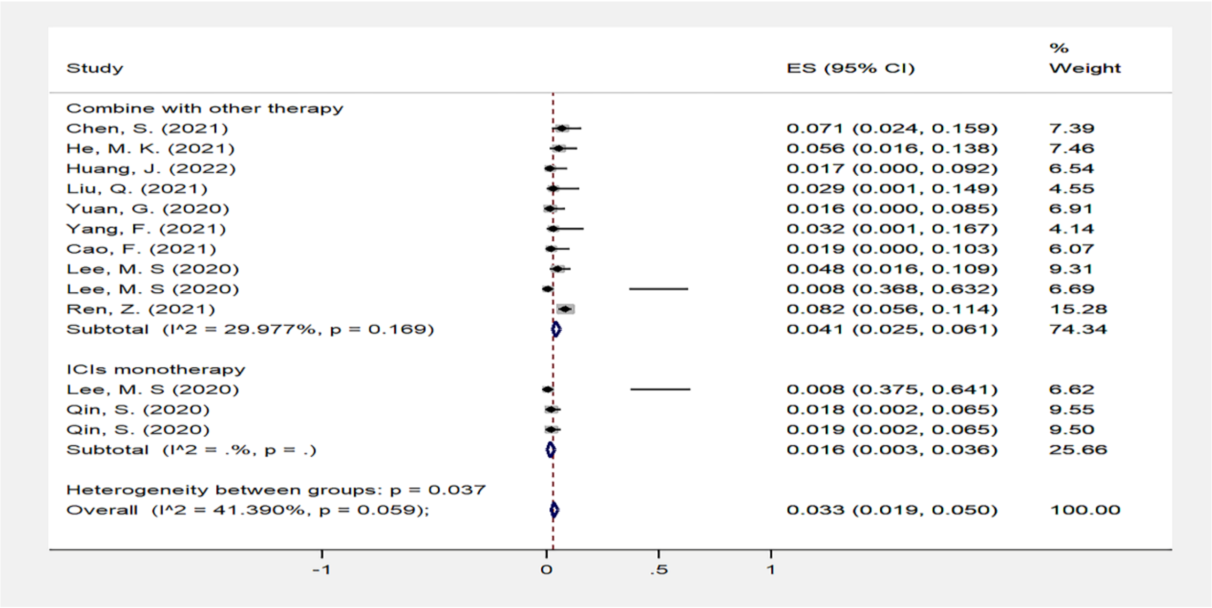

Supplementary Figure S87 Subgroup analysis of the incidence of grade ≥ 3 thrombocytopaenia

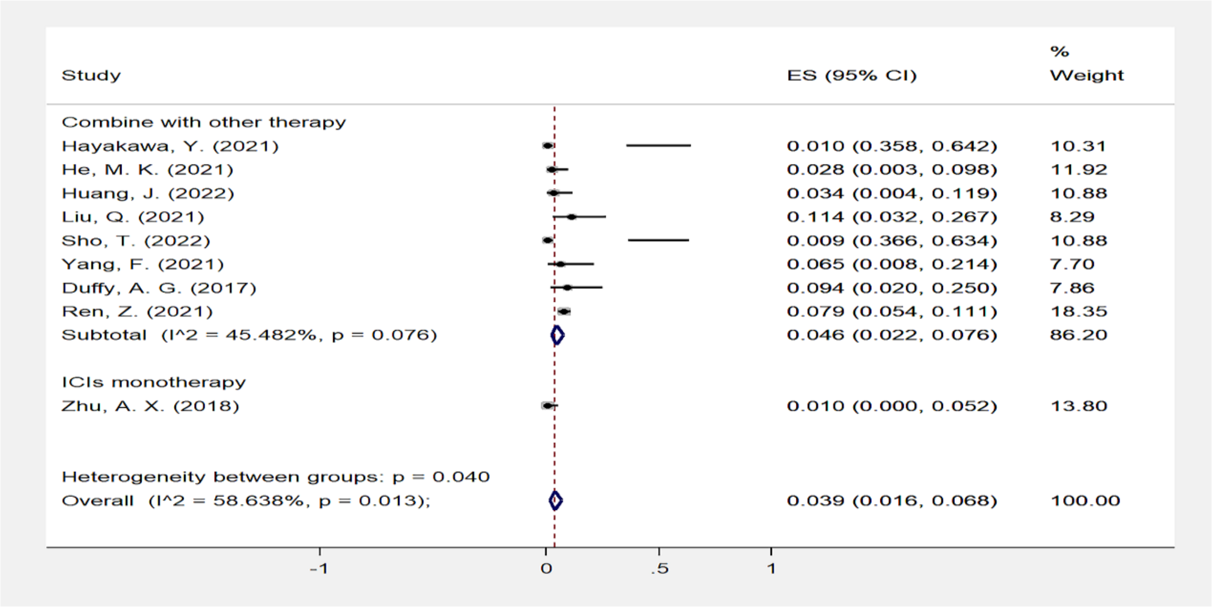

Supplementary Figure S88 Subgroup analysis of the incidence of grade ≥ 3 hyperbilirubinemia

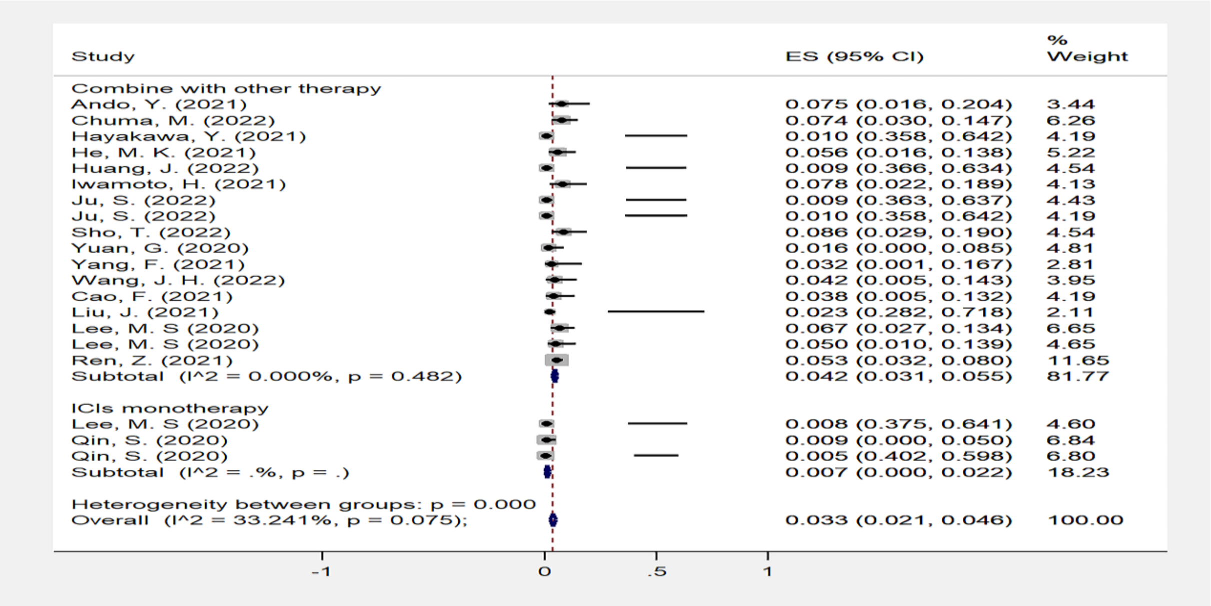

Supplementary Figure S89 Subgroup analysis of the incidence of grade ≥ 3 proteinuria

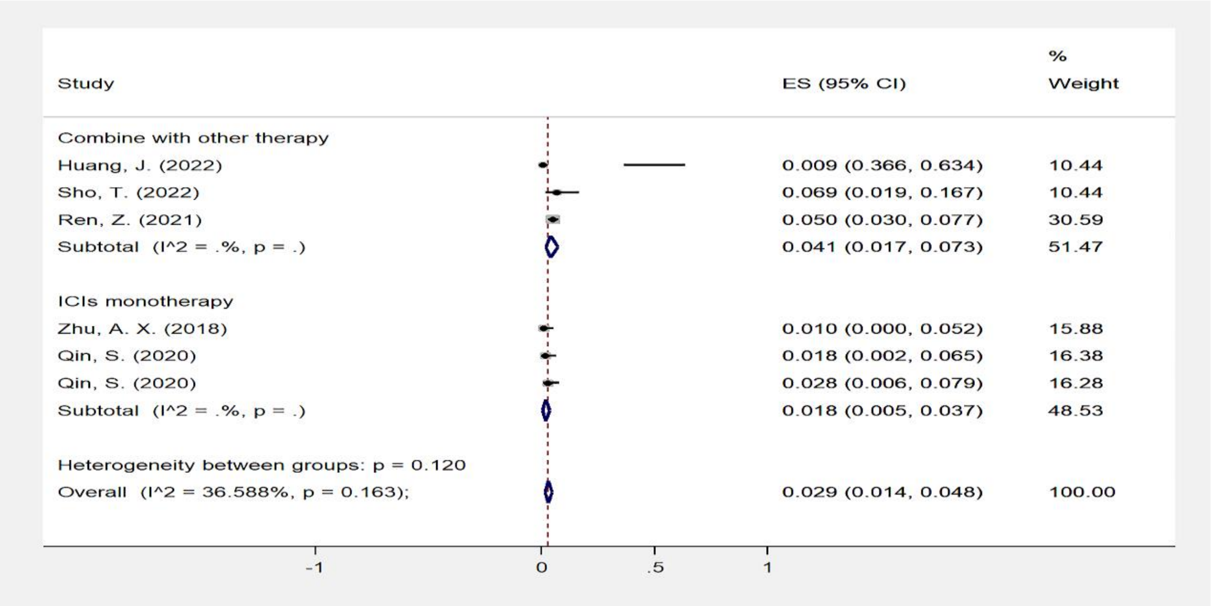

Supplementary Figure S90 Subgroup analysis of the incidence of grade ≥ 3 increased γ-glutamyltransferase

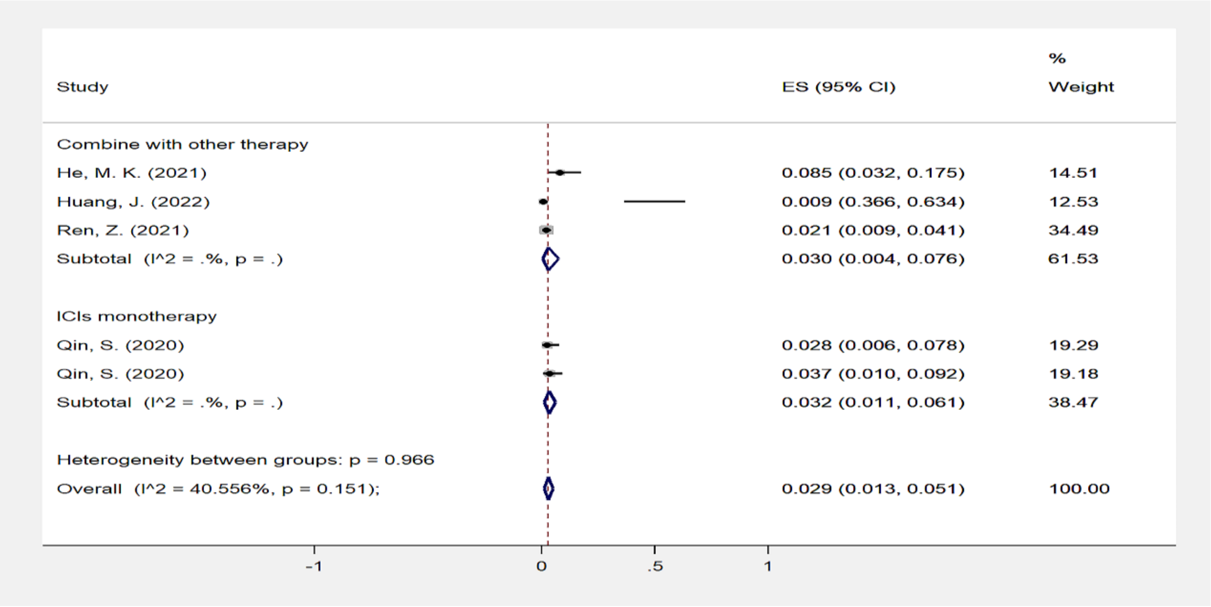

Supplementary Figure S91 Subgroup analysis of the incidence of grade ≥ 3 neutropenia

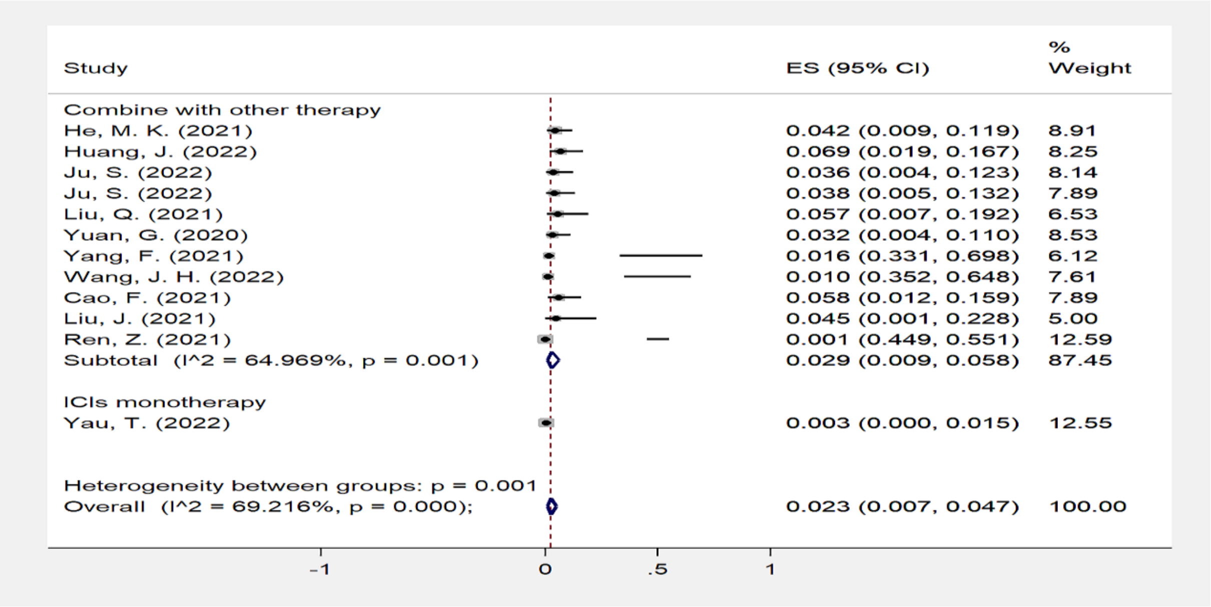

Supplementary Figure S92 Subgroup analysis of the incidence of grade ≥ 3 hand-foot skin reaction

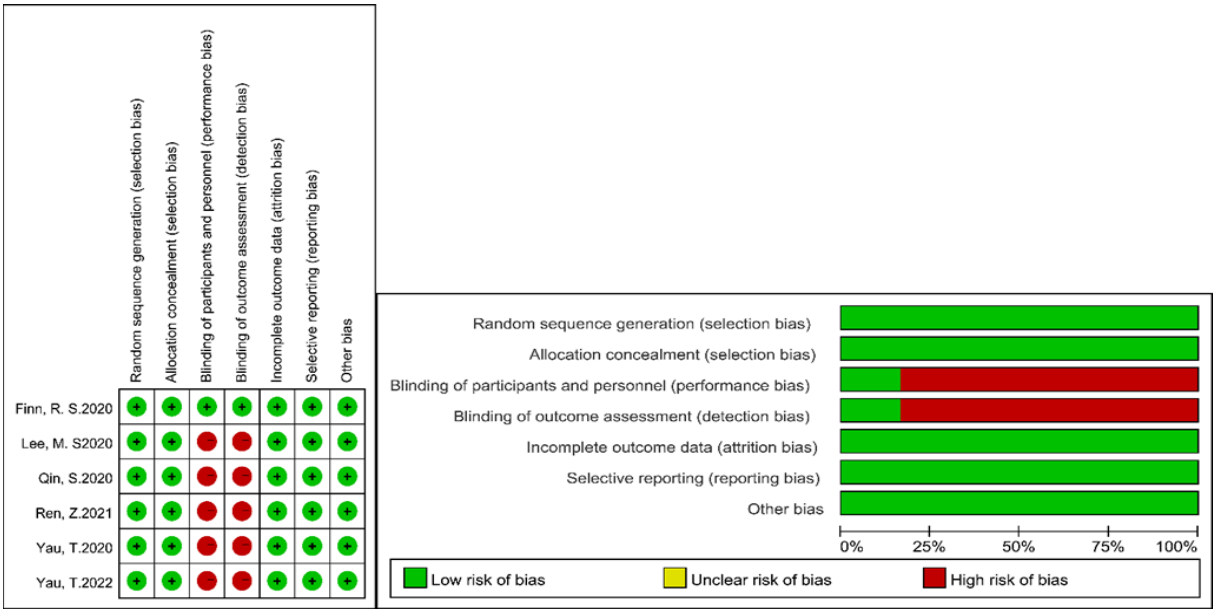

Supplementary Figure S93 Assessment of risk of bias of included RCTs

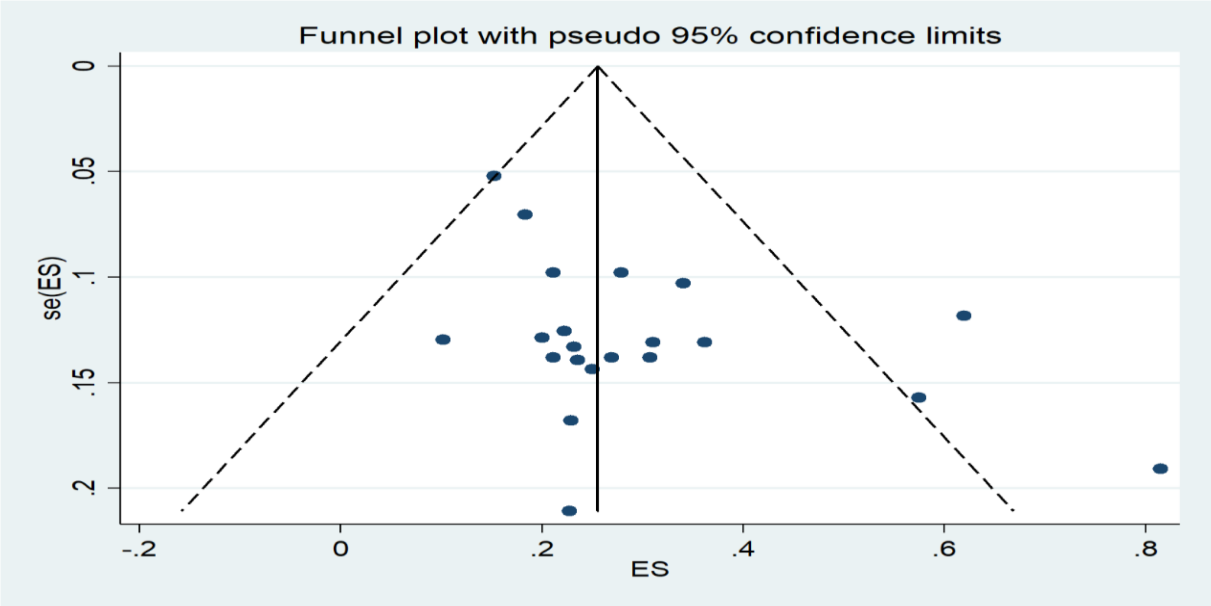

Supplementary Figure S94 Funnel plot for any-grade fatigue

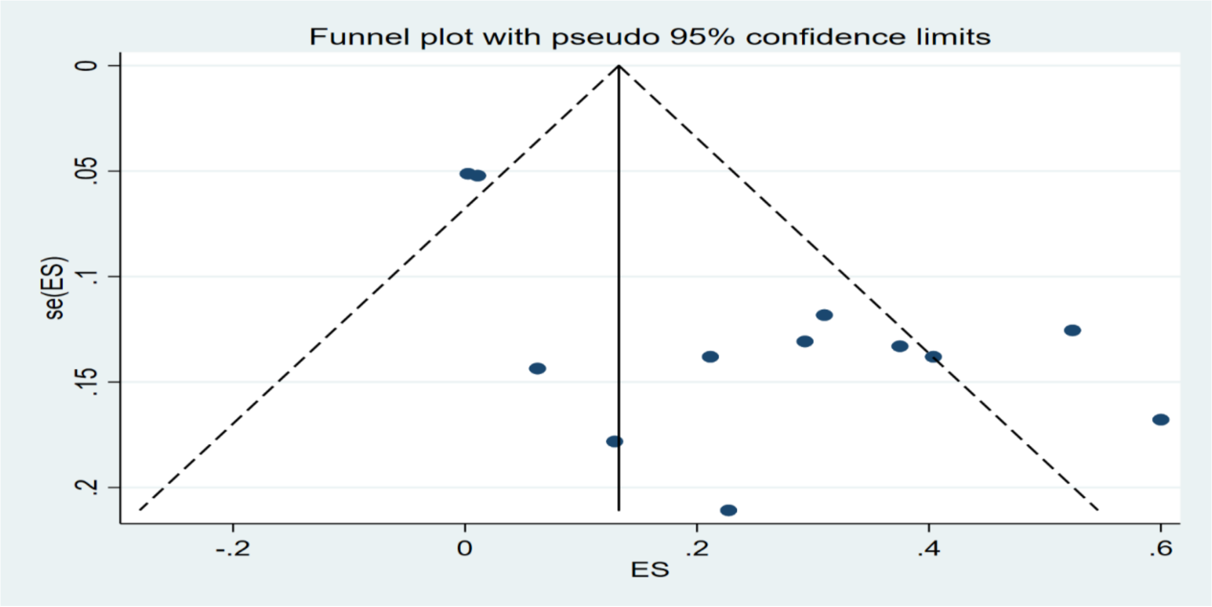

Supplementary Figure S95 Funnel plot for any-grade hand-foot skin reaction

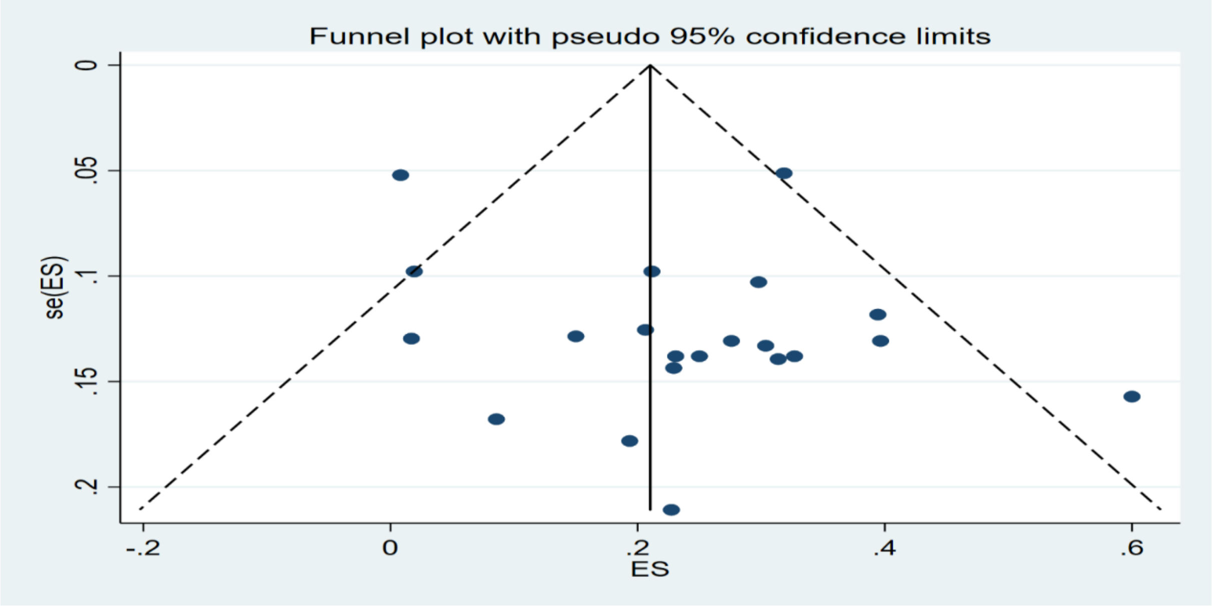

Supplementary Figure S96 Funnel plot for any-grade hypertension

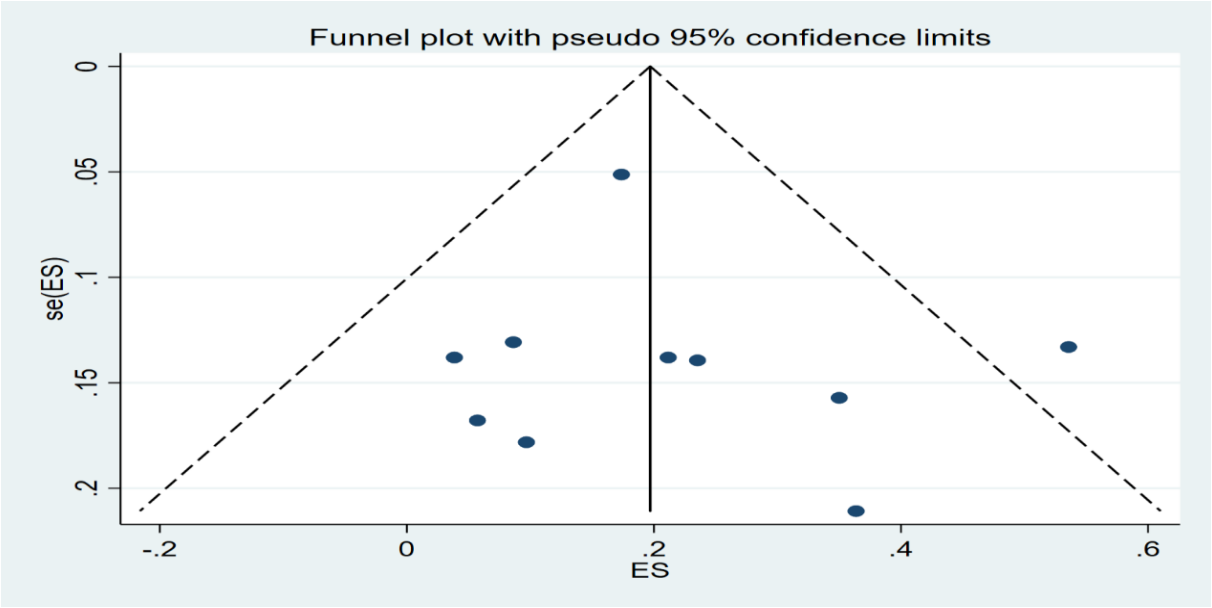

Supplementary Figure S97 Funnel plot for any-grade fever

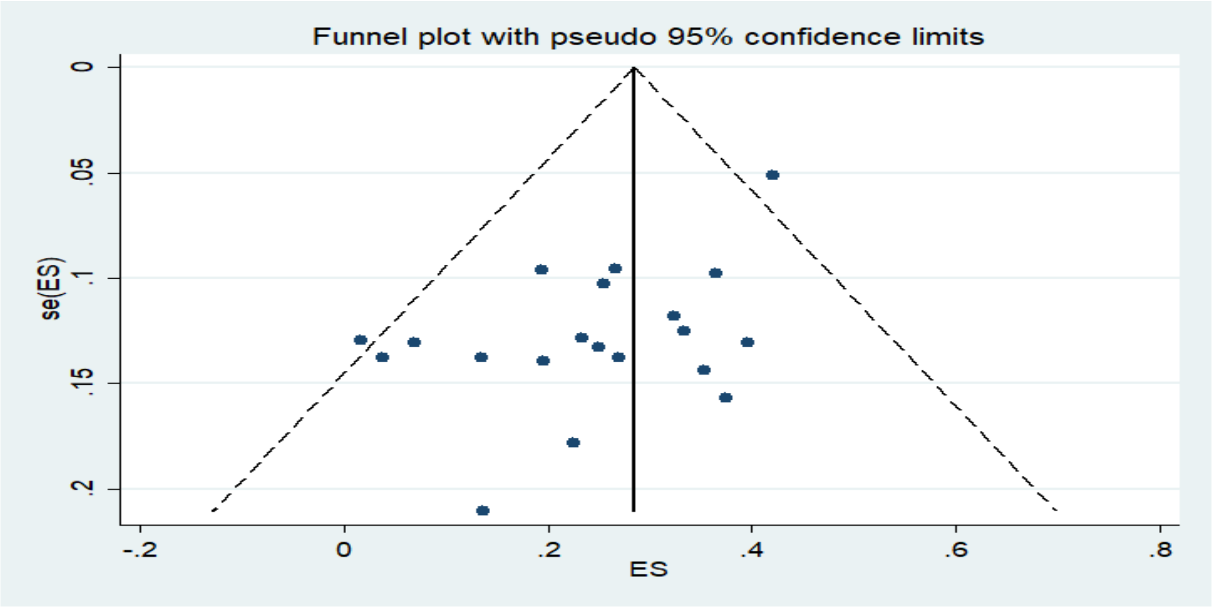

Supplementary Figure S98 Funnel plot for any-grade proteinuria

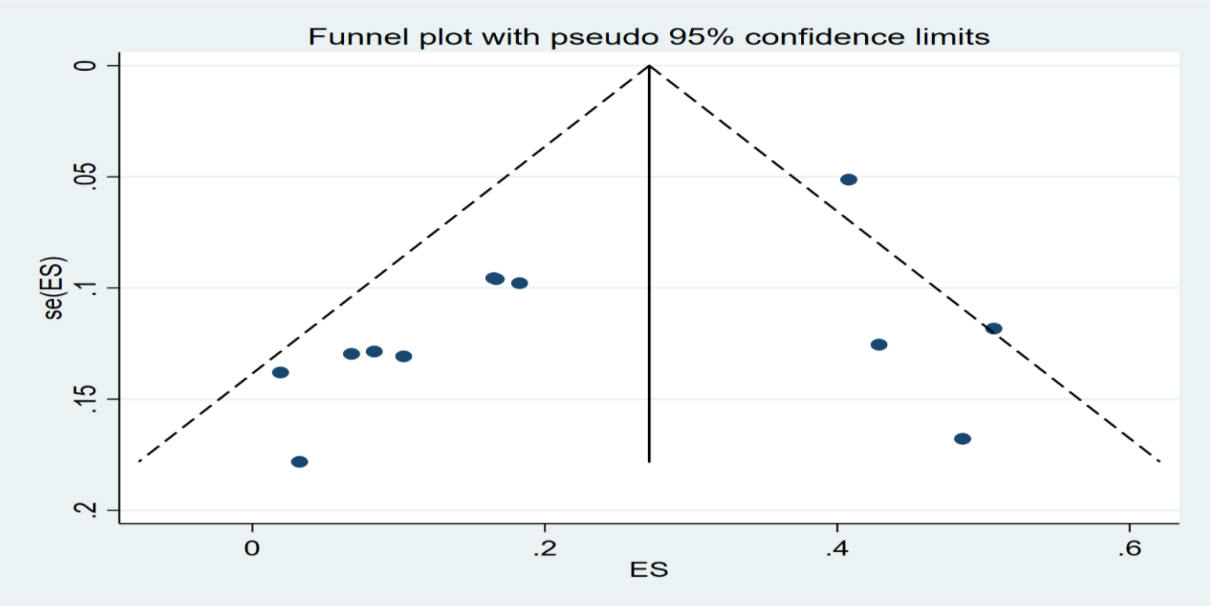

Supplementary Figure S99 Funnel plot for any-grade thrombocytopaenia

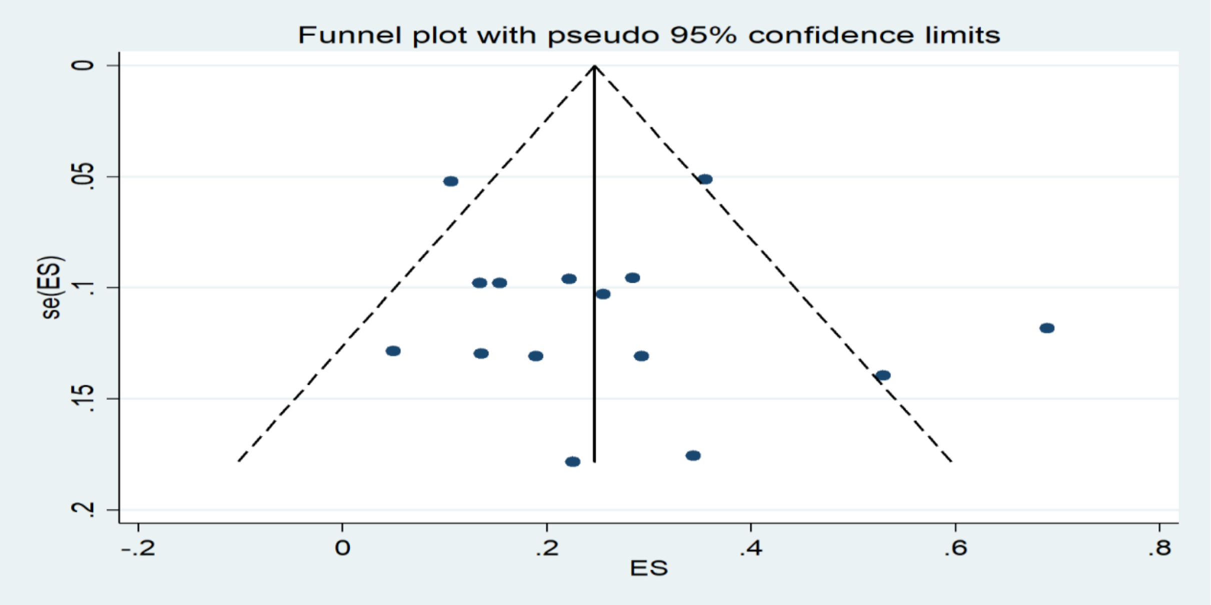

Supplementary Figure S100 Funnel plot for any-grade elevated AST

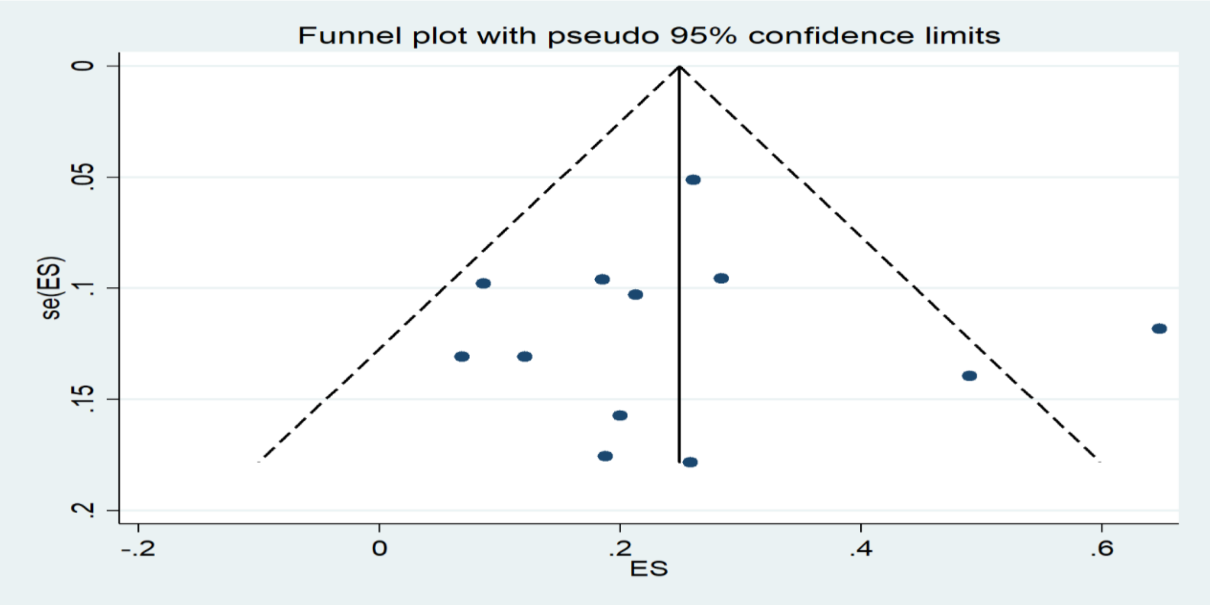

Supplementary Figure S101 Funnel plot for any-grade elevated ALT

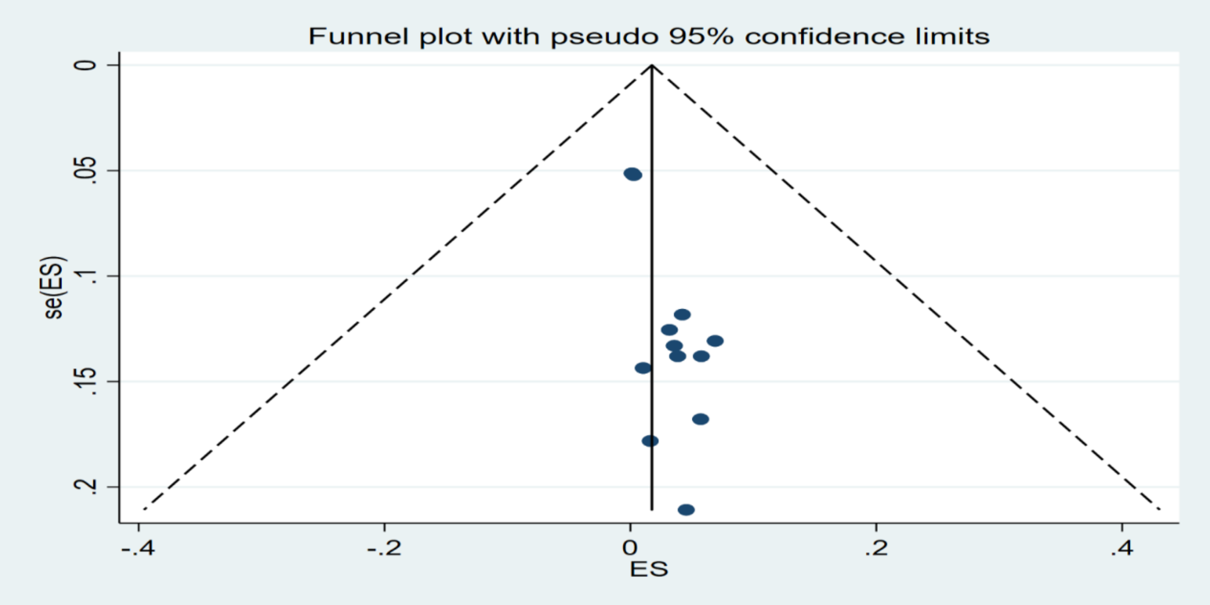

Supplementary Figure S102 Funnel plot for grade ≥ 3 hand-foot skin reaction

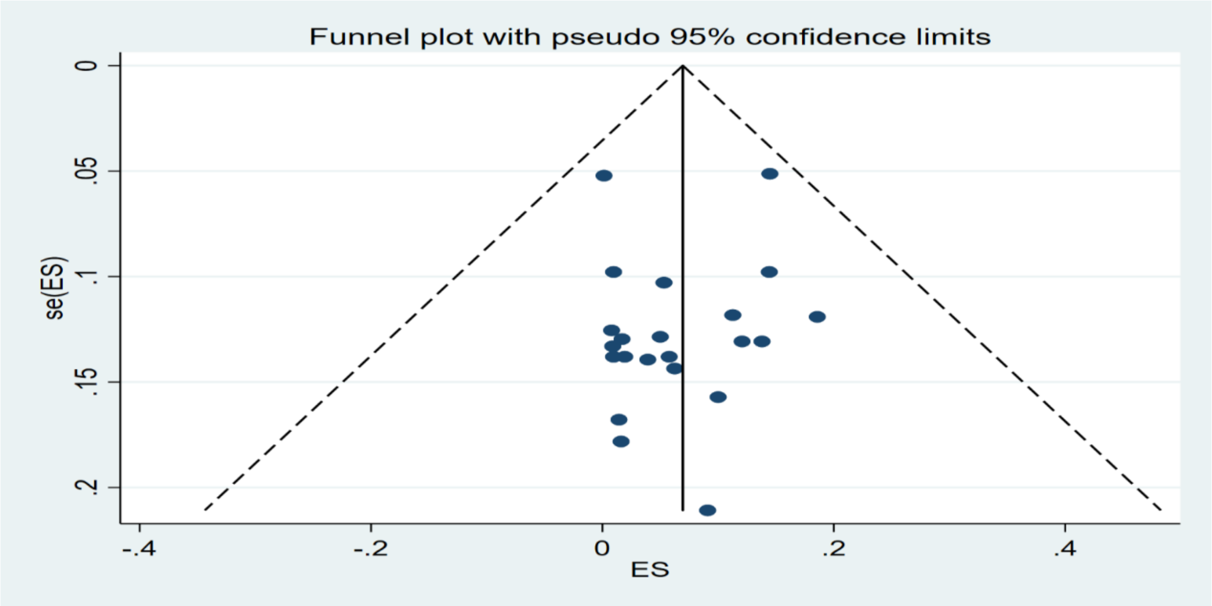

Supplementary Figure S103 Funnel plot for grade ≥ 3 hypertension

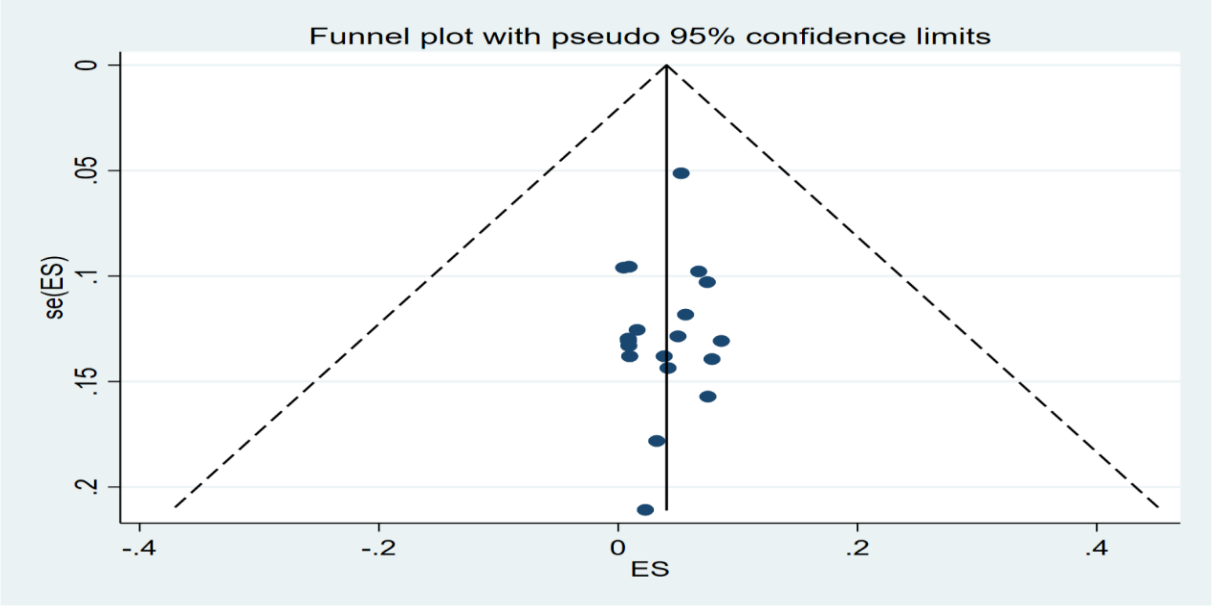

Supplementary Figure S104 Funnel plot for grade ≥ 3 proteinuria

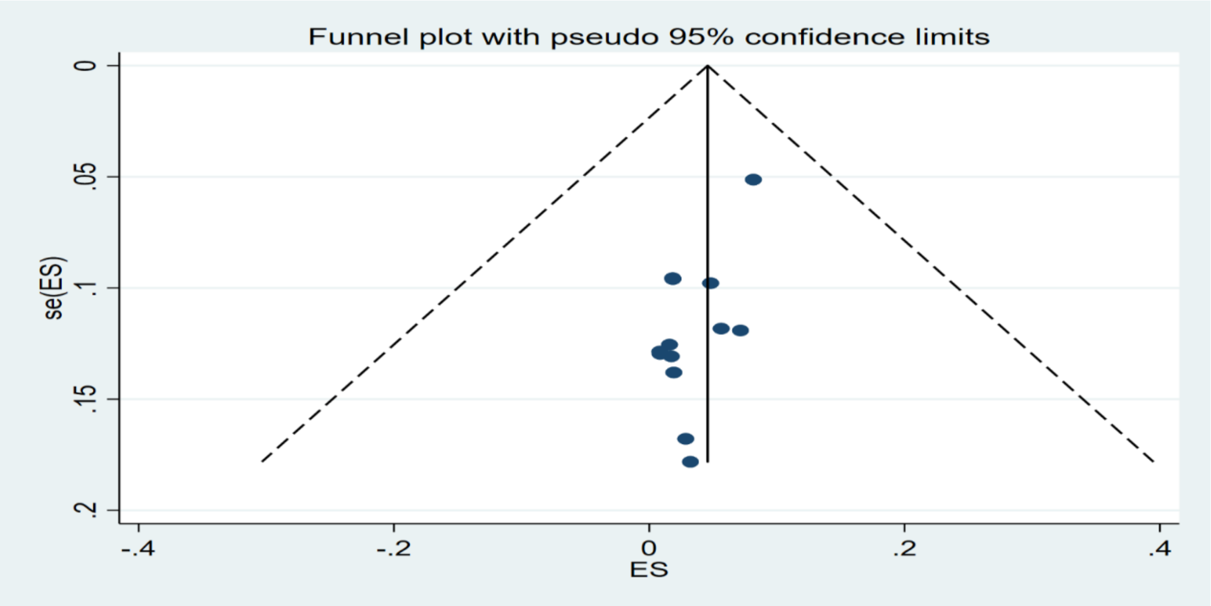

Supplementary Figure S105 Funnel plot for grade ≥ 3 thrombocytopaenia

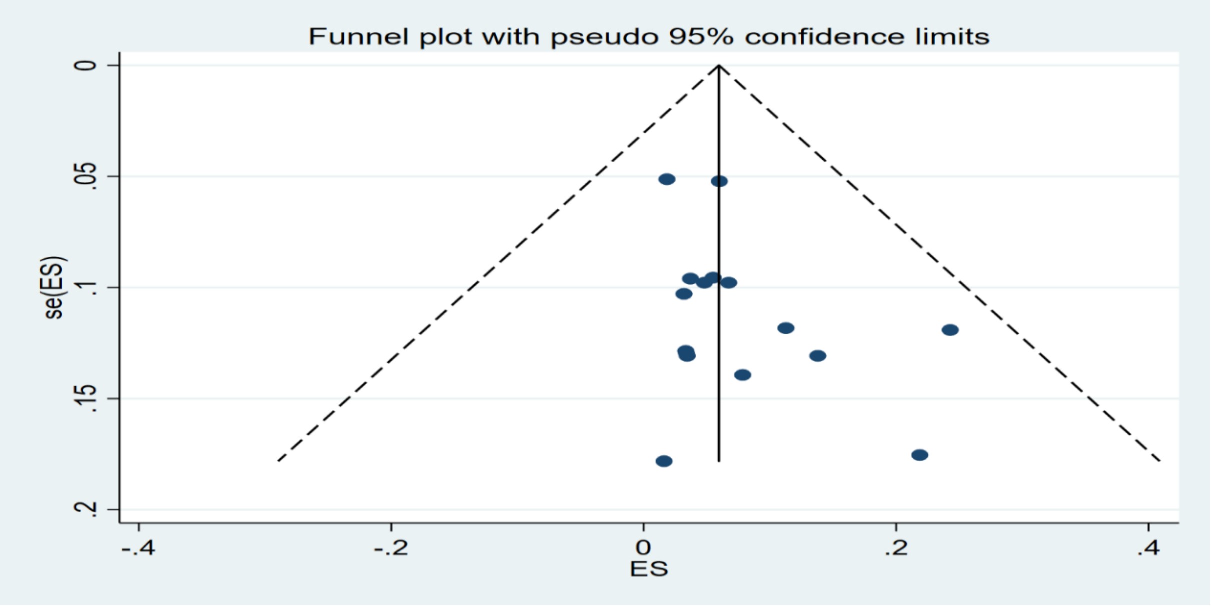

Supplementary Figure S106 Funnel plot for grade ≥ 3 elevated AST

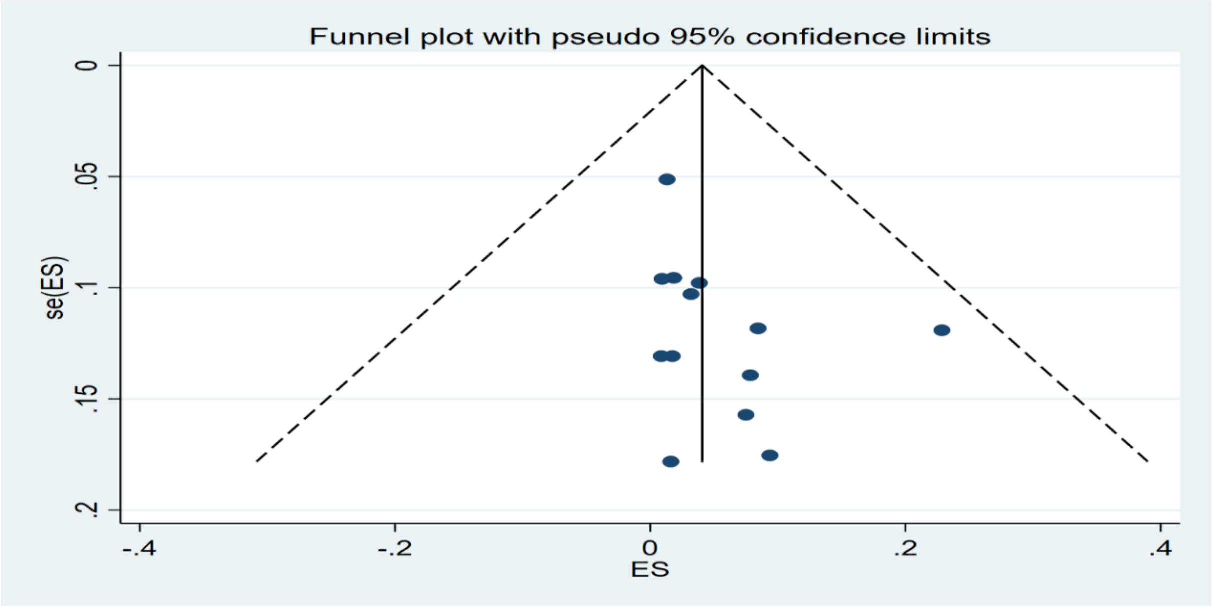

Supplementary Figure S107 Funnel plot for grade ≥ 3 elevated ALT

Supplementary Table S1 Search algorithm and results

pubmed	Searching strategy
#1	"Liver Neoplasms"[Mesh]
#2	((((((((((((((((((((Neoplasms, Hepatic[Title/Abstract]) OR (Neoplasms, Liver[Title/Abstract])) OR (Liver Neoplasm[Title/Abstract])) OR (Neoplasm, Liver[Title/Abstract])) OR (Hepatic Neoplasms[Title/Abstract])) OR (Hepatic Neoplasm[Title/Abstract])) OR (Neoplasm, Hepatic[Title/Abstract])) OR (Cancer of Liver[Title/Abstract])) OR (Hepatocellular Cancer[Title/Abstract])) OR (Cancers, Hepatocellular[Title/Abstract])) OR (Hepatocellular Cancers[Title/Abstract])) OR (Hepatic Cancer[Title/Abstract])) OR (Cancer, Hepatic[Title/Abstract])) OR (Cancers, Hepatic[Title/Abstract])) OR (Hepatic Cancers[Title/Abstract])) OR (Liver Cancer[Title/Abstract])) OR (Cancer, Liver[Title/Abstract])) OR (Cancers, Liver[Title/Abstract])) OR (Liver Cancers[Title/Abstract])) OR (Cancer of the Liver[Title/Abstract])) OR (Cancer, Hepatocellular[Title/Abstract])
#3	#1 OR #2
#4	((((((((immune related adverse event[Title/Abstract]) OR (immune related adverse events[Title/Abstract])) OR (irAEs[Title/Abstract])) OR (irAE[Title/Abstract])) OR (toxicity[Title/Abstract])) OR (safety[Title/Abstract])) OR (adverse event[Title/Abstract])) OR (adverse effect[Title/Abstract])) OR (side effect[Title/Abstract])
#5	"Immune Checkpoint Inhibitors"[Mesh]
#6	((Checkpoint Inhibitors, Immune[Title/Abstract]) OR (Immune Checkpoint Inhibitor[Title/Abstract])) OR (CheckpointInhibitor, Immune[Title/Abstract])) OR (Immune Checkpoint Blockers[Title/Abstract])) OR (Checkpoint Blockers, Immune[Title/Abstract])) OR (Immune Checkpoint Blockade[Title/Abstract])) OR (Checkpoint Blockade, Immune[Title/Abstract])) OR (Immune Checkpoint Inhibition[Title/Abstract])) OR (Checkpoint Inhibition, Immune[Title/Abstract])) OR (PD-L1 Inhibitors[Title/Abstract])) OR (PD L1 Inhibitors[Title/Abstract])) OR (PD-L1 Inhibitor[Title/Abstract])) OR (PD L1 Inhibitor[Title/Abstract])) OR (Programmed Death-Ligand 1 Inhibitors[Title/Abstract])) OR (Programmed Death Ligand 1 Inhibitors[Title/Abstract])) OR (PD-1-PD-L1 Blockade[Title/Abstract])) OR (Blockade, PD-1-PD-L1[Title/Abstract])) OR (PD 1 PD L1 Blockade[Title/Abstract])) OR (CTLA-4 Inhibitors[Title/Abstract])) OR (CTLA 4 Inhibitors[Title/Abstract])) OR (CTLA-4 Inhibitor[Title/Abstract])) OR (CTLA 4 Inhibitor[Title/Abstract])) OR (Cytotoxic T-Lymphocyte-Associated Protein 4 Inhibitors[Title/Abstract])) OR (Cytotoxic T Lymphocyte Associated Protein 4 Inhibitors[Title/Abstract])) OR (Cytotoxic T-Lymphocyte-Associated Protein 4 Inhibitor[Title/Abstract])) OR (Cytotoxic T Lymphocyte Associated Protein 4 Inhibitor[Title/Abstract])) OR (PD-1 Inhibitors[Title/Abstract])) OR (PD 1 Inhibitors[Title/Abstract])) OR (PD-1 Inhibitor[Title/Abstract])) OR (Inhibitor, PD-1[Title/Abstract])) OR (PD 1 Inhibitor[Title/Abstract])) OR (Programmed Cell Death Protein 1 Inhibitor[Title/Abstract])) OR (Programmed Cell Death Protein 1 Inhibitors[Title/Abstract])) OR (pembrolizumab[Title/Abstract])) OR (SCH-900475[Title/Abstract])) OR (lambrolizumab[Title/Abstract])) OR (MK-3475[Title/Abstract])) OR (Keytruda[Title/Abstract])) OR (Nivolumab[Title/Abstract])) OR (Opdivo[Title/Abstract])) OR (ONO-4538[Title/Abstract])) OR (ONO 4538[Title/Abstract])) OR (ONO4538[Title/Abstract])) OR (MDX-1106[Title/Abstract]))

	OR (MDX 1106[Title/Abstract])) OR (MDX1106[Title/Abstract])) OR (BMS-936558[Title/Abstract])) OR (BMS 936558[Title/Abstract])) OR (BMS936558[Title/Abstract])) OR (atezolizumab[Title/Abstract])) OR (anti-PDL1[Title/Abstract])) OR (immunoglobulin G1, anti-(human CD antigen CD274) (human monoclonal MDPL3280a heavy chain), disulfide with human monoclonal MDPL3280a kappa-chain, dimer[Title/Abstract])) OR (MPDL3280A[Title/Abstract])) OR (MPDL-3280A[Title/Abstract])) OR (Tecentriq[Title/Abstract])) OR (RG7446[Title/Abstract])) OR (RG-7446[Title/Abstract])) OR (durvalumab[Title/Abstract])) OR (MEDI4736[Title/Abstract])) OR (MEDI-4736[Title/Abstract])) OR (Imfinzi[Title/Abstract])) OR (avelumab[Title/Abstract])) OR (MSB-0010682[Title/Abstract])) OR (MSB0010682[Title/Abstract])) OR (bavencio[Title/Abstract])) OR (MSB0010718C[Title/Abstract])) OR (MSB-0010718C[Title/Abstract])) OR (cemiplimab[Title/Abstract])) OR (REGN2810[Title/Abstract])) OR (dostarlimab-gxly[Title/Abstract])) OR (Jemperli[Title/Abstract])) OR (Ipilimumab[Title/Abstract])) OR (Anti-CTLA-4 MAb Ipilimumab[Title/Abstract])) OR (Anti CTLA 4 MAb Ipilimumab[Title/Abstract])) OR (Ipilimumab, Anti-CTLA-4 MAb[Title/Abstract])) OR (Yervoy[Title/Abstract])) OR (MDX 010[Title/Abstract])) OR (MDX010[Title/Abstract])) OR (MDX-010[Title/Abstract])) OR (MDX-CTLA-4[Title/Abstract])) OR (MDX CTLA 4[Title/Abstract])) OR (toripalimab[Title/Abstract])) OR (sintilimab[Title/Abstract])) OR (IBI 308[Title/Abstract])) OR (IBI308[Title/Abstract])) OR (IBI-308[Title/Abstract])) OR (camrelizumab[Title/Abstract])) OR (carrelizumab[Title/Abstract])) OR (SHR-1210[Title/Abstract])) OR (SHR 1210[Title/Abstract])) OR (tislelizumab[Title/Abstract])) OR (BGB-A317[Title/Abstract])
#7	#5 OR #6
#8	#3 AND #4 AND #7

Supplementary Table S2 Risk of bias and quality assessment of cohort studies included using NOS (n=16)

Study	Selection 1	Selection 2	Selection 3	Selection 4	Comparability	Outcomes 1	Outcomes 2	Outcomes 3	Scores
Ando, Y.	1	1	1	1	1	1	0	0	6
Chen, S.	1	1	1	1	2	1	1	0	8
Chuma, M.	1	1	1	1	2	1	0	0	7
D'Alessio, A.	1	1	1	0	1	1	1	0	6
de Castro, T.	1	1	1	0	2	1	1	1	8
Guo, Y.	1	1	1	0	1	1	1	0	6
Hayakawa, Y.	1	1	1	0	1	1	1	0	6
He, M. K.	1	1	1	0	2	1	0	0	6
Huang, J.	1	1	1	0	1	1	0	0	5
Iwamoto, H.	1	1	1	0	2	1	0	0	6
Ju, S.	1	1	1	0	1	1	1	0	6
Liu, Q.	1	1	1	0	1	1	1	0	6
Sho, T.	1	1	1	0	1	1	0	0	5
Xia, J.	1	1	1	0	2	1	0	0	6
Yuan, G.	1	1	1	0	1	1	1	0	6
Zhang, S.	1	1	1	0	1	1	1	1	7

Supplementary Table S3 Risk of bias and quality assessment of non-randomized studies included using MINORS (n=7)

Study	1	2	3	4	5	6	7	8	9	10	11	12	TOALE
Yang, F.	2	2	2	2	1	2	0	0	-	-	-	-	11
Kudo, M.	2	2	2	2	1	2	0	0	-	-	-	-	11
Zhu, A. X.	2	2	2	2	1	2	0	0					11
Liu, J.	2	2	2	2	1	0	0	0	-	-	-	-	9
Cao, F.	2	2	2	2	1	2	0	0					11
Wang, J. H.	2	3	2	2	1	2	0	0	-	-	-	-	12
Duffy, A. G.	2	2	2	2	2	1	0	0	2	2	2	2	19
